# Synthesis of 1,3-Disubstituted 3‑Azabicyclo[3.2.0]heptane Libraries for Fragment-Based Drug Discovery

**DOI:** 10.1021/acs.orglett.6c02332

**Published:** 2026-07-13

**Authors:** Mathilde A. C. H. Janssen, Jesper W. A. van der Vorm, Rico Rappard, Bart Bijleveld, Tom Dekker, Maikel Wijtmans, Iwan J. P. de Esch, Daniel Blanco-Ania, Floris P. J. T. Rutjes

**Affiliations:** † Institute for Molecules and Materials, 6029 Radboud University, Heyendaalseweg 135, 6525 AJ Nijmegen, the Netherlands; ‡ Amsterdam Institute of Molecular and Life Sciences, 159203 Vrije Universiteit Amsterdam, De Boelelaan 1108, 1081 HZ Amsterdam, The Netherlands

## Abstract

The 3-azabicyclo[3.2.0]­heptane scaffold, yet underexplored in drug discovery, offers significant potential as a structural motif for medicinal chemistry. In this study, we present the synthesis of 1,3-disubstituted 3-azabicyclo[3.2.0]­heptane derived building blocks via a 1,3-dipolar cycloaddition between the unique cyclobutene-1-sulfonyl fluoride (CBSF) building block and an azomethine ylide. To evaluate the chemical diversity and novelty of the fragments, we synthesized a covalent and a noncovalent fragment library inspired by an automated workflow. The synthesis and derivatization processes—including the formation of sulfonamides, carboxamides, ureas, and tertiary amines—are detailed, demonstrating the versatility and synthetic potential of 3-azabicyclo[3.2.0]­heptane scaffolds in drug discovery.

## Graphical abstract

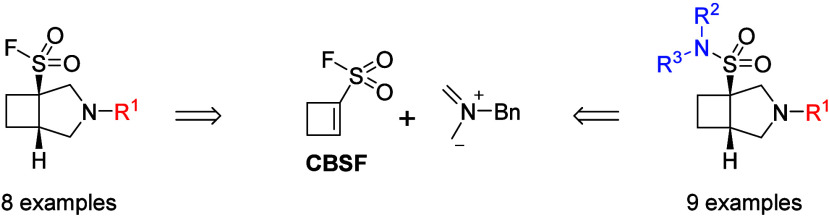

The identification of new small-molecule scaffolds remains essential for progress in drug discovery and the development of innovative therapeutics. Although the 3-azabicyclo[3.2.0]­heptane framework is not widely explored in medicinal chemistry, it appears in several biologically active compounds, such as the antipsychotic belaperidone and the antibacterial agent ecenofloxacin (Figure 1).^1,2^ This structural motif can be regarded as a conformationally constrained isostere of piperazine as well as 3-substituted pyrrolidines and piperidines, which may lead to improved binding affinity and selectivity.^3,4^

**Figure 1:**
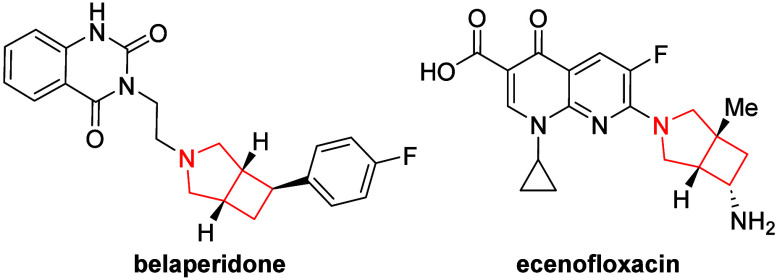
Biologically active compounds containing the 3-azabicyclo[3.2.0]­heptane scaffold: belaperidone and ecenofloxacin.

To further broaden the chemical space associated with this scaffold, we report the synthesis of 1,3-disubstituted 3-azabicyclo[3.2.0]­heptane derivatives **1** and **2**. These compounds were prepared through a 1,3-dipolar cycloaddition involving the cyclobutene-1-sulfonyl fluoride (**CBSF**, **4**) building block and an azomethine ylide, affording bicyclic intermediate **3** (Scheme 1).^3,5,6^ This intermediate was subsequently functionalized to yield fragments **1** and **2**, enabling evaluation of their diversity in fragment-based drug discovery (FBDD) studies.^7^ Fragments **1** are suited for irreversible (covalent) inhibition, whereas fragments **2** are designed for reversible (noncovalent) inhibition.

**Scheme 1:**
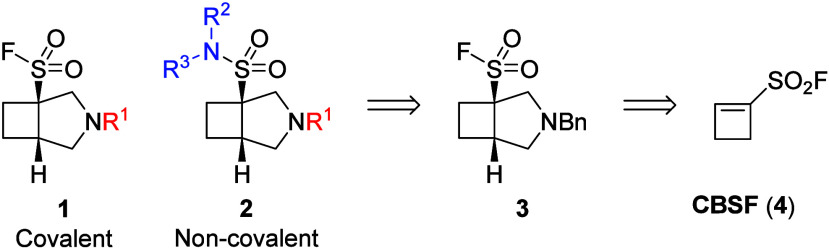
Retrosynthetic Approach to 3-Azabicyclo[3.2.0]­heptane Fragments **1** and **2**

Sulfonyl fluorides have recently emerged as valuable electrophilic groups for use as covalent warheads in chemical biology and drug discovery.^8–10^ These functionalities combine an optimal balance between biocompatibility (unreactive to water) and protein reactivity.^11,12^ In contrast to commonly employed warheads such as acrylamide, sulfonyl fluorides can react with a broader range of amino acid residues, including serine, threonine, lysine, and histidine, in addition to cysteine.^13^ This broader reactivity profile renders them promising moieties in the development of new covalent inhibitors.

In this study, both covalent and noncovalent fragment libraries based on the 3-azabicyclo[3.2.0]­heptane scaffold were synthesized from sulfonyl fluoride intermediate **3**. The substituents introduced in the covalent fragment set were selected using an automated open-source KNIME workflow developed by Dekker et al.^14^ This workflow evaluates physicochemical properties, structural diversity, novelty, and three-dimensional features, enabling the identification of fragments that efficiently cover underexplored regions of chemical space.

The synthesis of these libraries commenced with the preparation of cyclobutane-1-sulfonyl fluorides **5a** and **5b** as a 1:1 *cis*/*trans* mixture (91% yield), following a previously described protocol (Scheme 2).^15^ The key [2 + 2] cycloaddition reaction was carried out under high-pressure conditions (12 kbar) using ethenesulfonyl fluoride (ESF, **6**) and *tert*-butyl vinyl ether.

**Scheme 2:**
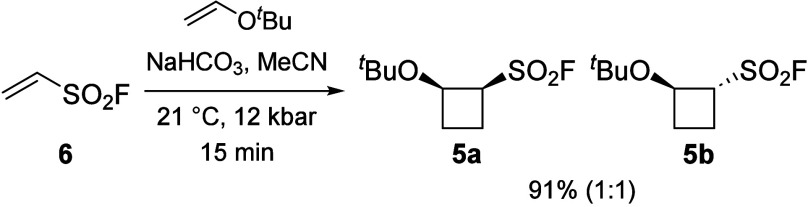
Synthesis of Cyclobutanes **5a** and **5b** via a Hyperbaric [2 + 2] Cycloaddition

This stereoisomeric mixture was subsequently converted into **CBSF** (**4**) via a three-step sequence: (1) de-*tert*-butylation (BBr_3_, CH_2_Cl_2_, 21 °C, 2.5 h) to form the corresponding alcohols **7a** and **7b**, (2) triflation under standard conditions (Tf_2_O, pyridine, CH_2_Cl_2_, 21 °C, 2 h) to yield the *cis*/*trans* mixture of triflates **8a** and **8b** (55% yield over two steps), and (3) elimination (KO^*t*^Bu, CH_2_Cl_2_, 21 °C, 3 h) to generate cyclobutene **4** (Scheme 3). The crude alcohol mixture (**7a** and **7b**) was obtained by simple quenching with MeOH, followed by solvent removal and used directly. Attempts to separate the *cis*- and *trans*-isomers through column chromatography led to ring opening, producing aldehyde **9** via a retro-aldol-type process. Purification of **CBSF** was achieved by vacuum distillation; although traces of THF remained, this did not affect subsequent transformations, and a stock solution of **CBSF** in THF was used directly in the next steps.

**Scheme 3:**
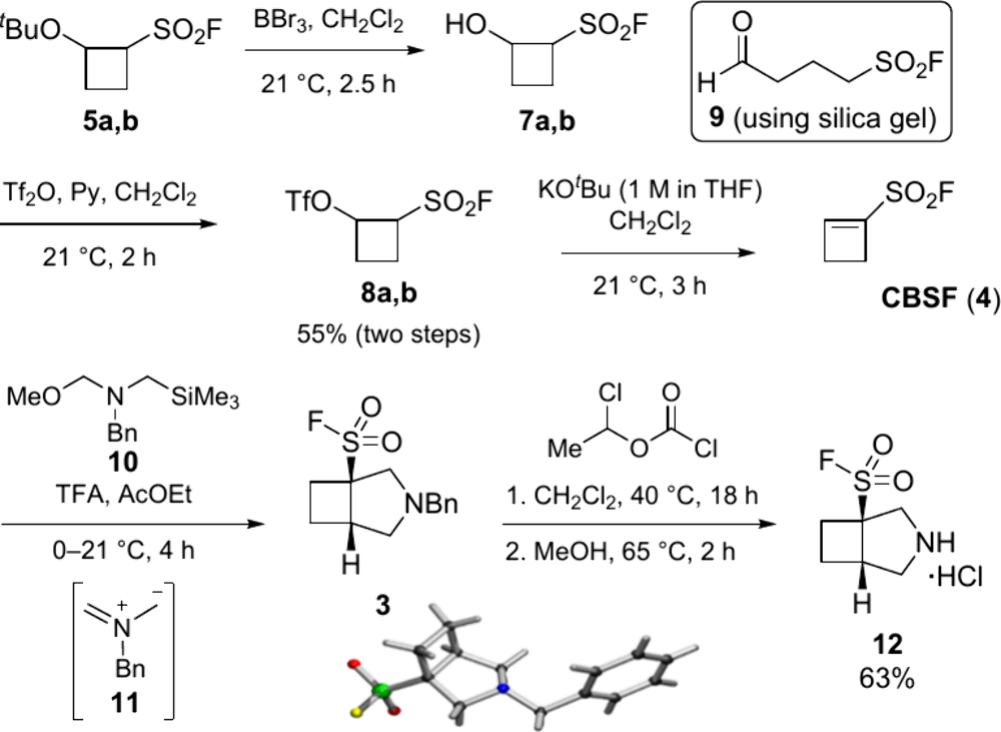
Synthesis of **CBSF** (**4**) and Bicyclosulfonyl Fluoride **3** (Including Its X-ray Structure) and **12**

The next step involved a 1,3-dipolar cycloaddition between **CBSF** and the *in situ* generated ylide **11**, derived from *N*-(methoxymethyl)-*N*-[(trimethylsilyl)­methyl]­benzylamine (**10**)^5^ providing bicyclosulfonyl fluoride **3** in 75% yield (Scheme 3). The structure of bicyclic compound **3** was confirmed by X-ray diffraction.

Key intermediate **3** was first modified at the nitrogen atom by debenzylation using 1-chloroethyl chloroformate, initially giving a carbamate intermediate that was subsequently deprotected to yield pyrrolidine sulfonyl fluoride **12** as its hydrochloride salt (Scheme 3).^16^

Scaffold **12** was further derivatized under standard conditions to give a series of amides, ureas, and tertiary amines (Scheme 4). Carboxamides **1a** and **1b** were obtained in good yields (83 and 74%, respectively) using the corresponding acyl chlorides and triethylamine. The pyrazole-derived amide **1c** was formed in moderate yield (50%), while the thiazole analogue **1d** was isolated in a lower yield (25%),^17^ likely due to incomplete conversion even after extended heating at 40 °C. Treatment of pyrrolidine **12** with ethyl and phenyl isocyanates provided ureas **1e** and **1f** in high isolated yields of 84 and 74%, respectively.^18^ Tertiary amines **1g** and **1h** were synthesized by alkylation with the corresponding alkyl bromides.^19^ Although full conversion was achieved for **1g**, the isolated yield was rather limited (3%). Attempts to obtain tertiary amines **1i** via reductive amination with oxetan-3-one^20^ and **1j** via Buchwald–Hartwig coupling were unsuccessful, although starting material could be recovered in both cases.

**Scheme 4:**
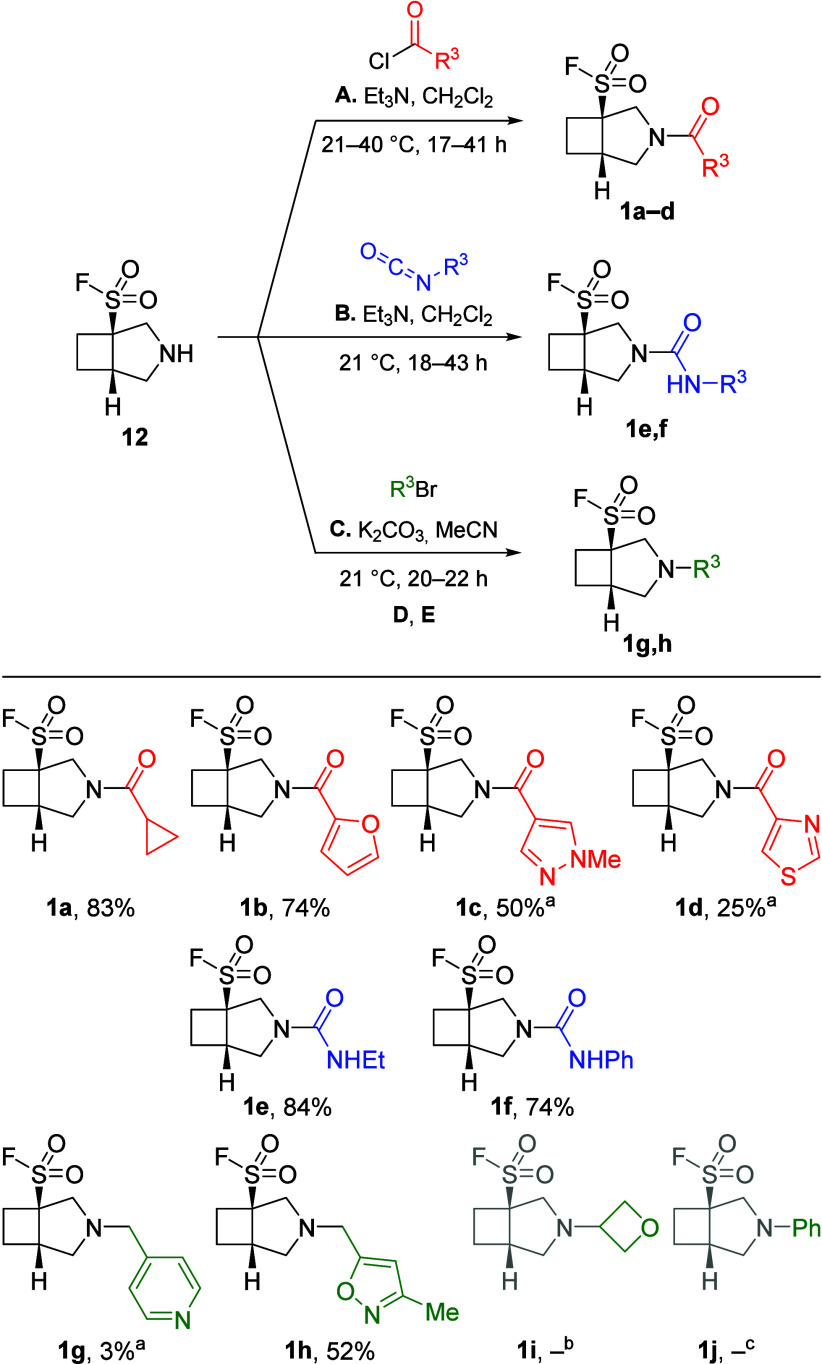
Synthesis of Amides (**1a**–**d**), Ureas (**1e** and **1f**), and Tertiary Amines (**1g** and **1h**) from Pyrrolidine **12**

a Yield after preparative HPLC.
b Method D: oxetan-3-one, NaBH­(OAc)_3_, AcOH, DIPEA, THF, 21 °C.
c Method E: NaO*^t^* Bu, Pd­(OAc)_2_, RuPhos, PhBr, THF, 70 °C.

With the covalent fragment library **1a**–**h** established, attention shifted to transforming the sulfonyl fluoride group into sulfonamides via reaction with various amines. Several literature procedures were screened using different amines (e.g., pyrrolidine, cyclopropanamine, methanamine, diethylamine, and aniline) to optimize this transformation (see Supporting Information for the optimization process): (1) DBU or Et_3_N/DMAP in PhMe or MeOH or MeCN at 110–150 °C for 4–48 h,^15,21^ (2) Ca­(NTf_2_)_2_ and DABCO in THF at 21 °C and 1–15000 bar for 16–72 h (methodology described by Mahapatra et al.),^22^ and (3) HOBT and DIPEA at 21 °C for 24 h (conditions described by Wei et al.).^23^ Based on these experiments, the Ca­(NTf_2_)_2_/DABCO methodology under hyperbaric conditions was selected for further use.

Under these conditions, both primary (aliphatic and aromatic) and secondary amines (cyclic and acyclic) afforded the corresponding sulfonamides in yields ranging from low to moderate (6–72%) (Scheme 5). Although conversions were not always complete, the reactions proceeded cleanly and unreacted starting material could be recovered. Primary aliphatic amines gave the best results, whereas aromatic and secondary amines showed lower efficiency. Consequently, sulfonamides **13a–c** were selected for further derivatization.

**Scheme 5:**
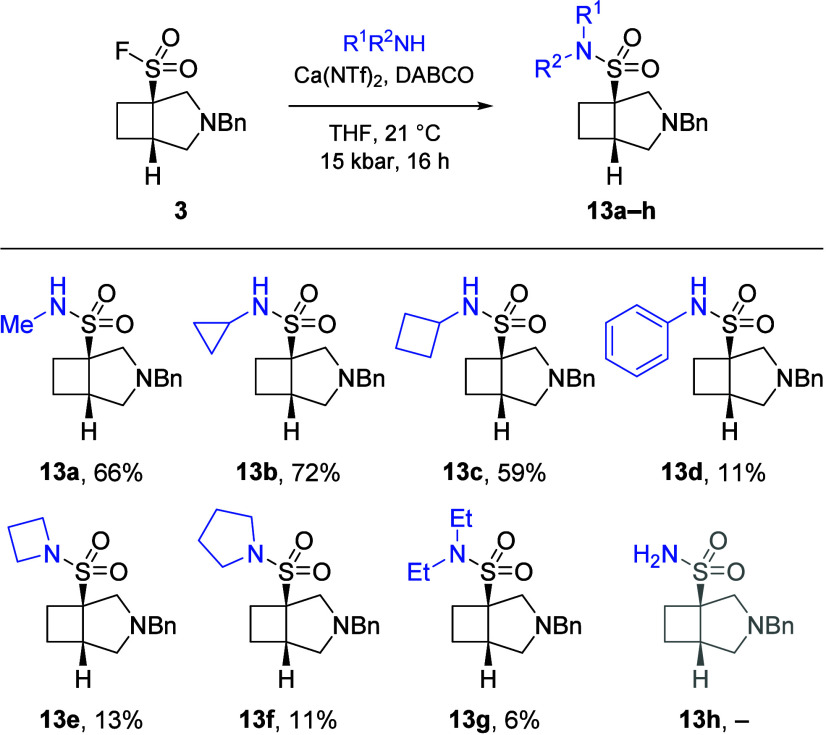
Sulfonamide Formation from Sulfonyl Fluoride **3**

Debenzylation of benzyl-protected sulfonamides **13a**–**c** using chloroethyl chloroformate afforded pyrrolidines **14a**–**c** as their hydrochloride salts (Scheme 6).^16^ Pyrrolidines **14a** and **14b** were purified by trituration, providing yields of 55 and 77%, respectively, while **14c** was used crude due to difficulties encountered during purification.

**Scheme 6:**
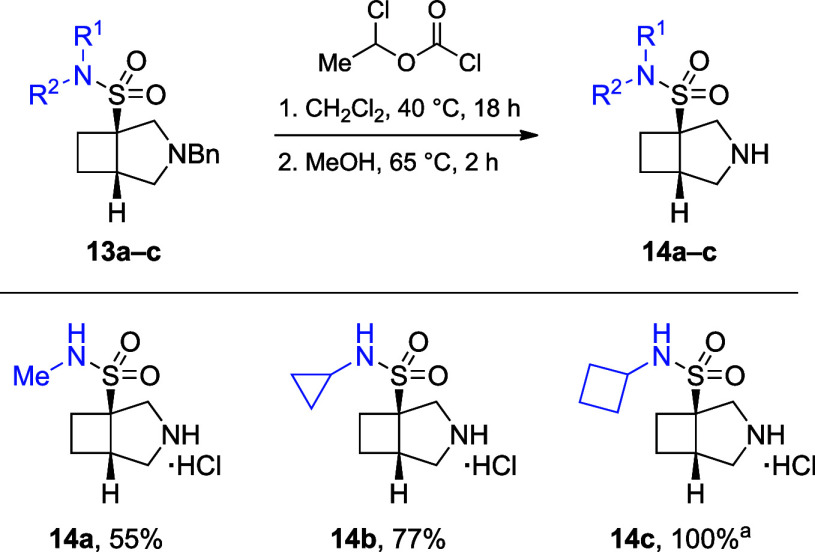
Debenzylation of Benzylamino Sulfonamides **13a**–**c** to Give Pyrrolidines **14a**–**c**

a Crude yield.

Subsequent functionalization of pyrrolidines **14a**–**c** provided amides, ureas and tertiary amines (Scheme 7). Amide **2aa** was obtained from pyrrolidine **14a** and benzoyl chloride in 48% yield,^16^ whereas amides **2bb** and **2cc** were prepared from **14b** and **14c**, giving yields of 90 and 49%, respectively. Ureas **2ad**, **2be**, and **2cf** were synthesized using isocyanates or carbamate-based reagents, affording moderate yields.^18^ Tertiary amines were accessed via multiple approaches: (1) Buchwald–Hartwig coupling of pyrrolidine **14a** with 3-bromoanisole furnished **2ag** (62% yield),^24^ (2) reductive amination of **14b** with cyclohexanone afforded **2bh** (54% yield),^20^ and (3) nucleophilic substitution of **14c** with 2-bromoacetic acid produced **2ci** (27% yield),^19^ although purification proved challenging due to of zwitterion formation.

**Scheme 7:**
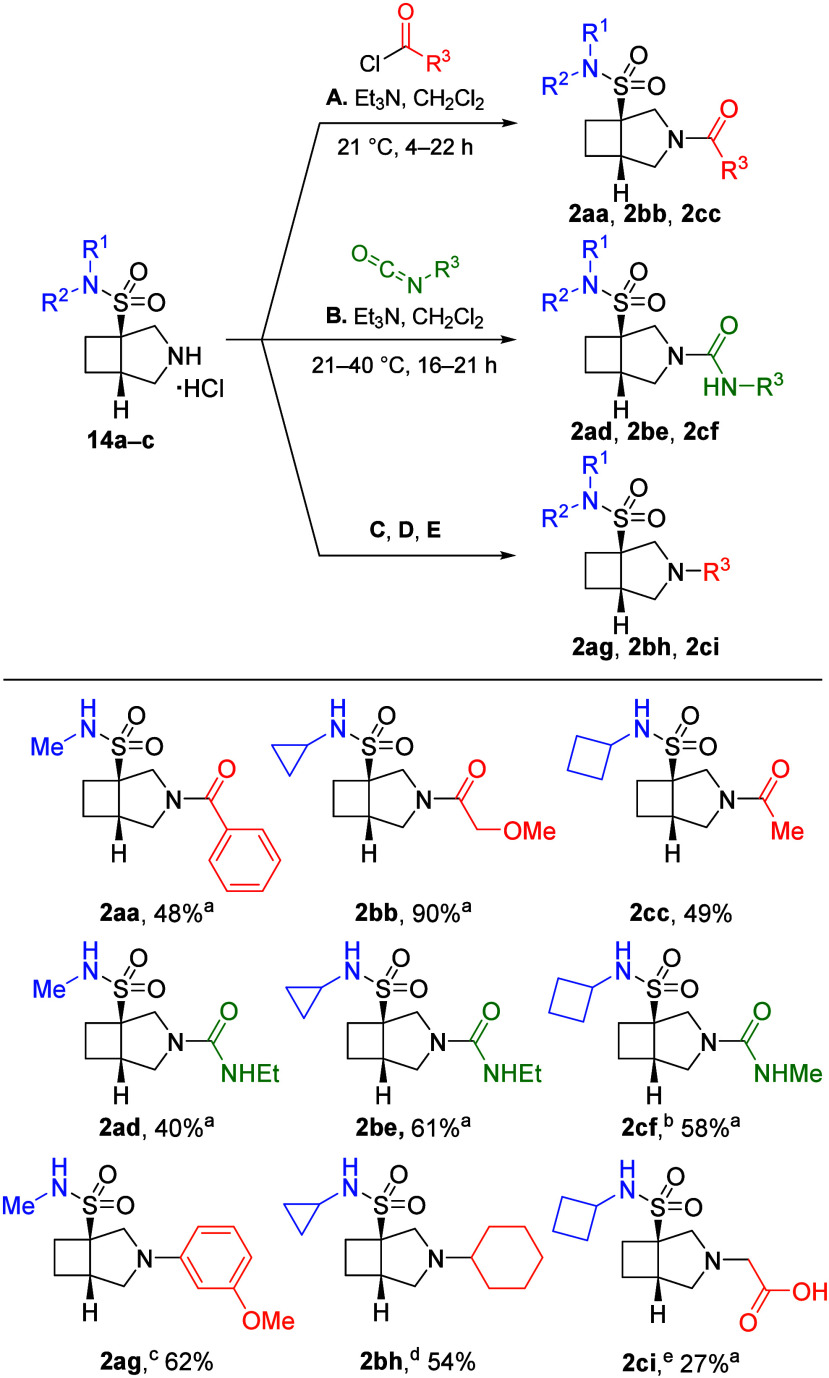
Derivatization of Sulfonamides **14a**–**c** into Amides **2aa**, **2bb**, and **2cc**, Ureas **2ad**, **2be**, and **2cf**, and Tertiary Amines **2ag**, **2bh**, and **2ci**

a Yield after preparative HPLC.
b Succinimidyl *N*-methylcarbamate was used instead of MeNCO.
c Method C: 3-BrC_6_H_4_OMe, NaO*^t^* Bu, Pd­(OAc)_2_, RuPhos, THF, 70 °C, 20 h.
d Method D: cyclohexanone, NaBH­(OAc)_3_, AcOH, DIPEA, THF, 21 °C, 24 h.
e Method E: BrCH_2_CO_2_H, DIPEA, THF, 21 °C, 24 h.

In summary, we have developed an efficient synthetic route to a novel library of 1,3-disubstituted 3-azabicyclo[3.2.0]­heptane derivatives. The strategy involves a high-pressure-promoted [2 + 2] cycloaddition to access the cyclobutene-1-sulfonyl fluoride building block, followed by a 1,3-dipolar cycloaddition to generate a versatile bicyclic intermediate. From this intermediate, two complementary fragment libraries were prepared: a covalent set retaining the sulfonyl fluoride functionality, and a noncovalent set in which it is converted into sulfonamides, both further diversified into amides, ureas, and tertiary amines. To ensure broad coverage of chemical space, fragment selection was guided by an open-source workflow evaluating physicochemical and structural properties. This approach produced a collection of compounds with pronounced three-dimensional character and high novelty, making them suitable for fragment-based screening. Ongoing studies are assessing their biological activity against histamine (H_1_R, H_3_R, H_4_R) and chemokine (ACKR3, CXCR4) receptors, which are relevant to inflammatory and neurological diseases. In addition, further work is underway to explore the reactivity and broader synthetic applicability of the **CBSF** building block.

## Supplementary Material



## Data Availability

The data underlying this study are available in the published article and its Supporting Information.

## Notes

Experimental data, NMR characterizations, and XRD analysis (PDF)
