# Supplementary material for: Synthesis of 1,3-Disubstituted 3‑Azabicyclo[3.2.0]heptane Libraries for Fragment-Based Drug Discovery
Source: Org Lett. 2026 Jul 13;28(29):9239–43. doi: 10.1021/acs.orglett.6c02332 (PMC13411048; doi:10.1021/acs.orglett.6c02332)
Supplement: Supplementary file 1 [file ol6c02332_si_001.pdf]

Synthesis of 1,3-Disubstituted 3-Azabicyclo[3.2.0]heptane Libraries for Fragment-Based Drug Discovery

Mathilde A. C. H. Janssen,[a] Jesper W. A. van der Vorm,[a] Rico Rappard,[a] Bart Bijleveld,[a] Tom Dekker,[b] Maikel Wijtmans,[b] Iwan J. P. de Esch,[b] Daniel Blanco-Ania,[a] Floris P. J. T. Rutjes*[a]

[a] Institute for Molecules and Materials, Radboud University, Heyendaalseweg 135, 6525 AJ Nijmegen, the Netherlands email: floris.rutjes@ru.nl
[b] Amsterdam Institute of Molecular and Life Sciences, Vrije Universiteit Amsterdam, De Boelelaan 1108, 1081 HZ Amsterdam, the Netherlands

Supporting information

Contents **Page**

1) Experimental Procedures 2
2) Optimization for the Formation of Sulfonamides 14
3) ^{1}H and ^{13}C NMR Spectra 15
4) X-Ray Crystal Structure Data 56
5) References 57

1) Experimental Procedures

General Methods

The high-pressure experiments were run in a high-pressure apparatus equipped with a one-wall-piston cylinder for pressures up to 15 kbar (1.5 GPa). The high-pressure reactions were performed in 1–15 mL PTFE ampules closed by screwed stainless steel stoppers. These ampules were inserted into the high-pressure vessel filled with 100–140 petroleum ether as transmission medium. Reactions were followed, and R_F values were obtained, using thin-layer chromatography (TLC) on silica gel-coated plates (Merck 60 F254) with the indicated solvent mixture. Detection was performed with UV light, and/or by charring at ca. 150 °C after dipping into a solution of $KMnO_4$. ^{1}H and ^{13}C NMR spectra were recorded at 25 °C on a Bruker Avance III 500 MHz spectrometer with a Prodigy probe or a JEOL ECZ500R spectrometer with a SuperCOOL probe or on a Bruker Avance III HD nanobay 400 MHz using a BBFO probe. Peak assignment in ^{1}H and ^{13}C spectra is based on 2D COSY, HSQC, HMBC, and NOESY spectra when needed. The chemical shifts are reported in parts per million (ppm) relative to tetramethylsilane (TMS). Obtained data are presented in the following order: chemical shift, multiplicity (s = singlet, d = doublet, t = triplet, dd = doublet of doublets, ddd = doublet of doublet of doublets, ddt = doublet of doublet of triplets, tt = triplet of triplets, q = quartet, p = quintet, m = multiplet and/or multiple resonances), coupling constants *J* in Hertz (Hz), and integration. Purifications by column chromatography were carried out on a Biotage Isolera Spectra using silicycle cartridges (Biotage, 30–100 µm, 60 Å) 4–120 g. Preparative HPLC was performed on a Shimadzu LC-40 Nexera system equipped with a Kinetex-C18 column (150 × 21.20 mm, particle size 10 µm). High-resolution mass spectra (HRMS) were recorded on a JEOL AccuTOF JMS−T100CS (ESI).

Deposition Numbers 2380113 (for **3**) contain the supplementary crystallographic data for this paper. These data are provided free of charge by the joint Cambridge Crystallographic Data Centre and Fachinformationszentrum Karlsruhe Access Structures service.

Procedures

***rac*-(1*S*,2*R*)-2-*tert*-Butoxycyclobutane-1-sulfonyl fluoride (5a) and *rac*-(1*R*,2*R*)-2-*tert*-butoxycyclobutane-1-sulfonyl fluoride (5b)[1]**

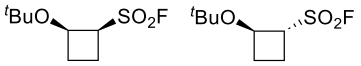

In a PTFE tube, ethenesulfonyl fluoride (**6**; 0.58 g, 0.44 mL, 5.3 mmol, 1.0 equiv) was added to a suspension of 2-methyl-2-(vinyloxy)propane (1.38 g, 1.79 mL, 13.8 mmol, 2.6 equiv) and $NaHCO_3$ (44 mg, 0.52 mmol, 0.1 equiv) in MeCN (10 mL). The tube was filled to 15 mL with MeCN, closed, and brought to 12 kbar at 21 °C for 15 min. The solvent was then removed *in vacuo* and the product was purified using column chromatography (heptane → 10:3 heptane/AcOEt) to afford a 1:1 mixture of cyclobutanes **5a** and **5b** (1.01 g, 4.83 mmol, 91%) as a slowly crystallizing oil. ***R*F**: 0.77 (heptane/AcOEt, 1:1). **FT-IR** (cm^{-1}, neat, ATR) ṽ = 2974, 1747, 1391, 1190, 1122, 1080, 743.

(1*S*,2*R*)-Isomer ^{1}H NMR [400 MHz, δ (ppm), $CDCl_3$]: 4.59–4.53 (m, 1 H,), 4.01–3.91 (m, 1 H), 2.40–2.28 (m, 1 H), 2.26–2.04 (m, 3 H), 1.20 (s, 9 H). **^{13}C NMR** [126 MHz, δ (ppm), $CDCl_3$]: 75.7, 65.6, 64.5 (d, *J* = 9.6 Hz,), 31.9, 27.6, 17.0. **^{19}F NMR** [377 MHz, δ (ppm), $CDCl_3$]: 57.3 (d, *J* = 3.0 Hz).

(1*R*,2*R*)-Isomer ^{1}H NMR [400 MHz, δ (ppm), $CDCl_3$]: 4.54–4.48 (m, 1 H), 4.17 (td, *J* = 7.4, 3.5 Hz, 1 H), 2.76–2.62 (m, 1 H), 2.40–2.28 (m, 2 H), 2.26–2.04 (m, 1 H), 1.22 (s, 9 H). **^{13}C NMR** [126 MHz, δ (ppm), $CDCl_3$]: 75.3, 67.4, 61.6 (d, *J* = 12.0 Hz), 30.4 (d, *J* = 2.4 Hz), 28.0, 16.3. **^{19}F NMR** [377 MHz, δ (ppm), $CDCl_3$]: 48.9 (d, *J* = 3.0 Hz).

***rac*-(1*S*,2*R*)-2-Hydroxycyclobutane-1-sulfonyl fluoride (7a) and *rac*-(1*R*,2*R*)-2-hydroxycyclobutane-1-sulfonyl fluoride (7b) [1]**

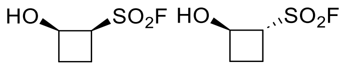

A 1 M solution of BBr_3 (in CH_2Cl_2, 44 mL, 44 mmol, 1.3 equiv) was added dropwise over 15 min to a cooled (0 °C) solution of cyclobutanesulfonyl fluorides **5a,b** (7.18 g, 34.1 mmol, 1.0 equiv) in anhydrous CH_2Cl_2. The reaction mixture was stirred at 21 °C for 2.5 h. The mixture was quenched with MeOH (20 mL) and the solvent was removed *in vacuo* to afford a 1:1 mixture of cyclobutanols **7a** and **7b** as a black oily solid that was used without further purification in the next step. ***R*F**: 0.50 (heptane/AcOEt, 1:4). **HRMS** [ESI (*m*/*z*)] calcd. for $(C_4H_7FO_3S + H)^+$ = 155.0173 found: 155.0170. **FT-IR** (cm^{-1}, neat, ATR) ṽ = 3359, 1390, 1193, 1120, 777.

(1*S*,2*R*)-Isomer ^{1}**H NMR** [400 MHz, δ (ppm), $CDCl_3$]: 4.82–4.74 (m, 1 H), 4.25 (dddd, *J* = 8.5, 7.2, 3.6, 1.5 Hz, 1 H), 2.58–2.50 (m, 2 H), 2.48–2.39 (m, 1 H), 2.35–2.17 (m, 1 H). ^{13}**C NMR** [126 MHz, δ (ppm), $CDCl_3$]: 66.3, 61.9 (d, *J* = 9.9 Hz), 31.4, 16.5. ^{19}**F NMR** [377 MHz, δ (ppm), $CDCl_3$]: 56.8.

(1*R*,2*R*)-Isomer ^{1}**H NMR** [400 MHz, δ (ppm), $CDCl_3$]: 4.74–4.65 (m, 1 H), 4.07–3.89 (m, 1 H), 2.48–2.39 (m, 1 H), 2.35–2.17 (m, 1 H), 2.15–2.01 (m, 2 H). ^{13}**C NMR** [126 MHz, δ (ppm), $CDCl_3$]: 67.8, 62.2 (d, *J* = 12.8 Hz), 28.5, 15.7. ^{19}**F NMR** [377 MHz, δ (ppm), $CDCl_3$]: 47.9.

rac-(1*R*,2*S*)-2-(Fluorosulfonyl)cyclobutyl trifluoromethanesulfonate (8a) and *rac*-(1*R*,2*R*)- 2-(fluorosulfonyl)cyclobutyl trifluoromethanesulfonate (8b) [1]

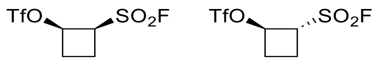

Trifluoromethanesulfonic anhydride (15.4 mL, 491.6 mmol, 2.5 equiv) was added to a cooled (0 °C) solution of cyclobutanols **7a,b** (5.632 g, 36.6 mmol, 1.0 equiv) and pyridine (7.4 mL, 91.8 mmol, 2.5 equiv) in anhydrous CH_2Cl_2. The reaction mixture was stirred at 21 °C for 2 h and then filtered over diatomaceous earth. The solvent was removed *in vacuo* to afford a 1:1 mixture of triflates **8a** and **8b** (5.3979 g, 18.9 mmol, 55% yield over two steps) as a brown oil after column chromatography (heptane → 3:2 heptane/AcOEt). **FT-IR** (cm^{-1}, neat, ATR) ṽ = 1752, 1410, 1200, 1137, 1086, 930, 614.

(1*R*,2*S*)-Isomer ^{1}**H NMR** [500 MHz, δ (ppm), $CDCl_3$]: 5.53–5.49 (m, 1 H), 4.48–4.43 (m, 1 H), 3.13–3.03 (m, 1 H), 2.76–2.72 (m, 1 H), 2.69–2.60 (m, 1 H), 2.45–2.36 (m, 1 H). ^{13}**C NMR*** [126 MHz, δ (ppm), $CDCl_3$]: 74.3, 60.4 (d, *J* = 18.4 Hz), 29.9, 17.3. ^{19}**F NMR** [377 MHz, δ (ppm), $CDCl_3$]: −74.8 (d, *J* = 2.3 Hz), 55.2.

(1*R*,2*R*)-Isomer ^{1}**H NMR** [500 MHz, δ (ppm), $CDCl_3$]: 5.58–5.52 (m, 1 H), 4.44–4.35 (m, 1 H), 2.72–2.68 (m, 1 H), 2.60–2.49 (m, 2 H), 2.36–2.27 (m, 1 H). ^{13}**C NMR*** [126 MHz, δ (ppm), $CDCl_3$]: 76.3, 59.0 (d, *J* = 18.9 Hz), 27.4, 16.2. ^{19}**F NMR** [377 MHz, δ (ppm), $CDCl_3$]: −75.0, 49.4.

*Not all carbon peaks are observed.

Cyclobut-1-ene-1-sulfonyl fluoride (4)

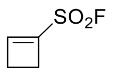

KO*t*Bu (19 mL of a 1 M solution in THF, 19 mmol, 1.1 equiv) was added dropwise to a cooled (0 °C) solution of triflates **8a,b** (4.90 g, 17.1 mmol, 1 equiv) in anhydrous CH_2Cl_2.The reaction mixture was stirred at 21 °C for 3 h and then filtered over diatomaceous earth. The reaction mixture was washed with water (140 mL). The organic layer was dried over Na_2SO_4, filtered, and carefully concentrated at 40 °C and 700 mbar to afford cyclobutene **4** (0.6853 g in THF, 5.0 mmol, 29%) as a yellow liquid.

^{1}**H NMR** [400 MHz, δ (ppm), $CDCl_3$]: 7.10 (dt, *J* = 2.4, 1.2 Hz, 1 H), 3.07 (td, *J* = 3.2, 1.2 Hz, 2 H), 2.79–2.71 (m, 2 H). ^{13}**C NMR** [126 MHz, δ (ppm), $CDCl_3$]: 152.2 (d, *J* = 4.2 Hz), 135.0 (d, *J* = 28.0 Hz), 31.3, 28.3. ^{19}**F NMR** [377 MHz, δ (ppm), $CDCl_3$]: 54.3. ***R*$_F$**: 0.48 (heptane/AcOEt, 2:3). **GC-HRMS** [EI (*m*/*z*)]: [$M^{+\cdot}$] calcd. for $C_4H_5FO_2S$: 135.9994 found: 136.0000.

rac-(1*R*,5*S*)-3-Benzyl-3-azabicyclo[3.2.0]heptane-1-sulfonyl fluoride (3) [2]

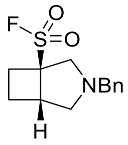

Trifluoroacetic acid (21.5 µl, 0.28 mmol, 0.15 equiv) in AcOEt (0.25 mL) was added to a cooled (0 °C) solution of cyclobut-1-ene-1-sulfonyl fluoride (**4**) (249.3 mg, 1.83 mmol, 1.0 equiv) and *N*-(methoxymethyl)-*N*-[(trimethylsilyl)methyl]benzylamine (**10**) (0.62 mL, 2.4 mmol, 1.3 equiv) in anhydrous AcOEt (3 mL). The reaction mixture was stirred at 21 °C for 4 h. The mixture was diluted with AcOEt (10 mL) and washed with a sat. aq. K_2CO_3 solution (10 mL), water (10 mL), and brine (10 mL). The organic layer was dried over Na_2SO_4, filtered and the solvent was removed *in vacuo* to afford bicyclosulfonyl fluoride **3** (368 mg, 1.37 mmol, 75%) as a white solid after purified by column chromatography (dry loaded on basic Al_2O_3, heptane → 4:1 heptane/AcOEt).

^{1}**H NMR** [400 MHz, δ (ppm), $CDCl_3$]: 7.40–7.26 (m, 5 H), 3.78 (d, *J* = 13.4, 1 H), 3.72 (d, *J* = 13.4, 1 H), 3.37–3.27 (m, 1 H), 3.15 (d, *J* = 9.5 Hz, 1 H), 2.93 (dd, *J* = 9.8, 1.9 Hz, 1 H), 2.88–2.74 (m, 1 H), 2.69 (d, *J* = 9.6 Hz, 1 H), 2.42 (dd, *J* = 9.7, 5.7 Hz, 1 H), 2.39–2.28 (m, 2 H), 1.98–1.85 (m, 1 H). ^{13}**C NMR** [101 MHz, δ (ppm), $CDCl_3$]: 138.4, 128.51 (2 C), 128.48 (2 C), 66.6, 59.2, 59.1, 58.9, 41.7, 26.5, 21.2. ^{19}**F NMR** [377 MHz, δ (ppm), $CDCl_3$]: 40.2 (SO_2*F*). ***R*$_F$**: 0.53 (heptane/AcOEt, 3:2). **FT-IR** (cm^{-1}, neat, ATR) ṽ = 1387, 1201, 1146, 747, 701. **HRMS** [ESI (*m*/*z*)] calcd. for $(C_{13}H_{16}FNO_2S + Na)^+$ = 292.0778 found: 292.0787.

General procedure A[3]

In a PTFE tube, the indicated amine (1.1–2.0 equiv) was added to a solution of $Ca(NTf_2)_2$ (1.1 equiv), DABCO (1.5 equiv), and bicyclosulfonyl fluoride **3** (1.0 equiv) in anhydrous THF (0.5 mL). The tube was filled to 1.5 mL with anhydrous THF, closed, and brought to 15 kbar at 21 °C for 16 h. The reaction mixture was diluted with AcOEt and washed with a sat. aq. $NaHCO_3$ solution (5 mL), water (5 mL), and brine (5 mL). The organic layer was dried over Na_2SO_4 and filtered. The solvent was removed *in vacuo* and the product was purified by column chromatography using the indicated eluent (dry loaded on basic Al_2O_3) to afford sulfonamides **13a**–**h**.

***rac*-(1*R*,5*S*)-3-Benzyl-*N*-methyl-3-azabicyclo[3.2.0]heptane-1-sulfonamide (13a)**

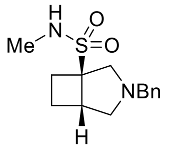

According to general procedure A, the reaction of bicyclosulfonyl fluoride **3** (65.0 mg, 0.24 mmol, 1.0 equiv), $Ca(NTf_2)_2$ (159.2 mg, 0.27 mmol, 1.1 equiv), DABCO (40.6 mg, 0.36 mmol, 1.5 equiv), and methanamine (0.05 mL of 9.8 M solution in MeOH, 0.49 mmol, 2.0 equiv) in anhydrous THF (1.3 mL) for 17 h afforded sulfonamide **13a** (44.5 mg, 159 µmol, 66%) as a colorless oil after column chromatography (heptane → 1:1 heptane/AcOEt).

^{1}H NMR [400 MHz, δ (ppm), $CDCl_3$]: 7.41–7.30 (m, 4 H), 7.29–7.25 (m, 1 H), 4.06 (q, *J* = 5.3 Hz, 1 H), 3.81–3.64 (m, 2 H), 3.18–3.11 (m, 1 H), 3.01 (d, *J* = 9.2 Hz, 1 H), 2.86 (d, *J* = 9.5 Hz, 1 H), 2.81 (d, *J* = 5.3 Hz, 3 H), 2.75–2.64 (m, 1 H), 2.61 (d, *J* = 9.1 Hz, 1 H), 2.40 (dd, *J* = 9.5, 5.7 Hz, 1 H), 2.29–2.14 (m, 2 H), 1.89–1.78 (m, 1 H). **^{13}C NMR** [101 MHz, δ (ppm), $CDCl_3$]: 139.0, 128.5 (2 C), 128.4 (2 C), 127.1, 68.5, 60.1, 59.8, 59.3, 40.9, 30.2, 26.8, 21.3. ***R*$_F$**: 0.21 (heptane/AcOEt, 3:2). **FT-IR** (cm^{-1}, neat, ATR) $\tilde{\nu}$ = 3288, 1301, 1112, 1036, 751, 694. **HRMS** [ESI (*m*/*z*)] calcd. for $(C_{14}H_{20}N_2O_2S + Na)^+$ = 303.1138 found: 303.1136.

***rac*-(1*R*,5*S*)-3-Benzyl-*N*-cyclopropyl-3-azabicyclo[3.2.0]heptane-1-sulfonamide (13b)**

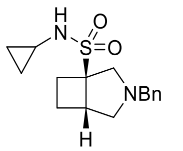

According to general procedure A, the reaction of bicyclosulfonyl fluoride **3** (74.8 mg, 0.278 mmol, 1.0 equiv), $Ca(NTf_2)_2$ (184.0 mg, 0.307 mmol, 1.1 equiv), DABCO (47.0 mg, 0.419 mmol, 1.5 equiv), and cyclopropanamine (21.2 µL, 0.306 mmol, 1.1 equiv) in anhydrous THF (1.3 mL) for 17 h afforded sulfonamide **13b** (61.5 mg, 201 µmol, 72%) as a colorless oil after column chromatography (heptane → 3:2 heptane/AcOEt). **^{1}H NMR** [400 MHz, δ (ppm), $CDCl_3$]: 7.43–7.33 (m, 4 H), 7.32–7.29 (m, 1 H), 4.52 (s, 1 H), 3.82–3.69 (m, 2 H), 3.29–3.22 (m, 1 H), 3.05 (d, *J* = 9.2 Hz, 1 H), 2.89 (d, *J* = 9.5 Hz, 1 H), 2.86–2.75 (m, 1 H), 2.68 (d, *J* = 9.2 Hz, 1 H), 2.59–2.52 (m, 1 H), 2.45 (dd, *J* = 9.5, 5.8 Hz, 1 H), 2.34–2.17 (m, 2 H), 1.94–1.81 (m, 1 H), 0.75–0.61 (m, 4H). **^{13}C NMR** [101 MHz, δ (ppm), $CDCl_3$]: 139.0, 128.5 (2 C), 128.4 (2 C), 127.1, 68.0, 60.3, 59.8, 59.3, 40.8, 26.8, 24.7, 21.4, 7.5, 7.2. ***R*$_F$**: 0.22 (heptane/AcOEt, 3:2). **HRMS** [ESI (*m*/*z*)] calcd. for $(C_{16}H_{22}N_2O_2S + Na)^+$ = 329.1294 found: 329.1294.

***rac*-(1*R*,5*S*)-3-Benzyl-*N*-cyclobutyl-3-azabicyclo[3.2.0]heptane-1-sulfonamide (13c)**

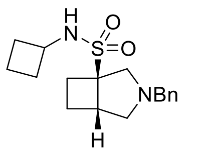

According to general procedure A, the reaction of bicyclosulfonyl fluoride **3** (74.9 mg, 0.278 mmol, 1.0 equiv), $Ca(NTf_2)_2$ (184.1 mg, 0.307 mmol, 1.1 equiv), DABCO (46.9 mg, 0.418 mmol, 1.5 equiv), and cyclobutanamine (26.2 µL, 0.307 mmol, 1.1 equiv) in anhydrous THF (1.3 mL) for 17 h afforded sulfonamide **13c** (52.9 mg, 165 µmol, 59%) as a colorless oil after column chromatography (heptane → 3:2 heptane/AcOEt).

^{1}H NMR [400 MHz, δ (ppm), $CDCl_3$]: 7.40–7.30 (m, 4 H), 7.29–7.23 (m, 1 H) 4.28 (d, *J* = 9.2 Hz, 1 H), 3.95 (qtd, *J* = 9.1, 7.6, 1.0 Hz, 1 H), 3.78–3.66 (m, 2 H), 3.07 (dt, *J* = 8.6, 5.5 Hz, 1 H), 2.97 (d, *J* = 9.1 Hz, 1 H), 2.85 (d, *J* = 9.4 Hz, 1 H), 2.68–2.60 (m, 1 H), 2.58 (d, *J* = 9.4 Hz, 1 H), 2.37 (dd, *J* = 9.5, 5.7 Hz, 1 H), 2.35–2.27 (m, 2 H), 2.25–2.11 (m, 2 H), 1.95–1.74 (m, 3 H), 1.69–1.59 (m, 2 H). **^{13}C NMR** [101 MHz, δ (ppm), $CDCl_3$]: 138.9, 128.5 (2 C), 128.3 (2 C), 127.1, 68.3, 60.1, 59.8, 59.3, 48.9, 40.7, 33.3, 33.0, 26.6, 21.0, 14.2. ***R*$_F$**: 0.30 (heptane/AcOEt, 3:2). **HRMS** [ESI (*m*/*z*)] calcd. for $(C_{17}H_{24}N_2O_2S + H)^+$ = 321.1631 found: 321.1635.

***rac*-(1*R*,5*S*)-3-Benzyl-*N*-phenyl-3-azabicyclo[3.2.0]heptane-1-sulfonamide (13d)**

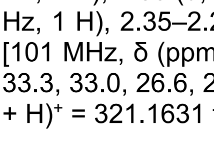

According to general procedure A, the reaction of bicyclosulfonyl fluoride **3** (50.1 mg, 0.186 mmol, 1.0 equiv), $Ca(NTf_2)_2$ (122.1 mg, 0.203 mmol, 1.1 equiv), DABCO (31.5 mg, 0.281 mmol, 1.5 equiv), and aniline (20 µL, 0.22 mmol, 1.2 equiv) in anhydrous THF (1.3 mL) for 16 h afforded sulfonamide **13d** (7.30 mg, 21.3 µmol, 11%) as a colorless oil after column chromatography (heptane → 3:2 heptane/AcOEt).

^{1}H NMR [400 MHz, δ (ppm), $CDCl_3$]: 7.35–7.27 (m, 4 H), 7.24–7.11 (m, 4 H), 6.80–6.65 (m, 2 H), 6.47 (s, 1 H), 3.65 (s, 2 H), 3.14–3.06 (m, 1 H), 2.84 (d, *J* = 9.3 Hz, 1 H), 2.74 (d, *J* = 9.5 Hz, 1 H), 2.71–2.62

(m, 1 H), 2.59 (d, *J* = 9.3 Hz, 1 H), 2.23 (dd, *J* = 9.5, 5.7 Hz, 1 H), 2.20–2.05 (m, 2 H), 1.81–1.71 (m, 1 H). **^{13}C NMR** [101 MHz, δ (ppm), $CDCl_3$]: 138.5, 136.8, 129.4 (2 C), 128.6 (2 C), 128.3 (2 C), 122.0 (2 C), 118.6, 115.1, 69.4, 60.0, 59.2, 59.1, 41.3, 26.9, 21.3. ***R*F**: 0.36 (heptane/AcOEt, 3:2). **FT-IR** (cm^{-1}, neat, ATR) ṽ = 3251, 1495, 1320, 1143, 916, 750, 695. **HRMS** [ESI (*m*/*z*)] calcd. for $(C_{19}H_{22}N_2O_2S + Na)^+$ = 365.1294 found: 365.1280.

***rac*-(1*R*,5*S*)-1-(Azetidine-1-sulfonyl)-3-benzyl-3-azabicyclo[3.2.0]heptane (13e)**

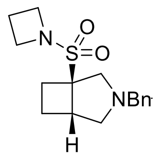

According to general procedure A, the reaction of bicyclosulfonyl fluoride **3** (50.2 mg, 0.184 mmol, 1.0 equiv), $Ca(NTf_2)_2$ (122.1 mg, 0.203 mmol, 1.1 equiv), DABCO (31.5 mg, 0.281 mmol, 1.5 equiv), and azetidine hydrochloride (19.1 mg, 0.204 mmol, 1.1 equiv) in anhydrous THF (1.3 mL) for 16 h afforded sulfonamide **13e** (7.40 mg, 24.1 µmol, 13%) as a colorless oil after column chromatography (heptane → 3:2 heptane/AcOEt).

^{1}H NMR [400 MHz, δ (ppm), $CDCl_3$]: 7.42–7.26 (m, 5 H), 3.96 (t, *J* = 7.7 Hz, 4 H), 3.79–3.65 (m, 2 H), 3.12–3.04 (m, 1 H), 2.98 (d, *J* = 9.2 Hz, 1 H), 2.85 (d, *J* = 9.4 Hz, 1 H), 2.71–2.58 (m, 1 H), 2.55 (d, *J* = 9.2 Hz, 1 H), 2.36 (dd, *J* = 9.5, 5.7 Hz, 1 H), 2.27–2.21 (m, 2 H), 2.22–2.09 (m, 2 H), 1.87–1.75 (m, 1 H). **^{13}C NMR** [101 MHz, δ (ppm), $CDCl_3$]: 139.1, 128.5 (2 C), 128.3 (2 C), 127.0, 67.8, 59.8, 59.7, 59.3, 51.1, 40.4, 26.3, 21.2, 15.6. ***R*F**: 0.35 (heptane/AcOEt, 3:2). **FT-IR** (cm^{-1}, neat, ATR) ṽ = 1311, 1143, 945, 698, 643. **HRMS** [ESI (*m*/*z*)] calcd. for $(C_{16}H_{22}N_2O_2S + H)^+$ = 307.1475 found: 307.1472.

***rac*-(1*R*,5*S*)-3-Benzyl-1-(pyrrolidine-1-sulfonyl)-3-azabicyclo[3.2.0]heptane (13f)**

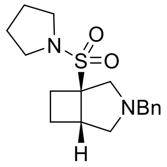

According to general procedure A, the reaction of bicyclosulfonyl fluoride **3** (43.7 mg, 0.16 mmol, 1.0 equiv), $Ca(NTf_2)_2$ (108.4 mg, 0.18 mmol, 1.1 equiv), DABCO (27.5 mg, 0.25 mmol, 1.5 equiv), and pyrrolidine (14.5 µL, 0.17 mmol, 1.1 equiv) in anhydrous THF (1.3 mL) for 16 h afforded sulfonamide **13f** (5.70 mg, 17.8 µmol, 11%) as an orange solid after column chromatography (heptane → 1:1 heptane/AcOEt).

^{1}H NMR [400 MHz, δ (ppm), $CDCl_3$]: 7.41–7.25 (m, 5 H), 3.79–3.66 (m, 2 H), 3.43–3.31 (m, 4 H), 3.22–3.15 (m, 1 H), 2.99 (d, *J* = 9.1 Hz, 1 H), 2.85 (d, *J* = 9.5 Hz, 1 H), 2.80–2.67 (m, 1 H), 2.63 (d, *J* = 9.1 Hz, 1 H), 2.37 (dd, *J* = 9.5, 5.7 Hz, 1 H), 2.30–2.11 (m, 2 H), 1.90–1.84 (m, 4 H), 1.85–1.77 (m, 1 H). **^{13}C NMR** [101 MHz, δ (ppm), $CDCl_3$]: 139.0, 128.5 (2 C), 128.3 (2 C), 127.0, 69.2, 60.0, 59.7, 59.3, 48.6, 41.0, 26.9, 26.0, 21.4. ***R*F**: 0.29 (heptane/AcOEt, 3:2). **FT-IR** (cm^{-1}, neat, ATR) ṽ = 1317, 1143, 1127, 747, 698, 627. **HRMS** [ESI (*m*/*z*)] calcd. for $(C_{17}H_{24}N_2O_2S + Na)^+$ = 343.1451 found: 343.1446.

***rac*-(1*R*,5*S*)-3-Benzyl-*N*,*N*-diethyl-3-azabicyclo[3.2.0]heptane-1-sulfonamide (13g)**

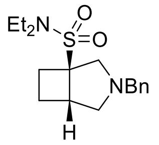

According to general procedure A, the reaction of bicyclosulfonyl fluoride **3** (50.3 mg, 0.187 mmol, 1.0 equiv), $Ca(NTf_2)_2$ (122.5 mg, 0.204 mmol, 1.1 equiv), DABCO (32.0 mg, 0.285 mmol, 1.5 equiv), and diethylamine (15.2 mg, 21.5 µL, 208 µmol, 1.1 equiv) in anhydrous THF (1.3 mL) for 16 h afforded sulfonamide **13g** (3.90 mg, 12.1 µmol, 6%) as a colorless oil after column chromatography (heptane → 7:3 heptane/AcOEt).

^{1}H NMR [400 MHz, δ (ppm), $CDCl_3$]: 7.44–7.27 (m, 5 H), 3.72 (s, 2 H), 3.41–3.19 (m, 4 H), 3.20–3.12 (m, 1 H), 2.96 (d, *J* = 9.0 Hz, 1 H), 2.83 (d, *J* = 9.4 Hz, 1 H), 2.74–2.64 (m, 1 H), 2.60 (d, *J* = 9.0 Hz, 1 H), 2.36 (dd, *J* = 9.4, 5.6 Hz, 1 H), 2.28–2.11 (m, 2 H), 1.85–1.74 (m, 1 H), 1.14 (t, *J* = 7.1 Hz, 6 H). **^{13}C NMR** [101 MHz, δ (ppm), $CDCl_3$]: 139.2, 128.5 (2 C), 128.3 (2 C), 127.0, 69.4, 60.2, 59.7, 59.3, 41.40, 41.37, 27.1, 21.3, 14.3. ***R*F**: 0.37 (heptane/AcOEt, 3:2). **FT-IR** (cm^{-1}, neat, ATR) ṽ = 2923, 1313, 1019, 757, 698. **HRMS** [ESI (*m*/*z*)] calcd. for $(C_{19}H_{22}N_2O_2S + Na)^+$ = 345.1607 found: 345.1608.

General procedure B[4]

1-Chloroethyl chloroformate (1.3 equiv) was added to a solution of bicyclosulfonyl fluoride **3** or sulfonamides **13a**–**c** in anhydrous CH_2Cl_2. The reaction mixture was stirred at 40 °C for 18 h after which the solvent was removed *in vacuo*. The residue was dissolved in MeOH and the mixture was heated to 65 °C for 2 h. The solvent was removed *in vacuo* and the product was purified by trituration with the indicated eluent to afford secondary amines **12** and **14a**–**c**.

rac-(1_R_,5_S_)-3-Azabicyclo[3.2.0]heptane-1-sulfonyl fluoride hydrochloride (12)

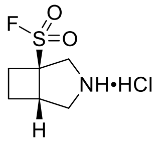

According to general procedure B, the reaction was conducted with bicyclosulfonyl fluoride **3** (208 mg, 0.78 mmol, 1.0 equiv) and 1-chloroethyl chloroformate (110 µL, 1.0 mmol, 1.3 equiv) in anhydrous CH_2Cl_2 (5.0 mL). The reaction mixture was stirred at 40 °C for 18 h. The solvent was removed *in vacuo* and the residue was redissolved in MeOH (5.0 mL). The resulting mixture was stirred at 65 °C for 2 h to afford the secondary amine hydrochloride **12** (105.2 mg, 0.49 mmol, 63%) as a light brown solid after column chromatography (CH_2Cl_2 → 4:1 CH_2Cl_2/MeOH).

^{1}H NMR [400 MHz, δ (ppm), CD_3OD]: 4.06 (d, *J* = 13.2 Hz, 1 H), 3.89–3.84 (m, 1 H), 3.84–3.76 (m, 1 H), 3.64 (d, *J* = 12.5 Hz, 1 H), 3.49 (dd, *J* = 12.5, 6.6 Hz, 1 H), 3.06–2.93 (m, 1 H), 2.61–2.40 (m, 2 H), 2.10–1.96 (m, 1 H). **^{13}C NMR** [101 MHz, δ (ppm), CD_3OD]: 66.5 (S*C*), 51.2, 50.9, 41.6, 24.9, 19.1. **^{19}F NMR** [377 MHz, δ (ppm), CD_3OD]: 39.8. ***R***$_F$: 0.20 (CH_2Cl_2/MeOH, 9:1). **FT-IR** (cm^{-1}, neat, ATR) ṽ = 2668, 1566, 1387, 1203, 1166, 788, 765, 613. **HRMS** [ESI (*m*/*z*)] calcd. for $(C_6H_{10}FNO_2S + H)^+$ = 180.0489 found: 180.0491.

rac-(1_R_,5_S_)-_N_-Methyl-3-azabicyclo[3.2.0]heptane-1-sulfonamide hydrochloride (14a)

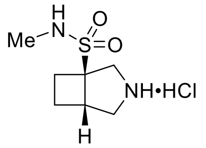

According to general procedure B, the reaction was conducted with sulfonamide **13a** (75.4 mg, 0.27 mmol, 1.0 equiv) and 1-chloroethyl chloroformate (37.7 µL, 0.35 mmol, 1.3 equiv) in anhydrous CH_2Cl_2 (1.0 mL). The reaction mixture was stirred at 40 °C for 18 h. The solvent was removed *in vacuo* and the residue was redissolved in MeOH (1.0 mL). The resulting mixture was stirred at 65 °C for 2 h to afford secondary amine hydrochloride **14a** (33.5 mg, 148 µmol, 55%) as a white solid after trituration with acetone.

^{1}H NMR [400 MHz, δ (ppm), CD_3OD]: 3.77 (d, *J* = 12.6 Hz, 1 H), 3.68 (dd, *J* = 12.6, 1.2 Hz, 1 H), 3.54–3.46 (m, 2 H), 3.40–3.33 (m, 1 H), 2.88–2.77 (m, 1 H), 2.81 (s, 3 H), 2.45–2.34 (m, 1 H), 2.30–2.20 (m, 1 H), 1.95–1.84 (m, 1 H). **^{13}C NMR** [101 MHz, δ (ppm), CD_3OD]: 67.8, 51.7, 51.4, 40.7, 28.6, 24.9, 19.1. **FT-IR** (cm^{-1}, neat, ATR) ṽ = 3145, 1559, 1401, 1251, 1143, 1087, 847, 613. **HRMS** [ESI (*m*/*z*)] calcd. for $(C_7H_{14}N_2O_2S + H)^+$ = 191.0849 found: 191.0842.

rac-(1_R_,5_S_)-_N_-Cyclopropyl-3-azabicyclo[3.2.0]heptane-1-sulfonamide hydrochloride (14b)

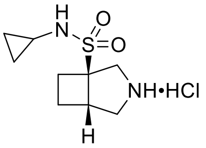

According to general procedure B, the reaction was conducted with sulfonamide **13b** (61.5 mg, 0.20 mmol, 1.0 equiv) and 1-chloroethyl chloroformate (28.2 µL, 0.26 mmol, 1.3 equiv) in anhydrous CH_2Cl_2 (1.0 mL). The reaction mixture was stirred at 40 °C for 18 h. The solvent was removed *in vacuo* and the residue was redissolved in MeOH (1.0 mL). The resulting mixture was stirred at 65 °C for 2 h to afford secondary amine hydrochloride **14b** (39.2 mg, 155 µmol, 77%) as a white solid after trituration with acetone.

^{1}H NMR [400 MHz, δ (ppm), CD_3OD]: 3.82 (d, *J* = 12.7 Hz, 1 H), 3.74 (dd, *J* = 12.6, 1.2 Hz, 1 H), 3.60–3.54 (m, 1 H), 3.55–3.49 (m, 1 H), 3.42–3.34 (m, 1 H), 2.91 (dddd, *J* = 13.2, 11.3, 7.6, 1.1 Hz, 1 H), 2.61–2.52 (m, 1 H), 2.47–2.35 (m, 1 H), 2.32–2.22 (m, 1 H), 1.97–1.85 (m, 1 H), 0.75–0.59 (m, 4 H). **^{13}C NMR** [101 MHz, δ (ppm), CD_3OD]: 67.3, 51.9, 51.4, 40.7, 24.9, 24.0, 19.2, 6.0, 5.8. **FT-IR** (cm^{-1}, neat, ATR) ṽ = 3147, 1600, 1415, 1320, 1143, 1010, 882, 624. **HRMS** [ESI (*m*/*z*)] calcd. for $(C_9H_{16}N_2O_2S + Na)^+$ = 239.0825 found: 239.0822.

rac-(1_R_,5_S_)-_N_-Cyclobutyl-3-azabicyclo[3.2.0]heptane-1-sulfonamide hydrochloride (14c)

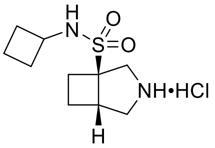

According to general procedure B, the reaction was conducted with sulfonamide **13c** (49.6 mg, 0.155 mmol, 1.0 equiv) and 1-chloroethyl chloroformate (22.0 µL, 0.204 mmol, 1.3 equiv) in anhydrous CH_2Cl_2 (1.0 mL). The reaction mixture was stirred at 40 °C for 18 h. The solvent was removed *in vacuo* and the residue was redissolved in MeOH (1.0 mL). The resulting mixture was stirred at 65 °C for 2 h to afford secondary amine hydrochloride **14c** (41.6 mg, 156 µmol, 100%) as a brown solid.

^{1}H NMR [400 MHz, δ (ppm), CD_3OD]: 3.96 (p, *J* = 8.4 Hz, 1 H), 3.73–3.61 (m, 2 H), 3.50 (dd, *J* = 12.1, 1.1 Hz, 1 H), 3.46–3.39 (m, 1 H), 3.36–3.34 (m, 1 H), 2.84–2.71 (m, 1 H), 2.41–2.25 (m, 3 H), 2.22–2.15 (m, 1 H), 2.08–1.96 (m, 2 H), 1.91–1.81 (m, 1 H), 1.72–1.57 (m, 2 H). **^{13}C NMR** [101 MHz, δ (ppm), CD_3OD]: 67.5 (S*C*), 51.8, 51.6, 48.7, 40.4, 32.2, 31.9, 24.7, 18.9, 13.5. **FT-IR** (cm^{-1}, neat, ATR) ṽ = 3351, 3120, 1605, 1448, 1315, 1307, 1136, 909, 617. **HRMS** [ESI (*m*/*z*)] calcd. for $(C_{10}H_{18}N_2O_2S + H)^+$ = 231.1162 found: 231.1164.

General procedure C[5]

The indicated acyl chloride (1.2–1.3 equiv) was added to a mixture of secondary amines **12** and **14a**–**c** and Et_3N (2.4–2.5 equiv) in CH_2Cl_2. The reaction mixture was stirred at 21–40 °C for 4–41 h. The solvent was removed *in vacuo* and the product was purified by column chromatography using the indicated eluent and prep HPLC to afford amides **1a**–**d** and **2aa**, **2bb**, and **2cc**.

***rac*-(1*R*,5*S*)-3-(Cyclopropanecarbonyl)-3-azabicyclo[3.2.0]heptane-1-sulfonyl fluoride (1a)**

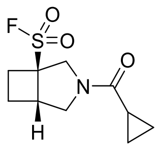

According to general procedure C, the reaction of secondary amine **12** (14.8 mg, 0.069 mmol, 1.0 equiv), Et_3N (24.2 µl, 0.17 mmol, 2.5 equiv), and cyclopropanecarbonyl chloride (8.2 µL, 0.09 mmol, 1.3 equiv) in anhydrous CH_2Cl_2 (0.25 mL) for 17 h afforded amide **1a** (14.1 mg, 57 µmol, 83%) as a colorless oil after column chromatography (dry loaded on silica, CH_2Cl_2 → 9:1 CH_2Cl_2/MeOH).

^{1}H NMR* [500 MHz, δ (ppm), $CDCl_3$]: 4.35–4.28 (m, 1 H), 4.07–3.97 (m, 1 H), 3.89 (d, *J* = 11.0 Hz, 1 H), 3.80–3.71 (m, 1 H), 3.66–6.58 (m, 1 H), 3.03–2.87 (m, 1 H), 2.54–2.39 (m, 1 H), 2.36–2.23 (m, 1 H), 2.01–1.85 (m, 1 H), 1.70–1.60 (m, 1 H), 1.12–1.04 (m, 2 H), 0.91–0.80 (m, 2 H). **^{13}C NMR*** [126 MHz, δ (ppm), $CDCl_3$]: 173.0, 52.2, 52.2, 42.6, 26.8, 21.1, 12.7, 8.2. **^{19}F NMR** [471 MHz, δ (ppm), $CDCl_3$]: 39.9. ***R*$_F$**: 0.72 (CH_2Cl_2/MeOH, 9:1). **FT-IR** (cm^{-1}, neat, ATR) ṽ = 1640, 1432, 1391, 1199, 758, 617. **HRMS** [ESI (*m*/*z*)] calcd. for $(C_{10}H_{14}FNO_3S + Na)^+$ = 270.0571 found: 270.0573.

*Most of the signals were broadened because of slow rotation of the amide bond and not all carbon peaks are observed.

***rac*-(1*R*,5*S*)-3-(Furan-2-carbonyl)-3-azabicyclo[3.2.0]heptane-1-sulfonyl fluoride (1b)**

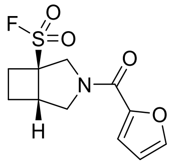

According to general procedure C, the reaction of secondary amine **12** (9.9 mg, 0.046 mmol, 1.0 equiv), Et_3N (16.2 µl, 0.12 mmol, 2.5 equiv), and 2-furoyl chloride (6 µL, 0.061 mmol, 1.3 equiv) in anhydrous CH_2Cl_2 (0.25 mL) for 22 h (additional 2-furoyl chloride (6 µl, 0.061 mmol, 1.3 equiv) was added after 20 h) afforded amide **1b** (9.3 mg, 34 µmol, 74%) as a white solid after column chromatography (dry loaded on silica, CH_2Cl_2 → 9:1 CH_2Cl_2/MeOH).

^{1}H NMR* [500 MHz, δ (ppm), $CDCl_3$]: 7.57–7.51 (m, 1 H), 7.21 (d, *J* = 3.5 Hz, 1 H), 6.55 (dd, *J* = 3.5, 1.8 Hz, 1 H), 4.66–4.50 (m, 2 H), 4.38–4.11 (m, 2 H), 3.72–3.49 (m, 1 H), 3.02–2.92 (m, 1 H), 2.52–2.41 (m, 1 H), 2.31 (ddd, *J* = 13.9, 9.5, 5.0 Hz, 1 H), 1.94 (dddd, *J* = 12.5, 9.6, 8.3, 6.4 Hz, 1 H). **^{13}C NMR*** [126 MHz, δ (ppm), $CDCl_3$]: 158.5, 147.9, 144.8, 117.8, 111.9, 53.2, 52.6, 26.8, 21.1. **^{19}F NMR** [471 MHz, δ (ppm), $CDCl_3$]: 40.3. ***R*$_F$**: 0.81 (CH_2Cl_2/MeOH, 9:1). **FT-IR** (cm^{-1}, neat, ATR) ṽ = 3108, 1603, 1486, 1388, 1177, 1016, 76, 738, 619. **HRMS** [ESI (*m*/*z*)] calcd. for $(C_{11}H_{12}FNO_4S + H)^+$ = 274.0544 found: 274.0534.

*Most of the signals were broadened because of slow rotation of the amide bond and not all carbon peaks are observed.

***rac*-(1*R*,5*S*)-3-(1-Methyl-1*H*-pyrazole-4-carbonyl)-3-azabicyclo[3.2.0]heptane-1-sulfonyl fluoride (1c)**

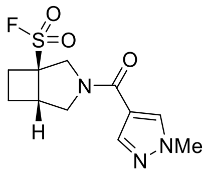

According to general procedure C, the reaction of secondary amine **12** (14.7 mg, 0.068 mmol, 1.0 equiv), Et_3N (24.0 µl, 0.17 mmol, 2.5 equiv), and 1-methylpyrazole-4-carbonyl chloride (13.1 mg, 0.091 mmol, 1.3 equiv) in anhydrous CH_2Cl_2 (0.25 mL) for 20 h and additionally at 40 °C for 2 h afforded amide **1c** (9.8 mg, 34 µmol, 50%) as a colorless oil after column chromatography (dry loaded on silica, CH_2Cl_2 → 4:1 CH_2Cl_2/MeOH) and prep HPLC.

^{1}H NMR* [500 MHz, δ (ppm), $CDCl_3$]: 7.82 (s, 1 H), 7.72 (s, 1 H), 4.66–4.30 (m, 2 H), 4.23–4.10 (m, 1 H), 3.93 (s, 3 H), 3.84–3.67 (m, 1 H), 3.65–3.53 (m, 1 H), 2.98–2.88 (m, 1 H), 2.48–2.38 (m, 1 H), 2.34–2.21 (m, 1 H), 2.01–1.79 (m, 1 H). **^{13}C NMR*** [126 MHz, δ (ppm), $CDCl_3$]: 163.1, 139.4, 133.1, 117.7, 39.4, 26.8, 21.0. **^{19}F NMR** [471 MHz, δ (ppm), $CDCl_3$]: 40.3. ***R*$_F$**: 0.37 (CH_2Cl_2/MeOH, 9:1). **FT-IR** (cm^{-1}, neat, ATR) ṽ = 1600, 1549, 1390, 1197, 751. **HRMS** [ESI (*m*/*z*)] calcd. for $(C_{11}H_{14}FN_3O_3S + H)^+$ = 288.0813 found: 288.0821.

*Some of the signals were broadened because of slow rotation of the amide bond and not all carbon peaks are observed.

***rac*-(1*R*,5*S*)-3-(1,3-Thiazole-4-carbonyl)-3-azabicyclo[3.2.0]heptane-1-sulfonyl fluoride (1d)**

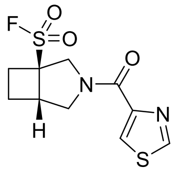

According to general procedure C, the reaction of secondary amine **12** (12.3 mg, 0.057 mmol, 1.0 equiv), Et_3N (20 µL, 0.14 mmol, 2.5 equiv) and 1,3-thiazole-4-carbonyl chloride (11 mg, 0.075 mmol, 1.3 equiv) in anhydrous CH_2Cl_2 (0.25 mL) for 20 h and additionally at 40 °C for 21 h afforded amide **1d** (4.2 mg, 14.4 µmol, 25%) as a white solid after column chromatography (dry loaded on silica, CH_2Cl_2 → 9:1 CH_2Cl_2/MeOH) and prep HPLC.

^{1}H NMR* [500 MHz, δ (ppm), $CDCl_3$]: 8.84–8.78 (m, 1 H), 8.29–8.23 (m, 1 H), 5.05 (d, *J* = 13.3 Hz, 1 H), 4.75 (d, *J* = 12.5 Hz, 1 H), 4.58 (d, *J* = 13.9 Hz, 1 H), 4.39 (d, *J* = 13.3 Hz, 1 H), 4.26 (d, *J* = 12.9 Hz, 1 H), 4.16 (d, *J* = 13.9 Hz, 1 H), 3.89–3.81 (m, 1 H), 3.71–3.67 (m, 1 H), 3.66–3.60 (m, 1 H), 3.57–3.25 (m, 1 H), 3.01–2.90 (m, 1 H), 2.53–2.39 (m, 1 H), 2.38–2.28 (m, 1 H), 2.02–1.90 (m, 1 H). **^{13}C NMR*** [126 MHz, δ (ppm), $CDCl_3$]: 162.0 (major), 161.7 (minor), 152.3 (minor), 152.2 (major), 151.35, 126.8 (minor), 126.7 (major), 67.6 (major), 64.7 (minor), 54.4 (major), 53.9 (minor), 53.0, 52.3, 43.2 (minor), 40.3 (major), 26.9 (minor), 26.5 (major), 21.3 (major), 20.8 (minor). **^{19}F NMR*** [471 MHz, δ (ppm), $CDCl_3$]: 40.5, 40.2. ***R*$_F$**: 0.77 (CH_2Cl_2/MeOH, 9:1). **FT-IR** (cm^{-1}, neat, ATR) ṽ = 3057, 1605, 1424, 1388, 1206, 1183, 868, 765, 743, 609.**HRMS** [ESI (*m*/*z*)] calcd. for $(C_{10}H_{11}FN_2O_3S_2 + Na)^+$ = 313.0087 found: 313.0084.

*Rotamers observed.

***rac*-(1*R*,5*S*)-3-Benzoyl-*N*-methyl-3-azabicyclo[3.2.0]heptane-1-sulfonamide (2aa)**

According to general procedure C, the reaction of secondary amine **14a** (10.5 mg, 0.046 mmol, 1.0 equiv), Et_3N (15.5 µL, 0.11 mmol, 2.4 equiv), and benzoyl chloride (6.2 µL, 0.053 mmol, 1.15 equiv) in anhydrous CH_2Cl_2 (0.25 mL) for 4 h afforded amide **2aa** (6.50 mg, 22.1 µmol, 48%) as a white solid after column chromatography (CH_2Cl_2 → 9:1 CH_2Cl_2/MeOH) and prep HPLC.

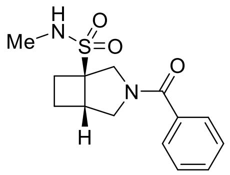

^{1}H NMR* [500 MHz, δ (ppm), $CDCl_3$]: 7.57–7.52 (m, 2 H), 7.51–7.42 (m, 3 H), 4.60–4.49 (m, 1 H), 4.29–4.18 (m, 1 H), 3.99 (d, *J* = 12.9 Hz, 1 H), 3.70–3.61 (m, 1 H), 3.60–3.53 (m, 1 H), 3.42–3.33 (m, 1 H), 2.92–2.82 (m, 4 H), 2.39–2.30 (m, 1 H), 2.19 (m, 1 H), 1.66–1.54 (m, 1 H). **^{13}C NMR*** [126 MHz, δ (ppm), $CDCl_3$]: 170.42, 136.1, 130.4 (2 C), 128.6, 127.3 (2 C), 67.4, 55.0, 52.6, 42.0, 30.2, 26.9, 20.7. ***R*$_F$**: 0.52 (CH_2Cl_2/MeOH, 9:1). **FT-IR** (cm^{-1}, neat, ATR) ṽ = 3241, 1608, 1573, 1449, 1305, 1150, 1096, 1079, 717. **HRMS** [ESI (*m*/*z*)] calcd. for $(C_{14}H_{18}N_2O_3S + H)^+$ = 295.1111 found: 295.1108.

*Most of the signals were broadened because of slow rotation of the amide bond.

***rac*-(1*R*,5*S*)-*N*-Cyclopropyl-3-(2-methoxyacetyl)-3-azabicyclo[3.2.0]heptane-1-sulfonamide (2bb)**

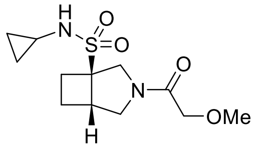

According to general procedure C, the reaction of secondary amine **14b** (10 mg, 0.04 mmol, 1.0 equiv), Et_3N (13.8 µL, 0.099 mmol, 2.5 equiv), and 2-methoxyacetyl chloride (4.5 µL, 0.049 mmol, 1.24 equiv) in anhydrous CH_2Cl_2 (0.25 mL) for 22 h afforded amide **2bb** (10.3 mg, 35.7 µmol, 90%) as a colorless liquid after column chromatography (CH_2Cl_2 → 19:1 CH_2Cl_2/MeOH) and prep HPLC.

^{1}H NMR* [400 MHz, δ (ppm), $CDCl_3$]: 4.66 (s, 1 H, major), 4.64 (s, 1 H, minor), 4.24–3.87 (m, 8 H), 3.67–3.48 (m, 5 H), 3.46 (s, 3 H, minor), 3.45 (s, 3 H, major), 3.44–3.40 (m, 1 H, minor), 2.96–2.84 (m, 2 H), 2.63–2.50 (m, 2 H), 2.41–2.29 (m, 2 H), 2.15–2.05 (m, 2 H), 1.85–1.73 (m, 2 H), 0.79–0.58 (m, 8 H). **^{13}C NMR*** [101 MHz, δ (ppm), $CDCl_3$]: 168.6 (major), 168.2 (minor), 72.6 (minor), 72.0 (major), 68.6 (minor), 66.1 (major), 59.20 (major), 59.15 (minor), 53.2 (major), 52.9 (minor), 51.9 (minor), 51.6 (major), 41.9 (major), 39.5 (minor), 27.1 (major), 26.7 (minor), 25.0 (minor), 24.6 (major), 21.3 (minor), 21.0 (major), 7.5 (2 C, major), 6.9 (2 C, minor). ***R*$_F$**: 0.47 (CH_2Cl_2/MeOH, 9:1). **FT-IR** (cm^{-1}, neat, ATR) ṽ = 3171, 1636, 1457, 1308, 1144, 1120, 888, 620. **HRMS** [ESI (*m*/*z*)] calcd. for $(C_{12}H_{20}N_2O_4S + Na)^+$ = 311.1036 found: 311.1033.

*Rotamers observed.

***rac*-(1*R*,5*S*)-3-Acetyl-*N*-cyclobutyl-3-azabicyclo[3.2.0]heptane-1-sulfonamide (2cc)**

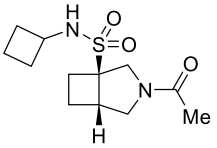

According to general procedure C, the reaction of secondary amine **14c** (8.2 mg, 0.030 mmol, 1.0 equiv), Et_3N (13.1 µL, 0.94 mmol, 2.5 equiv), and acetyl chloride (2.7 µL, 0.038 mmol, 1.2 equiv) in anhydrous CH_2Cl_2 (0.25 mL) for 17 h afforded amide **2cc** (4.1 mg, 15 µmol, 49%) as a white solid after column chromatography (CH_2Cl_2 → 19:1 CH_2Cl_2/MeOH).

***R*F**: 0.36 (CH_2Cl_2/MeOH, 9:1). **FT-IR** (cm^{-1}, neat, ATR) ṽ = 3120, 1628, 1454, 1311, 1140, 911, 554. **HRMS** [ESI (*m*/*z*)] calcd. for $(C_{12}H_{20}N_2O_3S + H)^+$ = 273.1267 found: 273.1268.

Major-rotamer ^{1}H NMR* [500 MHz, δ (ppm), $CDCl_3$]: 4.97 (d, *J* = 9.1 Hz, 1 H), 4.07 (d, *J* = 13.3 Hz, 1 H), 3.97–3.94 (m, 1 H), 3.92–3.88 (m, 1 H,), 3.64 (dd, *J* = 11.0, 6.2 Hz, 1 H), 3.53 (d, *J* = 11.0 Hz, 1 H), 3.44–3.38 (m, 1 H), 2.79 (td, *J* = 11.5, 8.2 Hz, 1 H), 2.41–2.36 (m, 2 H), 2.34–2.25 (m, 3 H), 2.13 (s, 3 H), 2.07 (ddd, *J* = 12.8, 9.5, 4.5 Hz, 1 H), 2.00–1.90 (m, 3 H), 1.83–1.74 (m, 1 H), 1.74–1.64 (m, 2 H). **^{13}C NMR*** [126 MHz, δ (ppm), $CDCl_3$]: 169.9, 67.1, 53.6, 52.9, 48.9, 41.6, 33.9, 32.7, 26.9, 22.6, 20.9, 14.3.

Minor-rotamer ^{1}H NMR* [500 MHz, δ (ppm), $CDCl_3$]: 4.73 (d, *J* = 9.1 Hz, 1 H), 4.04–3.97 (m, 1 H), 3.97–3.95 (m, 1 H), 3.94 (d, *J* = 6.6 Hz, 1 H), 3.80 (d, *J* = 11.7 Hz, 1 H), 3.40 (m, 1 H), 3.26 (q, *J* = 6.9 Hz, 1 H), 2.82–2.74 (m, 1 H), 2.34–2.31 (m, 1 H), 2.32–2.26 (m, 1 H), 2.16 (s, 3 H), 2.07 (ddd, *J* = 12.8, 9.5, 4.5 Hz, 1 H), 1.93–1.88 (m, 3 H), 1.84–1.77 (m, 1 H) 1.64–1.55 (m, 2 H). **^{13}C NMR*** [126 MHz, δ (ppm), $CDCl_3$]: 169.6, 68.9, 54.5, 51.7, 49.2, 40.2, 33.2, 33.0, 26.8, 22.8, 21.0, 14.2.

*Rotamers observed.

General procedure D[6]

The indicated isocyanate (1.2–1.4 equiv) was added to a mixture of secondary amine **12** and **14a**–**c** and Et_3N (2.5–3.6 equiv) in THF or CH_2Cl_2. The reaction mixture was stirred at 21–40 °C for 16–43 h. The solvent was removed *in vacuo* and the product was purified by column chromatography using the indicated eluent to afford ureas **1e**, **1f**, **2ad, 2be,** and **2cf**.

***rac*-(1*R*,5*S*)-3-(Ethylcarbamoyl)-3-azabicyclo[3.2.0]heptane-1-sulfonyl fluoride (1e)**

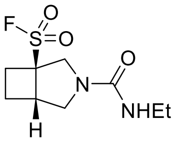

According to general procedure D, the reaction of secondary amine **12** (9.7 mg, 0.045 mmol, 1.0 equiv), Et_3N (16.2 µL, 0.12 mmol, 2.6 equiv), and ethyl isocyanate (4.4 µL, 0.056 mmol, 1.2 equiv) in anhydrous CH_2Cl_2 (0.25 mL) for 43 h (additional ethyl isocyanate (4.4 µl, 0.056 mmol, 1.2 equiv) was added after 18 h) afforded urea **1e** (9.5 mg, 22.1 µmol, 84%) as a white solid after column chromatography (dry loaded on silica, CH_2Cl_2 → 17:3 CH_2Cl_2/MeOH).

^{1}H NMR [400 MHz, δ (ppm), CD_3OD]: 6.55 (s, 1 H), 4.07 (d, *J* = 12.1 Hz, 1 H), 3.89 (dd, *J* = 12.1, 1.1 Hz, 1 H), 3.66 (dt, *J* = 11.1, 1.3 Hz, 1 H), 3.61–3.53 (m, 1 H), 3.40 (dd, *J* = 11.0, 6.3 Hz, 1 H), 3.29–3.19 (m, 2 H), 2.95–2.85 (m, 1 H), 2.50–2.38 (m, 1 H), 2.38–2.28 (m, 1 H), 2.00–1.88 (m, 1 H), 1.16 (t, *J* = 7.2 Hz, 3 H). **^{13}C NMR** [101 MHz, δ (ppm), CD_3OD]: 158.2, 66.5 (d, *J* = 14.9 Hz), 52.0, 51.3, 42.2, 35.0, 26.1, 20.7, 14.4. **^{19}F NMR** [377 MHz, δ (ppm), CD_3OD]: 37.9. ***R*F**: 0.45 (CH_2Cl_2/MeOH, 9:1). **FT-IR** (cm^{-1}, neat, ATR) ṽ = 3322, 1626, 1541, 1391, 1199, 740, 621. **HRMS** [ESI (*m*/*z*)] calcd. for $(C_9H_{15}FN_2O_3S + H)^+$ = 251.0860 found: 251.0861.

***rac*-(1*R*,5*S*)-3-(Phenylcarbamoyl)-3-azabicyclo[3.2.0]heptane-1-sulfonyl fluoride (1f)**

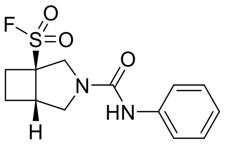

According to general procedure D, the reaction of secondary amine **12** (9.2 mg, 0.043 mmol, 1.0 equiv), Et_3N (16.5 µL, 0.12 mmol, 2.8 equiv), and phenyl isocyanate (10 µL, 0.092 mmol, 2.1 equiv) in anhydrous CH_2Cl_2 (0.25 mL) for 18 h afforded urea **1f** (9.4 mg, 31 µmol, 74%) as a white solid after column chromatography (dry loaded on silica, heptane → 1:1 heptane/AcOEt).

^{1}H NMR [400 MHz, δ (ppm), CD_3OD]: 7.46–7.40 (m, 2 H), 7.33–7.25 (m, 2 H), 7.08–7.01 (m, 1 H), 4.23 (d, *J* = 12.3 Hz, 1 H), 4.03 (dd, *J* = 12.3, 1.1 Hz, 1 H), 3.84 (dd, *J* = 11.0, 1.5 Hz, 1 H), 3.65–3.58 (m, 1 H), 3.54 (dd, *J* = 11.0, 6.3 Hz, 1 H), 2.96–2.87 (m, 1 H), 2.52–2.43 (m, 1 H), 2.41–2.31 (m, 1 H), 2.06–1.94 (m, 1 H). **^{13}C NMR** [101 MHz, δ (ppm), CD_3OD]: 155.9, 139.0, 128.2 (2 C), 123.1, 120.8 (2 C), 66.6 (d, *J* = 15.1 Hz), 52.1, 51.5, 42.2, 26.1, 20.7. **^{19}F NMR** [377 MHz, δ (ppm), CD_3OD]: 38.0 (SO_2*F*). ***R*F**: 0.22 (heptane/AcOEt, 3:2). **FT-IR** (cm^{-1}, neat, ATR) ṽ = 3284, 1633, 1593, 1529, 1447, 1392, 1244, 1204, 755, 627. **HRMS** [ESI (*m*/*z*)] calcd. for $(C_{13}H_{15}FN_2O_3S + Na)^+$ = 322.0713 found: 322.0710.

***rac*-(1*R*,5*S*)-*N*-Ethyl-1-(methylsulfamoyl)-3-azabicyclo[3.2.0]heptane-3-carboxamide (2ad)**

According to general procedure D, the reaction of secondary amine **14a** (6.9 mg, 0.030 mmol, 1.0 equiv), Et_3N (15.2 µL, 0.11 mmol, 3.6 equiv), and ethyl isocyanate (3.40 µL, 0.43 mmol, 1.4 equiv) in anhydrous THF (0.5 mL) for 16 h afforded urea **2ad** (3.2 mg, 12.2 µmol, 40%) as a white solid after column chromatography (CH_2Cl_2 → 9:1 CH_2Cl_2/MeOH) and prep HPLC.

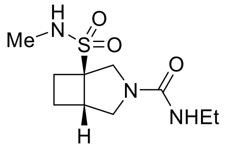

^{1}H NMR [500 MHz, δ (ppm), $CDCl_3$]: 4.58 (q, *J* = 5.2 Hz, 1 H), 4.35 (t, *J* = 5.4 Hz, 1 H), 3.93–3.85 (m, 2 H), 3.48–3.42 (m, 2 H), 3.42–3.38 (m, 1 H), 3.32 (qd, *J* = 7.2, 5.4 Hz, 2 H), 2.86 (d, *J* = 5.2 Hz, 3 H), 2.85–2.79 (m, 1 H), 2.39–2.34 (m, 1 H), 2.20–2.12 (m, 1 H), 1.86 (dddd, *J* = 11.8, 9.4, 8.0, 6.0 Hz, 1 H), 1.19 (t, *J* = 7.2 Hz, 3 H). **^{13}C NMR** [126 MHz, δ (ppm), $CDCl_3$]: 157.3, 68.0, 53.4, 52.0, 41.5, 35.6, 30.1, 27.1, 21.5, 15.6. ***R*$_F$**: 0.28 (CH_2Cl_2/MeOH, 9:1). **FT-IR** (cm^{-1}, neat, ATR) ṽ = 3399, 3143, 2172, 1629, 1540, 1310, 1117. **HRMS** [ESI (*m*/*z*)] calcd. for $(C_{10}H_{19}N_3O_3S + Na)^+$ = 284.1039 found: 284.1031.

***rac*-(1*R*,5*S*)-1-(Cyclopropylsulfamoyl)-*N*-ethyl-3-azabicyclo[3.2.0]heptane-3-carboxamide (2be)**

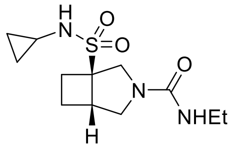

According to general procedure D, the reaction of secondary amine **14b** (10.0 mg, 0.040 mmol, 1.0 equiv), Et_3N (13.8 µL, 0.1 mmol, 2.5 equiv), and ethyl isocyanate (3.7 µL, 0.05 mmol, 1.2 equiv) in anhydrous CH_2Cl_2 (0.3 mL) for 19 h, additional ethyl isocyanate (2.0 µL, 0.025 mmol, 0.6 equiv) was added and the reaction mixture was stirred at 40 °C for 2 h, afforded urea **2be** (6.9 mg, 24 µmol, 61%) as a white solid after column chromatography (CH_2Cl_2 → 9:1 CH_2Cl_2/MeOH) and prep HPLC.

^{1}H NMR [500 MHz, δ (ppm), $CDCl_3$]: 5.16 (s, 1 H), 4.35 (t, *J* = 5.6 Hz, 1 H), 3.94–3.86 (m, 2 H), 3.48–3.43 (m, 1 H), 3.43–3.39 (m, 2 H), 3.30 (qd, *J* = 7.2, 5.4 Hz, 2 H), 2.93–2.82 (m, 1 H), 2.62–2.52 (m, 1 H), 2.40–2.31 (m, 1 H), 2.20–2.11 (m, 1 H), 1.85 (dddd, *J* = 12.2, 9.6, 8.1, 6.0 Hz, 1 H), 1.17 (t, *J* = 7.2 Hz, 3 H), 0.78–0.59 (m, 4 H). **^{13}C NMR** [126 MHz, δ (ppm), $CDCl_3$]: 157.3, 67.5, 53.6, 52.1, 41.4, 35.6, 27.2, 24.7, 21.5, 15.6, 7.5, 7.0. ***R*$_F$**: 0.38 (CH_2Cl_2/MeOH, 9:1). **FT-IR** (cm^{-1}, neat, ATR) ṽ = 3402, 3124, 2175, 1626, 1535, 1308, 1144, 1033, 717. **HRMS** [ESI (*m*/*z*)] calcd. for $(C_{12}H_{21}N_3O_3S + H)^+$ = 288.1376 found: 288.1375.

***rac*-(1*R*,5*S*)-1-(Cyclobutylsulfamoyl)-*N*-methyl-3-azabicyclo[3.2.0]heptane-3-carboxamide (2cf)**

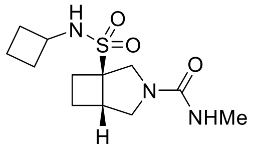

According to general procedure D (using *N*-succinimidyl *N*-methylcarbamate instead of methyl isocyanate), the reaction of secondary amine **14c** (10 mg, 0.04 mmol, 1.0 equiv), Et_3N (13.1 µL, 0.1 mmol, 2.5 equiv), and succinimidyl *N*-methylcarbamate (8.0 mg, 0.046 mmol, 1.2 equiv) in anhydrous CH_2Cl_2 (0.3 mL) for 19 h afforded urea **2cf** (6.2 mg, 21.6 µmol, 58%) as a white solid after column chromatography (CH_2Cl_2 → 9:1 CH_2Cl_2/MeOH) and prep HPLC.

^{1}H NMR [500 MHz, δ (ppm), $CDCl_3$]: 5.19 (d, *J* = 9.2 Hz, 1 H), 4.44 (q, *J* = 4.8 Hz, 1 H), 4.01–3.89 (m, 1 H), 3.86–3.79 (m, 2 H), 3.46–3.37 (m, 2 H), 3.36–3.28 (m, 1 H), 2.84 (d, *J* = 4.7 Hz, 3 H), 2.80–2.70 (m, 1 H), 2.40–2.22 (m, 3 H), 2.09 (ddd, *J* = 12.1, 9.4, 4.9 Hz, 1 H), 2.04–1.89 (m, 2 H), 1.84–1.75 (m, 1 H), 1.70–1.51 (m, 2 H). **^{13}C NMR** [126 MHz, δ (ppm), $CDCl_3$]: 158.0, 67.8, 53.5, 52.2, 49.0, 41.3, 33.1, 32.7, 27.5, 26.9, 21.1, 14.2. ***R*$_F$**: 0.33 (CH_2Cl_2/MeOH, 9:1). **FT-IR** (cm^{-1}, neat, ATR) ṽ = 3391, 3101, 1625, 1541, 1367, 1304, 1135, 1115, 620. **HRMS** [ESI (*m*/*z*)] calcd. for $(C_{12}H_{21}N_3O_3S + H)^+$ = 288.1376 found: 288.1375.

General procedure E[7]

The indicated alkyl bromide (1.3 equiv) was added to a mixture of secondary amine **12** and K_2CO_3 (2.5 equiv) in anhydrous MeCN. The reaction mixture was stirred at 21 °C for 20–22 h. The mixture was diluted with AcOEt (10 mL) and washed with 0.1 M aqueous K_2CO_3 solution (10 mL). The organic layer was dried over Na_2SO_4, filtered, the solvent was removed *in vacuo*, and the product was purified by either column chromatography or prep HPLC using the indicated eluent to afford tertiary amines **1g** and **1h**.

rac-(1_R_,5_S_)-3-(Pyridin-4-ylmethyl)-3-azabicyclo[3.2.0]heptane-1-sulfonyl fluoride (1g)

According to general procedure E, the reaction of secondary amine **12** (14.9 mg, 0.069 mmol, 1.0 equiv), K_2CO_3 (24.2 mg, 0.18 mmol, 2.5 equiv), and 4-(bromomethyl)pyridine hydrobromide (23.0 mg, 0.09 mmol, 1.3 equiv) in anhydrous MeCN (1.0 mL) for 22 h afforded tertiary amine **1g** (0.6 mg, 2.2 µmol, 3%) as a yellow oil after prep HPLC.

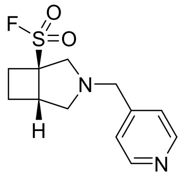

^{1}H NMR [500 MHz, δ (ppm), $CDCl_3$]: 8.66–8.50 (m, 2 H), 7.37–7.29 (m, 2 H), 3.77 (s, 2 H), 3.36 (dt, _J_ = 10.1, 5.5 Hz, 1 H), 3.18 (d, _J_ = 9.5 Hz, 1 H), 2.94 (dd, _J_ = 9.7, 1.7 Hz, 1 H), 2.91–2.82 (m, 1 H), 2.75 (d, _J_ = 9.6 Hz, 1 H), 2.46 (dd, _J_ = 9.7, 5.7 Hz, 1 H), 2.43–2.31 (m, 2 H), 2.03–1.89 (m, 1 H). **^{13}C NMR*** [126 MHz, δ (ppm), $CDCl_3$]: 150.1, 123.5, 59.4 (2 C), 57.8, 41.7, 26.6, 21.2. **^{19}F NMR** [471 MHz, δ (ppm), $CDCl_3$]: 40.5 (SO_2F). **R_F**: 0.57 (CH_2Cl_2/MeOH, 9:1). **FT-IR** (cm^{-1}, neat, ATR) ṽ = 1602, 1394, 1203, 761. **HRMS** [ESI (_m/z_)] calcd. for $(C_{12}H_{15}FN_2O_2S + H)^+$ = 271.0911 found: 271.0897.

*Not all carbon peaks are observed.

rac-(1_R_,5_S_)-3-[(3-Methylisoxazol-5-yl)methyl]-3-azabicyclo[3.2.0]heptane-1-sulfonyl fluoride (1h)

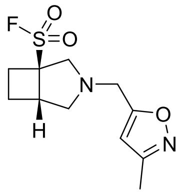

According to general procedure E, the reaction of secondary amine **12** (14.9 mg, 0.069 mmol, 1.0 equiv), K_2CO_3 (24 mg, 0.17 mmol, 2.5 equiv), and 5-(bromomethyl)-3-methylisoxazole (10.7 µL, 0.09 mmol, 1.3 equiv) in anhydrous MeCN (0.5 mL) for 20 h afforded tertiary amine **1h** (9.9 mg, 36.1 µmol, 52%) as a white solid after column chromatography (heptane → 1:1 heptane/AcOEt).

^{1}H NMR [400 MHz, δ (ppm), $CDCl_3$]: 6.05 (s, 1 H), 3.89 (s, 2 H), 3.39–3.29 (m, 1 H), 3.23 (d, _J_ = 9.5 Hz, 1 H), 2.99 (dd, _J_ = 9.6, 1.8 Hz, 1 H), 2.88–2.83 (m, 1 H), 2.81 (d, _J_ = 9.7 Hz, 1 H), 2.52 (dd, _J_ = 9.6, 5.7 Hz, 1 H), 2.40–2.33 (m, 2 H), 2.31 (s, 3 H), 1.96–1.85 (m, 1 H). **^{13}C NMR** [101 MHz, δ (ppm), $CDCl_3$]: 168.6, 159.8, 103.8, 66.5 (d, _J_ = 14.5 Hz), 59.1, 58.9, 49.1, 41.6, 26.4, 21.0, 11.5. **^{19}F NMR** [377 MHz, δ (ppm), $CDCl_3$]: 40.3. **R_F**: 0.26 (heptane/AcOEt, 3:2). **FT-IR** (cm^{-1}, neat, ATR) ṽ = 2826, 1603, 1390, 1203, 807, 745, 609. **HRMS** [ESI (_m/z_)] calcd. for $(C_{11}H_{15}FN_2O_3S + H)^+$ = 275.0860 found: 275.0861.

rac-(1_R_,5_S_)-3-(3-Methoxyphenyl)-_N_-methyl-3-azabicyclo[3.2.0]heptane-1-sulfonamide (2ag) [8]

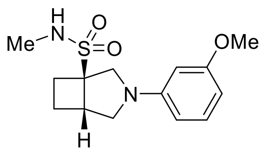

3-Bromoanisole (4.2 µL, 0.033 mmol, 1.2 equiv) and NaO^{t}Bu (2 M solution in THF, 32 µL, 0.064 mmol, 2.3 equiv) were added to a solution of secondary amine **14a** (6.3 mg, 28 µmol, 1.0 equiv), $Pd(OAc)_2$ (0.1 mg, 0.45 µmol, 0.016 equiv), and RuPhos (0.4 mg, 0.86 µmol, 0.031 equiv) in anhydrous THF (0.5 mL). The reaction mixture was stirred at 70 °C for 20 h. The reaction mixture was then diluted with CH_2Cl_2 (10 mL) and washed with a 1 M aq. NaOH solution (5 mL) and water (5 mL). The organic phase was dried over Na_2SO_4 and filtered. The solvent was removed _in vacuo_ to afford tertiary amine **2ag** (5.1 mg, 0.017 µmol, 62%) as a light-yellow oil after column chromatography (dry loaded on basic Al_2O_3, heptane → 1:1 heptane/AcOEt).

^{1}H NMR [500 MHz, δ (ppm), $CDCl_3$]: 7.19 (t, _J_ = 8.2 Hz, 1 H), 6.39 (dd, _J_ = 8.1, 2.3 Hz, 1 H), 6.35 (dd, _J_ = 8.1, 2.3 Hz, 1 H), 6.27 (t, _J_ = 2.4 Hz, 1 H), 4.20–4.13 (m, 1 H), 3.81 (s, 3 H), 3.79 (d, _J_ = 10.2 Hz, 1 H), 3.57–3.54 (m, 1 H), 3.53 (d, _J_ = 9.8 Hz, 1 H), 3.40 (dt, _J_ = 9.4, 6.0 Hz, 1 H), 3.25 (dd, _J_ = 9.8, 6.3 Hz, 1 H), 2.87 (d, _J_ = 5.2 Hz, 3 H), 2.90–2.78 (m, 1 H), 2.37 (tdd, _J_ = 11.3, 9.3, 5.5 Hz, 1 H), 2.21 (ddd, _J_ = 12.4, 9.6, 5.5 Hz, 1 H), 1.87 (dddd, _J_ = 12.0, 9.7, 7.5, 5.8 Hz, 1 H). **^{13}C NMR** [126 MHz, δ (ppm), $CDCl_3$]: 160.7, 149.8, 130.1, 106.9, 103.1, 100.5, 68.4, 56.12, 55.24, 55.2, 40.7, 30.4, 27.2, 21.7. **R_F**: 0.37 (heptane/AcOEt, 1:1). **FT-IR** (cm^{-1}, neat, ATR) ṽ = 3298, 1608, 1576, 1496, 1307, 1210, 1146, 1120, 828, 599. **HRMS** [ESI (_m/z_)] calcd. for $(C_{14}H_{20}N_2O_3S + Na)^+$ = 319.1087 found: 319.1096.

rac-(1_R_,5_S_)-3-Cyclohexyl-_N_-cyclopropyl-3-azabicyclo[3.2.0]heptane-1-sulfonamide (2bh) [9]

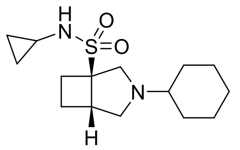

$NaBH(OAc)_3$ (12.4 mg, 0.06 mmol, 1.5 equiv) and acetic acid (2.2 µL, 0.04 mmol, 1.0 equiv) were added to a solution of secondary amine **14b** (9.9 mg, 0.04 mmol, 1.0 equiv), DIPEA (6.8 µL, 0.039 mmol, 1.0 equiv), and cyclohexanone (4.5 µl, 0.043 mmol, 1.1 equiv) in anhydrous THF (0.5 mL). The reaction mixture was stirred at 21 °C for 24 h. The mixture was quenched with a 1 M aq. NaOH solution (5 mL) and three times extracted with AcOEt (5 mL). The combined organic layers were dried over Na_2SO_4 and filtered. The solvent was removed _in vacuo_ to afford tertiary amine **2bh** (6.3 mg, 21.1 µmol, 54%) as a white solid after column chromatography (dry loaded on basic Al_2O_3, heptane → 1:1 heptane/AcOEt).

^{1}H NMR [500 MHz, δ (ppm), $CDCl_3$]: 4.60 (s, 1 H), 3.25–3.12 (m, 2 H), 3.05–2.95 (m, 1 H), 2.83–2.73 (m, 1 H), 2.71–2.62 (m, 1 H), 2.61–2.54 (m, 1 H), 2.42–2.31 (m, 1 H), 2.30–2.11 (m, 2 H), 1.92–1.85 (m, 3 H), 1.81–1.74 (m, 2 H), 1.62–1.56 (m, 1 H), 1.45–1.18 (m, 5 H), 0.75–0.65 (m, 4 H). **^{13}C NMR*** [126 MHz, δ (ppm), $CDCl_3$]: 67.4, 58.3, 57.4, 40.1, 26.8, 26.0, 24.8, 24.66 (2 C, 26.63 (2 C), 21.3, 7.5, 7.2. ***R*$_F$**: 0.10 (heptane/AcOEt, 3:2). **FT-IR** (cm^{-1}, neat, ATR) ṽ = 2929, 1449, 1308, 1146, 1127, 1026. **HRMS** [ESI (*m*/*z*)] calcd. for $(C_{15}H_{26}N_2O_2S + H)^+$ = 299.1788 found: 299.1789.

*Not all carbon peaks are observed.

***rac*-2-{(1*R*,5*S*)-1-(Cyclobutylsulfamoyl)-3-azabicyclo[3.2.0]heptan-3-yl}acetic acid (2ci)** [7]

2-Bromoacetic acid (7.8 mg, 0.056 mmol, 1.1 equiv) was added to a solution of secondary amine **14c** (13.5 mg, 0.05 mmol, 1.0 equiv) and DIPEA (26.5 µL, 0.15 mmol, 3.0 equiv) in anhydrous THF (0.5 mL). The reaction mixture was stirred at 21 °C for 24 h. The mixture was filtered and the solvent was removed *in vacuo* to afford tertiary amine **2ci** (4.6 mg, 13.8 µmol, 27%) as a white solid and after prep HPLC.

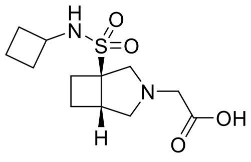

^{1}H NMR [500 MHz, δ (ppm), CD_3OD]: 3.93 (p, *J* = 8.4 Hz, 1 H), 3.67 (s, 2 H), 3.68–3.64 (m, 1 H), 3.50 (d, *J* = 11.1 Hz, 1 H), 3.29–3.22 (m, 2 H, C*H*), 3.03 (dd, *J* = 11.1, 6.7 Hz, 1 H), 2.75–2.64 (m, 1 H), 2.34–2.19 (m, 4 H), 2.04–1.94 (m, 2 H), 1.94–1.87 (m, 1 H), 1.70–1.54 (m, 2 H). **^{13}C NMR** [126 MHz, δ (ppm), CD_3OD]: 170.1, 67.4, 59.79 , 59.76, 56.6, 48.6, 40.0, 32.3, 31.9, 25.1, 19.5, 13.5. **FT-IR** (cm^{-1}, neat, ATR) ṽ = 2946, 1618, 1397, 1131, 1100, 908, 728. **HRMS** [ESI (*m*/*z*)] calcd. for $(C_{12}H_{20}N_2O_4S + H)^+$ = 289.1217 found: 289.1223.

2) Optimization for the Formation of Sulfonamides

We screened several reaction conditions to optimize this transformation (Table 1). Initially, we employed the conditions we had reported previously,[1] using DBU in PhMe at 110 °C for 24–48 h.[10] However, no conversion to sulfonamide **13f** was observed with pyrrolidine as the nucleophile (entry 1), possibly because of steric hindrance. Similarly, cyclopropanamine failed to react under these conditions (entry 2). Increasing the temperature via microwave irradiation (entry 3) did not yield the desired product either. Switching to the pair triethylamine/DMAP as the base/nucleophilic catalyst and methanol as the solvent (entry 4) provided sulfonamide **13b** in 4% yield, but changing the solvent to acetonitrile resulted in no conversion (entry 5). Methanamine (9.8 M solution in MeOH; entry 6) yielded sulfonamide **13a** in 35% yield, whereas ammonia (solution in MeOH; entry 7) failed to react. We next tested the methodology described by Mahapatra *et al.*,[3] using $Ca(NTf_2)_2$ and DABCO in THF. Cyclopropanamine and pyrrolidine (entries 8 and 9) yielded sulfonamides **13b** and **13f** in 6 and 10% yields, respectively. To further enhance the yields, we performed the reactions under hyperbaric conditions (15 kbar). For cyclopropanamine (entry 10), this change improved the yield to 72%, but for pyrrolidine (entry 11) remained low at 11%. Finaly, we applied the conditions described by Wei *et al.*[11], which employ HOBT and DIPEA for sterically hindered amines. Cyclopropanamine (entry 12) yielded the desired sulfonamide in 14% yield, aniline (entry 13) did not yield any product, and the secondary amines pyrrolidine and diethylamine (entries 14 and 15) gave sulfonamides **13f** and **13g** in low yields (18 and 1%, respectively). Given the comparable yields obtained with the Mahapatra and Wei methods, we proceeded with the Mahapatra methodology under hyperbaric conditions for subsequent reactions.

Table 1 Optimization of the formation of sulfonamides from sulfonyl fluoride **3**.

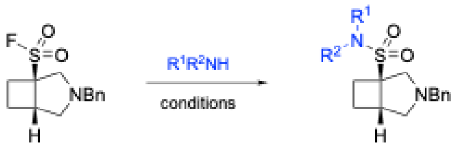

Entry	Amine (equiv)	Catalyst/Base (equiv)	Solvent	T (°C)	Pressure (bar)	Time (h)	Product	Yield (%)
1	Pyrrolidine (3.0)	DBU (2.5)	PhMe	110	1	20	**13f**	0
2	Cyclopropanamine (3.0)	DBU (2.0)	PhMe	110	1	70	**13b**	0
3[a]	Cyclopropanamine (3.0)	DBU (2.0)	PhMe	130–150	~2	4	**13b**	0
4	Cyclopropanamine (6.0)	Et_3N (1.0), DMAP (0.2)	MeOH	65	1	48	**13b**	4
5	Cyclopropanamine (3.0)	Et_3N (1.0), DMAP (0.2)	MeCN	70	1	44	**13b**	0
6	Methanamine (13)	–	MeOH	65	1	19	**13a**	35
7	Ammonia (12)	–	MeOH	65	1	22	**13h**	0
8	Cyclopropanamine (1.1)	$Ca(NTf_2)_2$ (1.1), DABCO (1.5)	THF	21	1	16	**13b**	6
9	Pyrrolidine (1.1)	$Ca(NTf_2)_2$ (1.1), DABCO (1.5)	THF	21	1	72	**13f**	10
10	Cyclopropanamine (1.1)	$Ca(NTf_2)_2$ (1.1), DABCO (1.5)	THF	21	15000	16	**13b**	72
11	Pyrrolidine (1.1)	$Ca(NTf_2)_2$ (1.1), DABCO (1.5)	THF	21	15000	16	**13f**	11
12	Cyclopropanamine (1.1)	HOBT (0.02), DIPEA (2.0)	DMSO	25	1	24	**13b**	14
13	Aniline (1.1)	HOBT (0.02), DIPEA (2.0)	DMSO	25	1	24	**13d**	0
14	Pyrrolidine (1.1)	HOBT (0.02), DIPEA (2.0)	DMSO	25	1	24	**13f**	18
15	Diethylamine (1.1)	HOBT (0.02), DIPEA (2.0)	DMSO	25	1	24	**13g**	1

[a] Reaction performed in the microwave.

3) ^{1}H and ^{13}C NMR Spectra

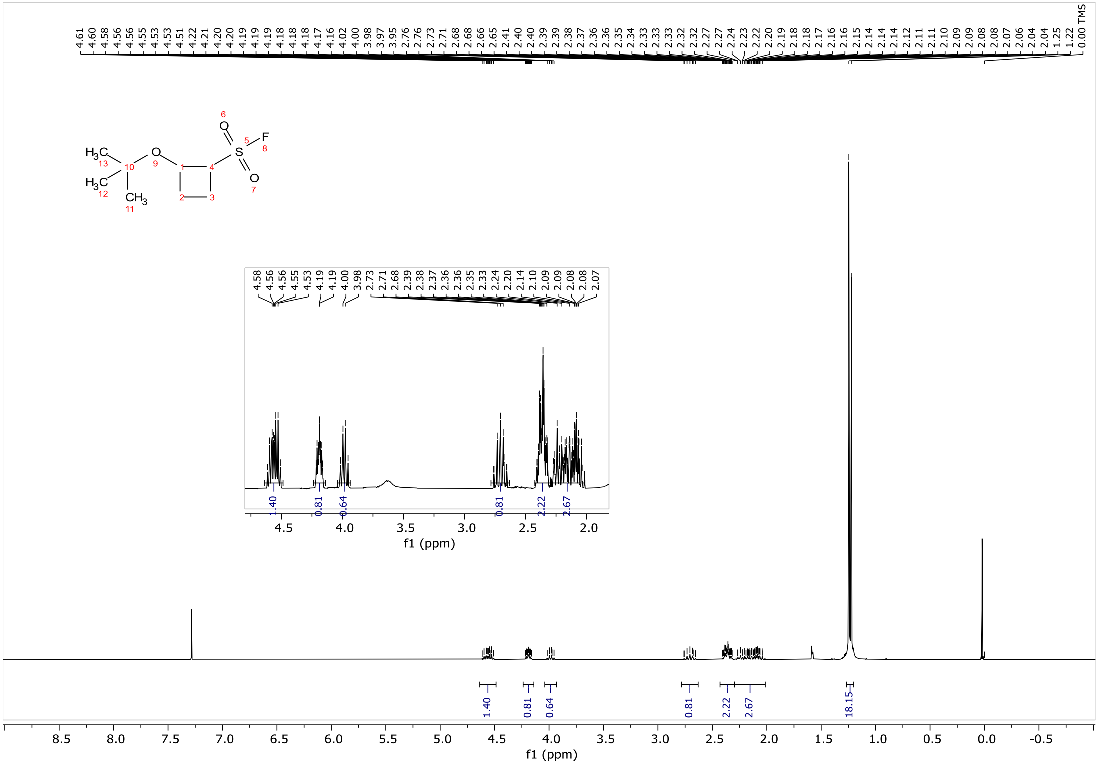

Figure S1 *^{1}H NMR spectrum (400 MHz, CDCl₃) of* ***5a*** *and* ***5b****.*

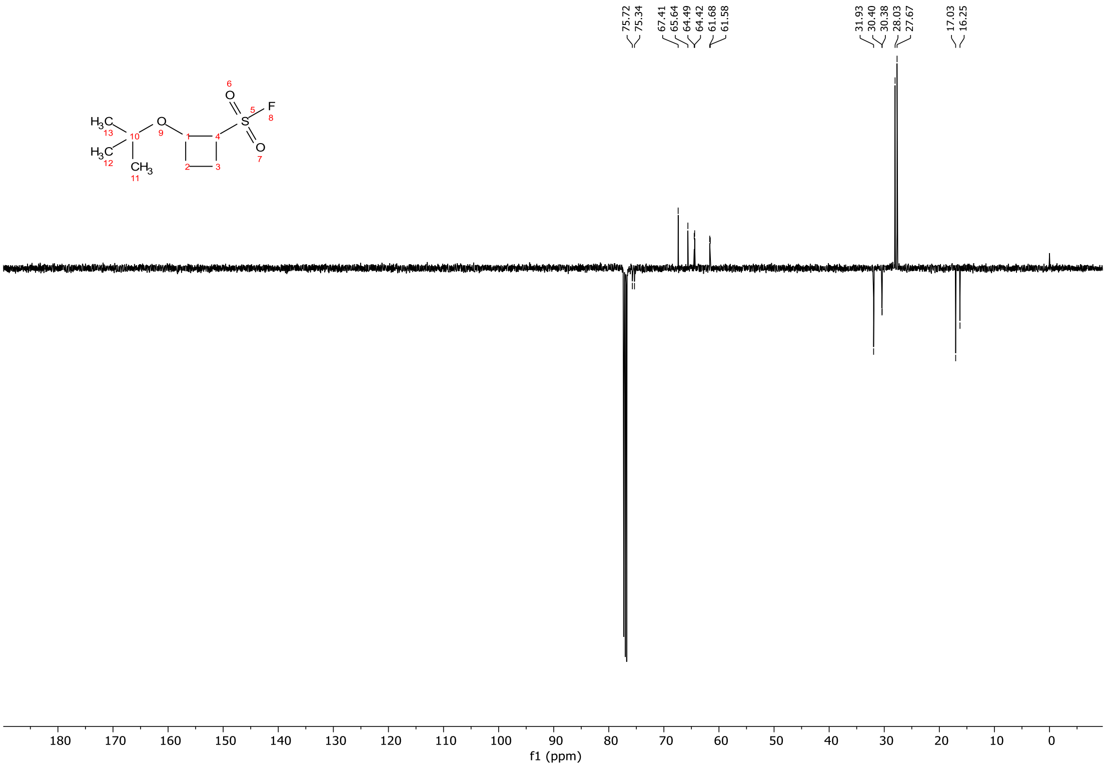

Figure S2 *^{13}C NMR spectrum (126 MHz, CDCl₃) of* ***5a*** *and* ***5b****.*

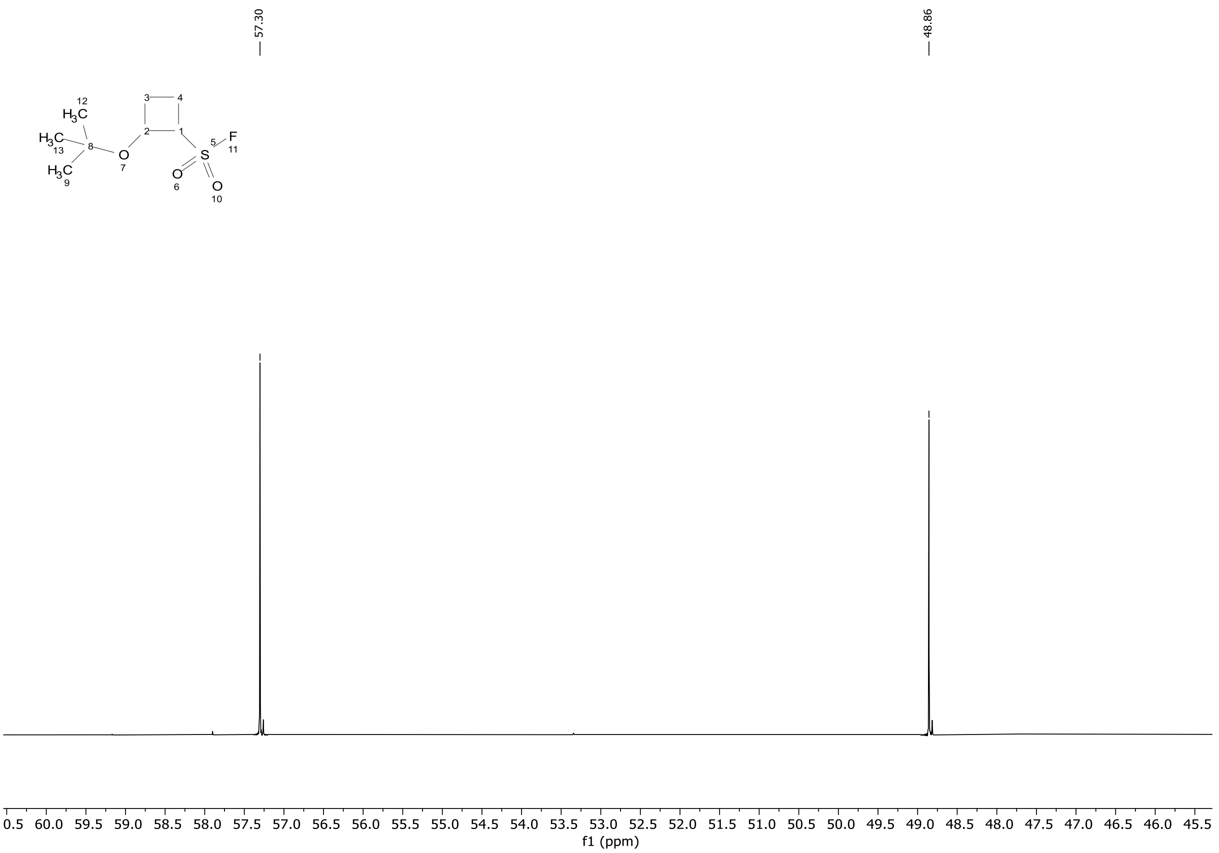

Figure S3 *^{19}F NMR spectrum (377 MHz, CDCl$_3$) of* ***5a*** *and* ***5b****.*

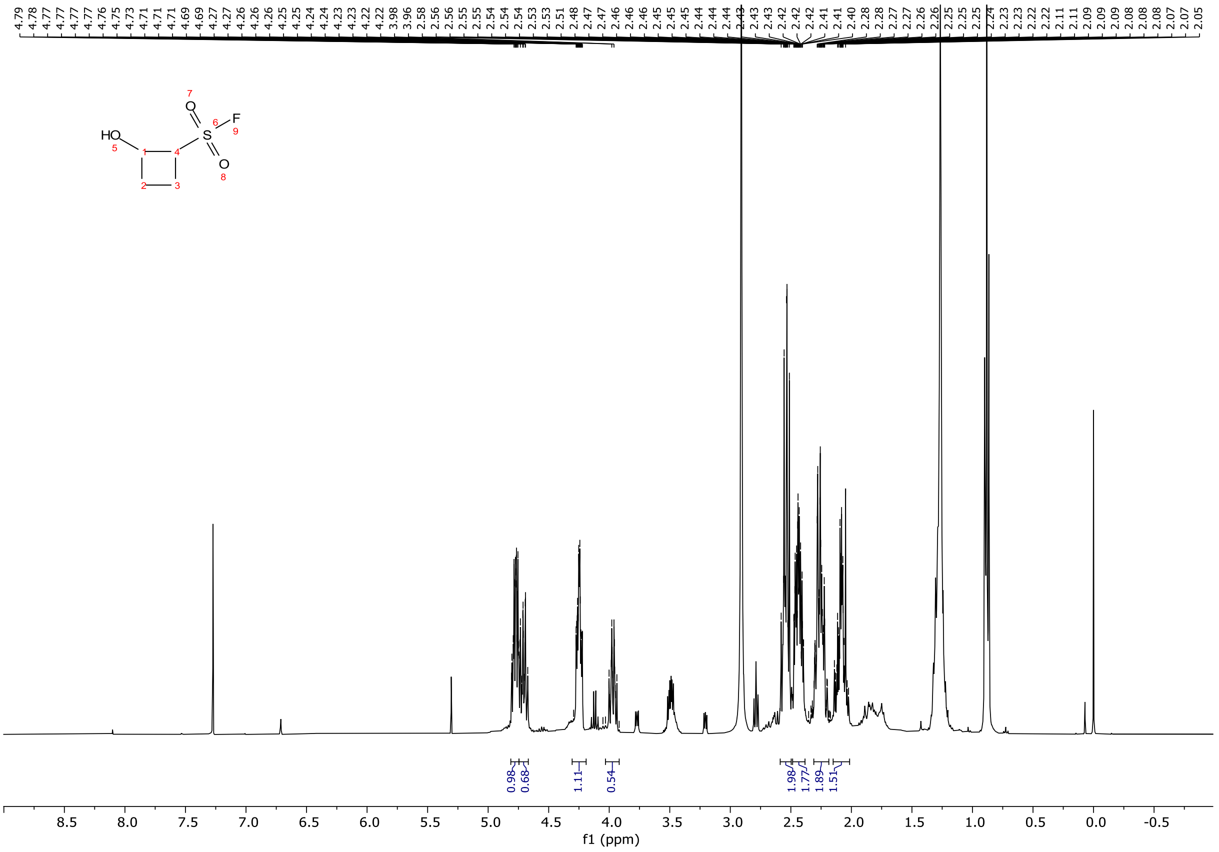

Figure ***S4*** *^{1}H NMR spectrum (400 MHz, CDCl$_3$) of* ***7a*** *and* ***7b****.*

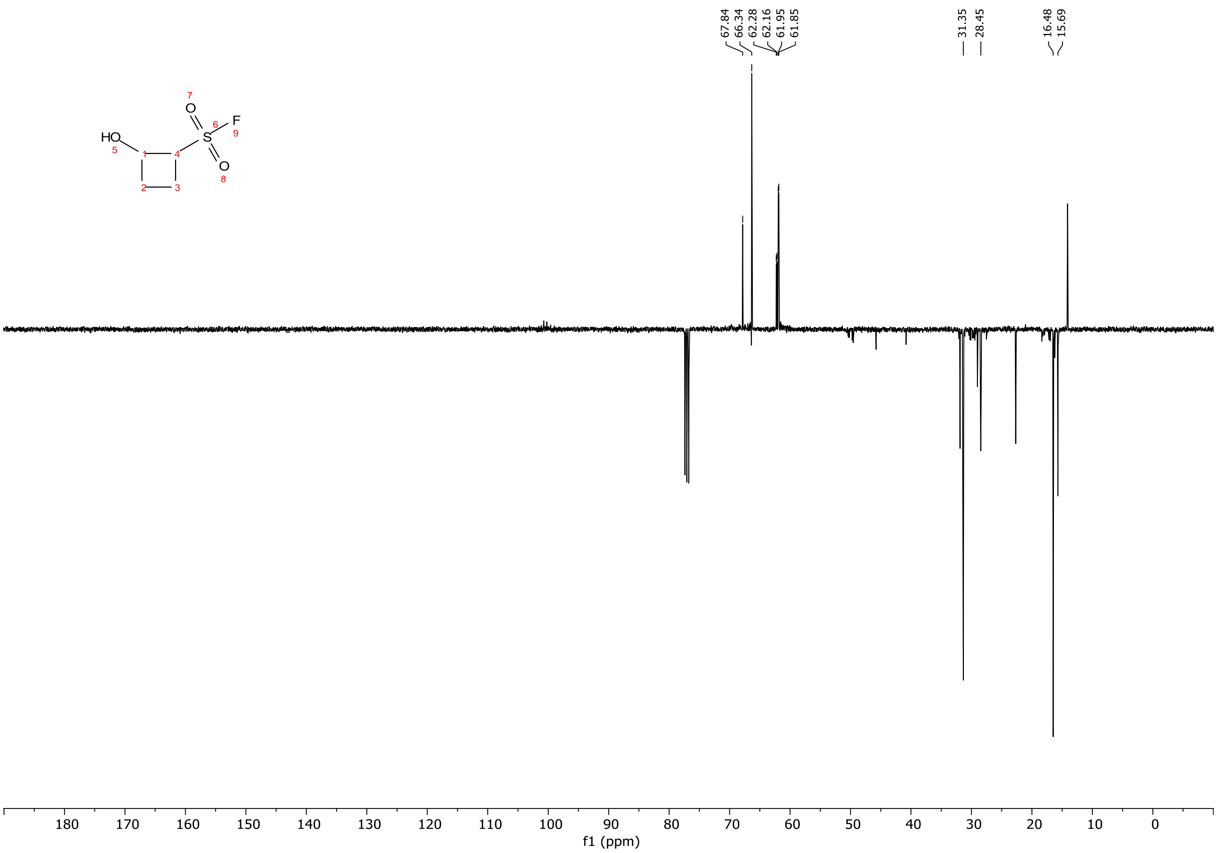

Figure S5 *^{13}C NMR spectrum (101 MHz, $CDCl_3$) of* ***7a*** *and* ***7b****.*

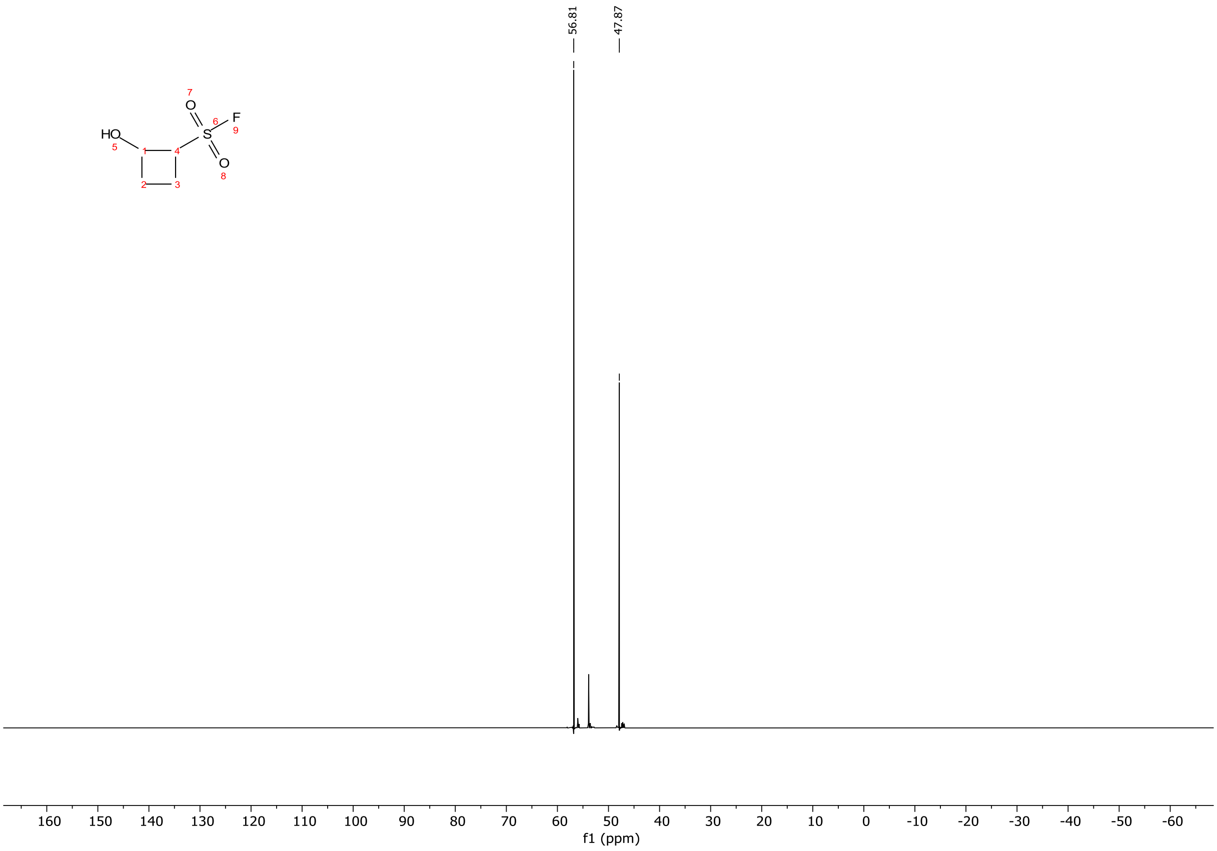

Figure S6 *^{19}F NMR spectrum (377 MHz, $CDCl_3$) of* ***7a*** *and* ***7b****.*

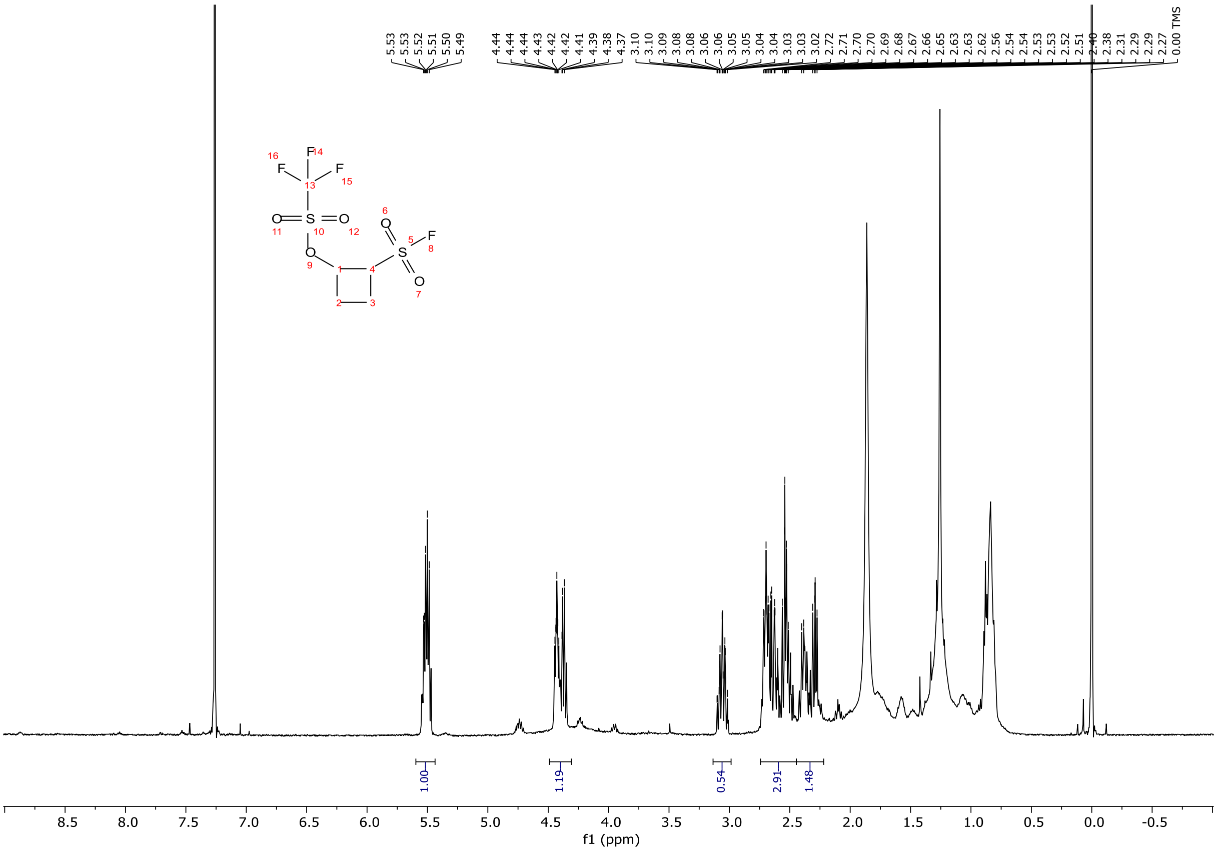

***Figure S7** ^{1}H NMR spectrum (500 MHz, $CDCl_3$) of **8a** and **8b**.*

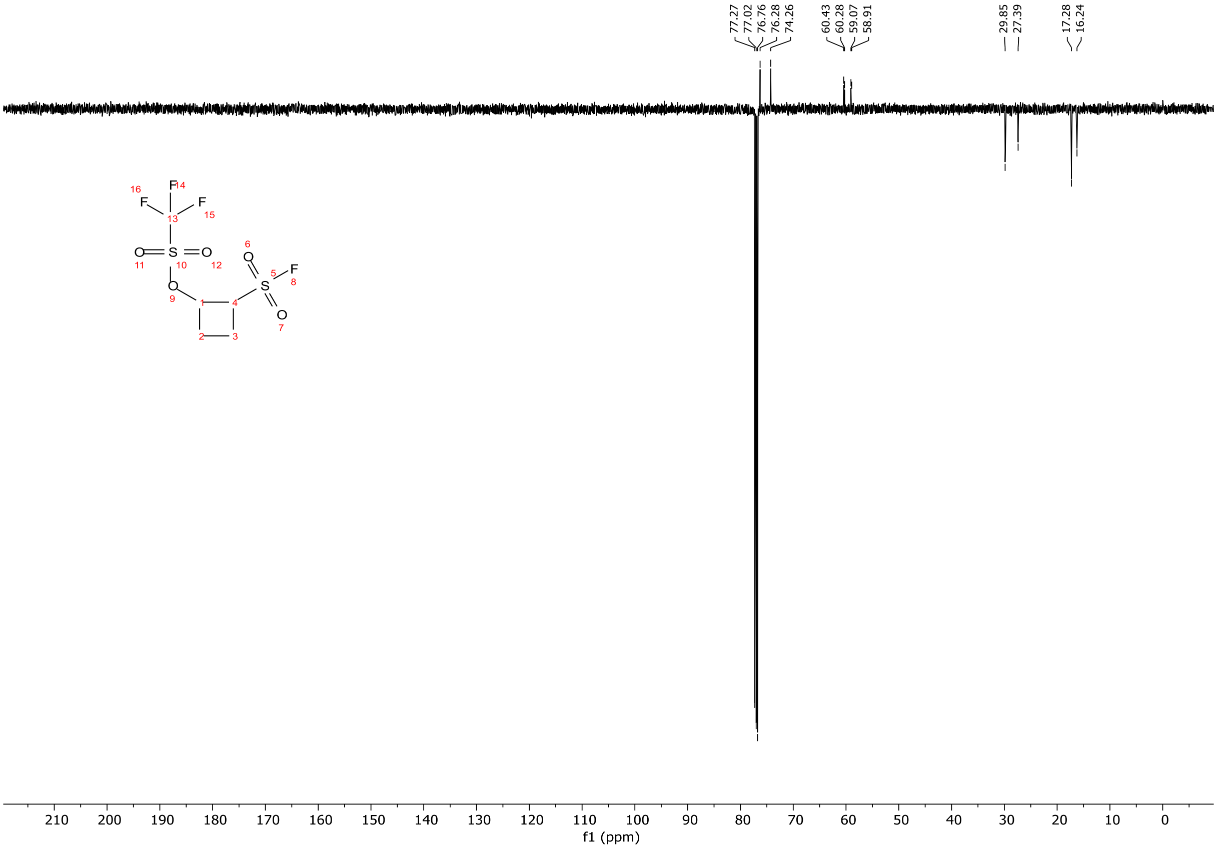

***Figure S8** ^{13}C NMR spectrum (126 MHz, $CDCl_3$) of **8a** and **8b**.*

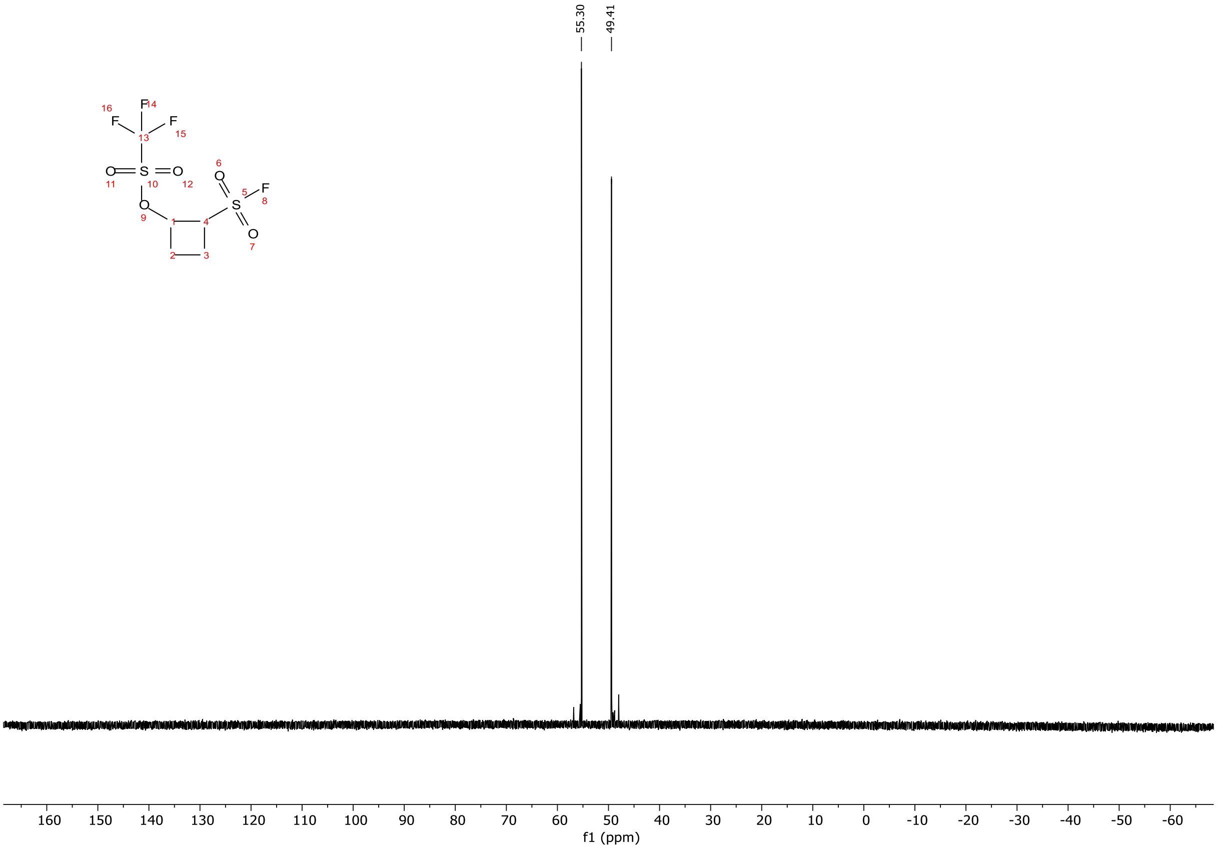

Figure S9 *^{19}F NMR spectrum (377 MHz, $CDCl_3$) of **8a** and **8b**.*

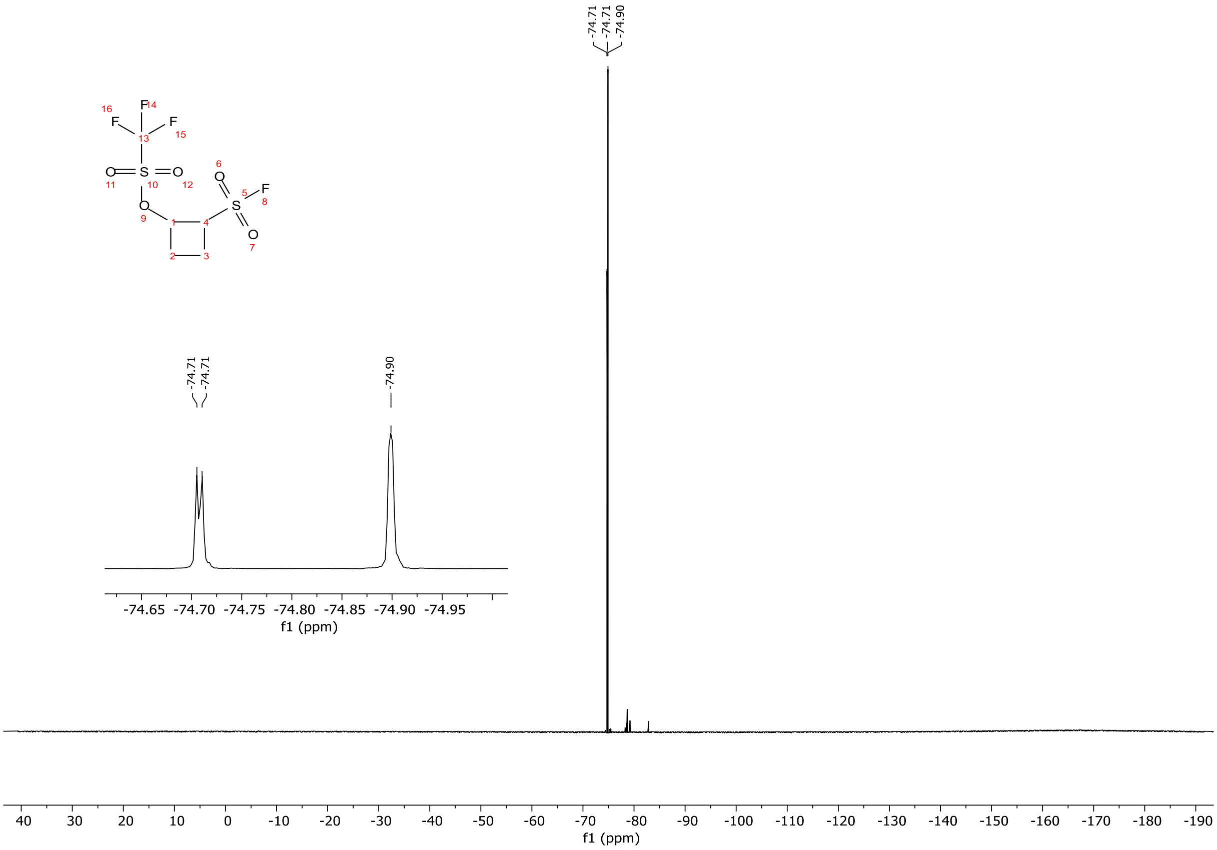

Figure S10 *^{19}F NMR spectrum (377 MHz, $CDCl_3$) of **8a** and **8b**.*

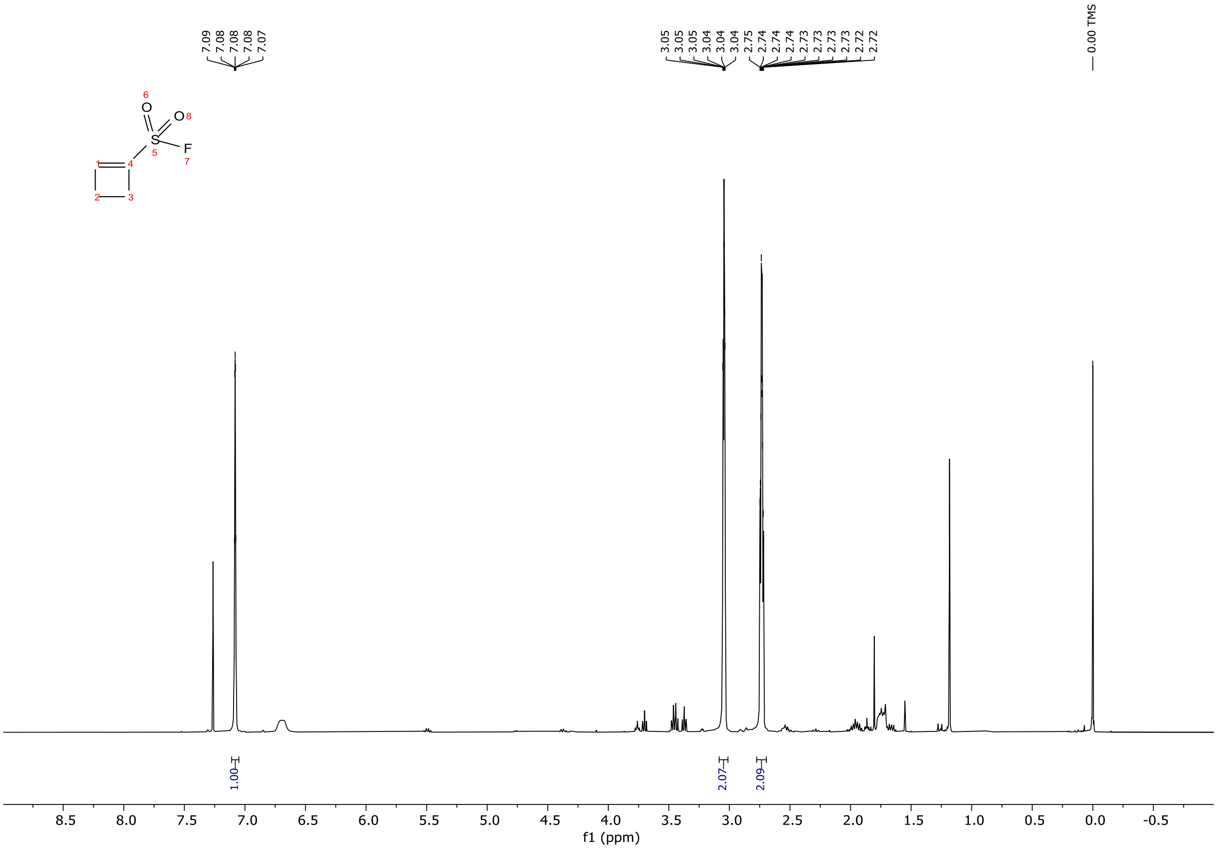

Figure S11 ^{1}*H NMR spectrum (400 MHz,* $CDCl_3$*) of* ***4****.*

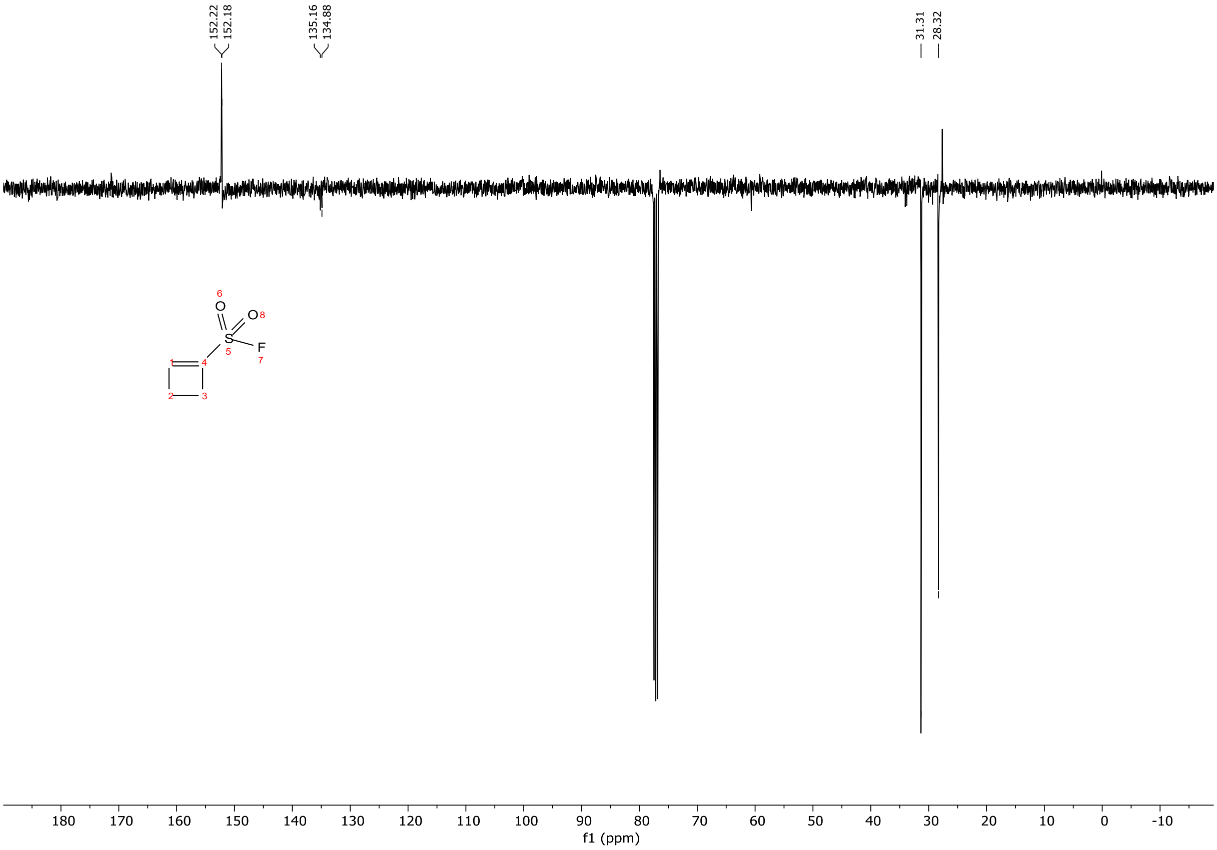

Figure S12 ^{13}*C NMR spectrum (101 MHz,* $CDCl_3$*) of* ***4****.*

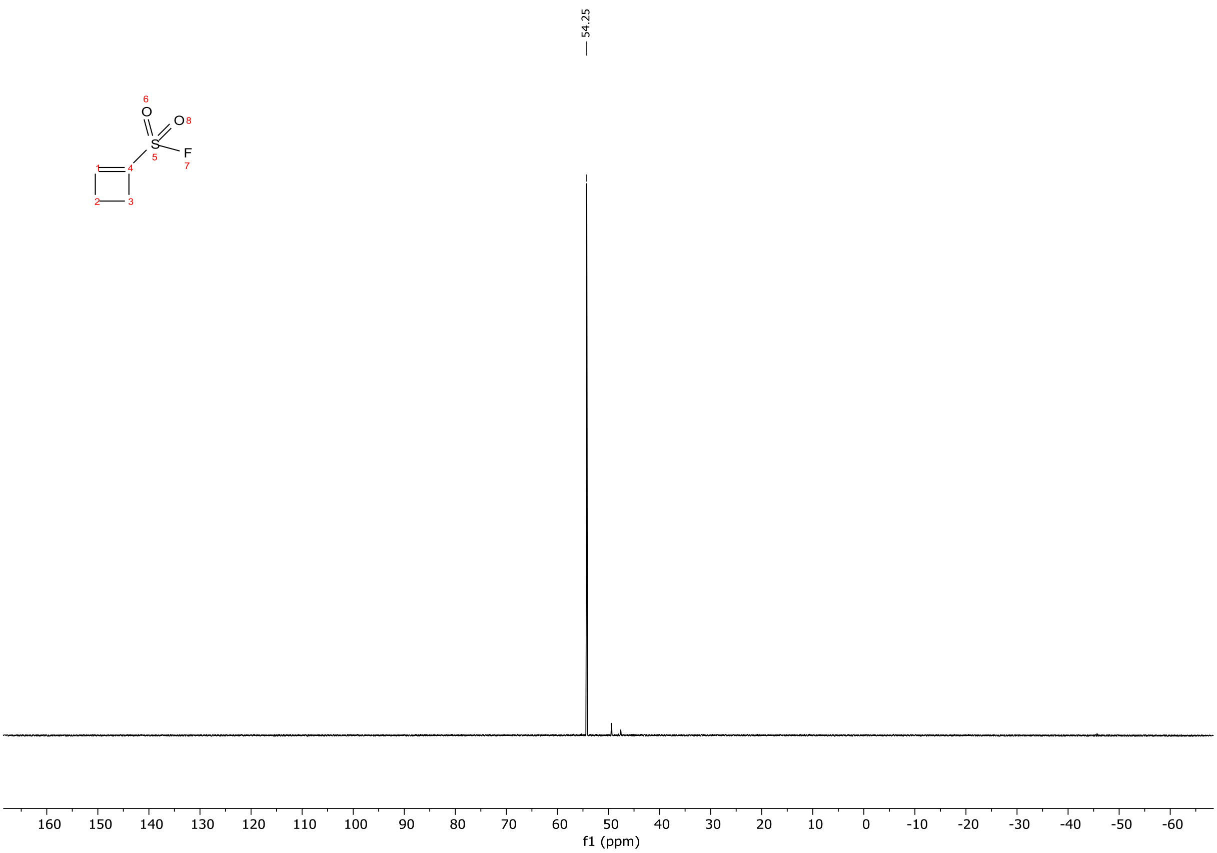

Figure S13 *^{19}F NMR spectrum (377 MHz, $CDCl_3$) of* ***4***.

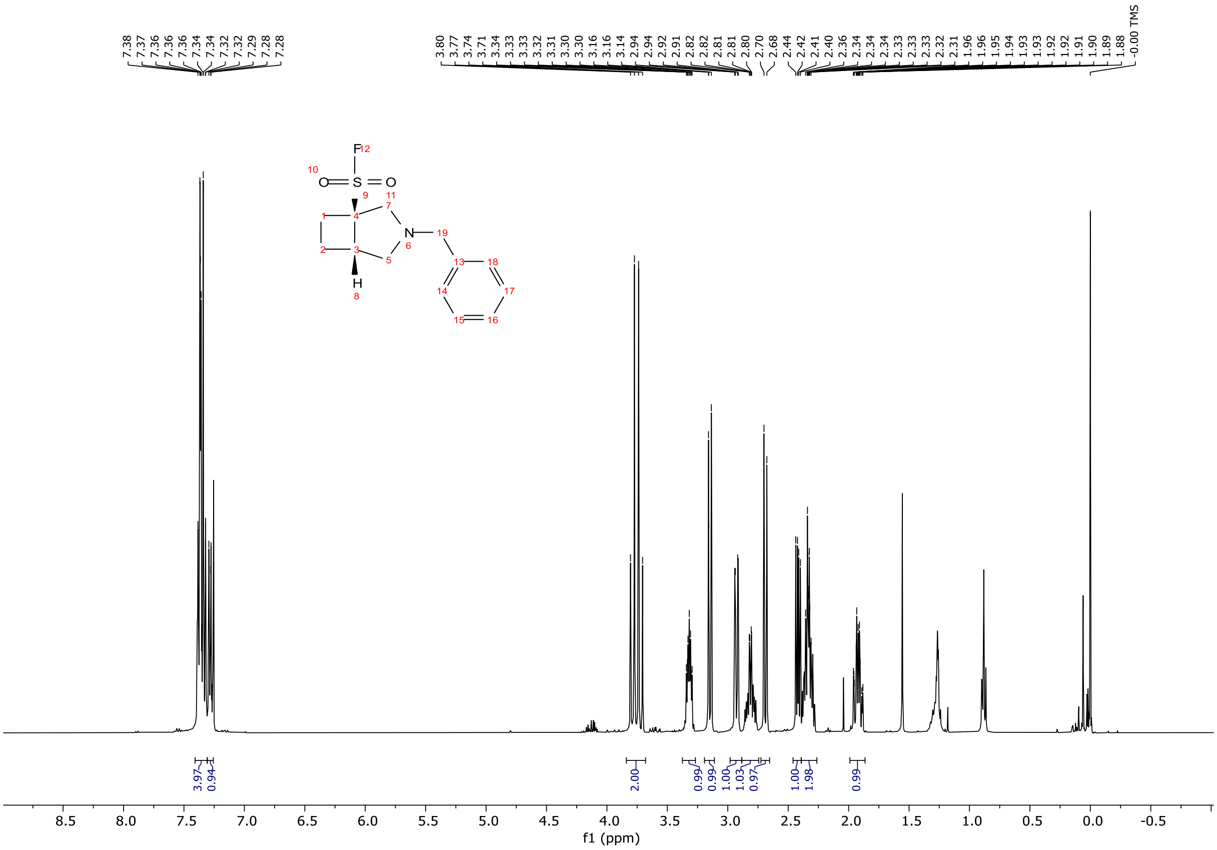

Figure S14 *^{1}H NMR spectrum (400 MHz, $CDCl_3$) of* ***3***.

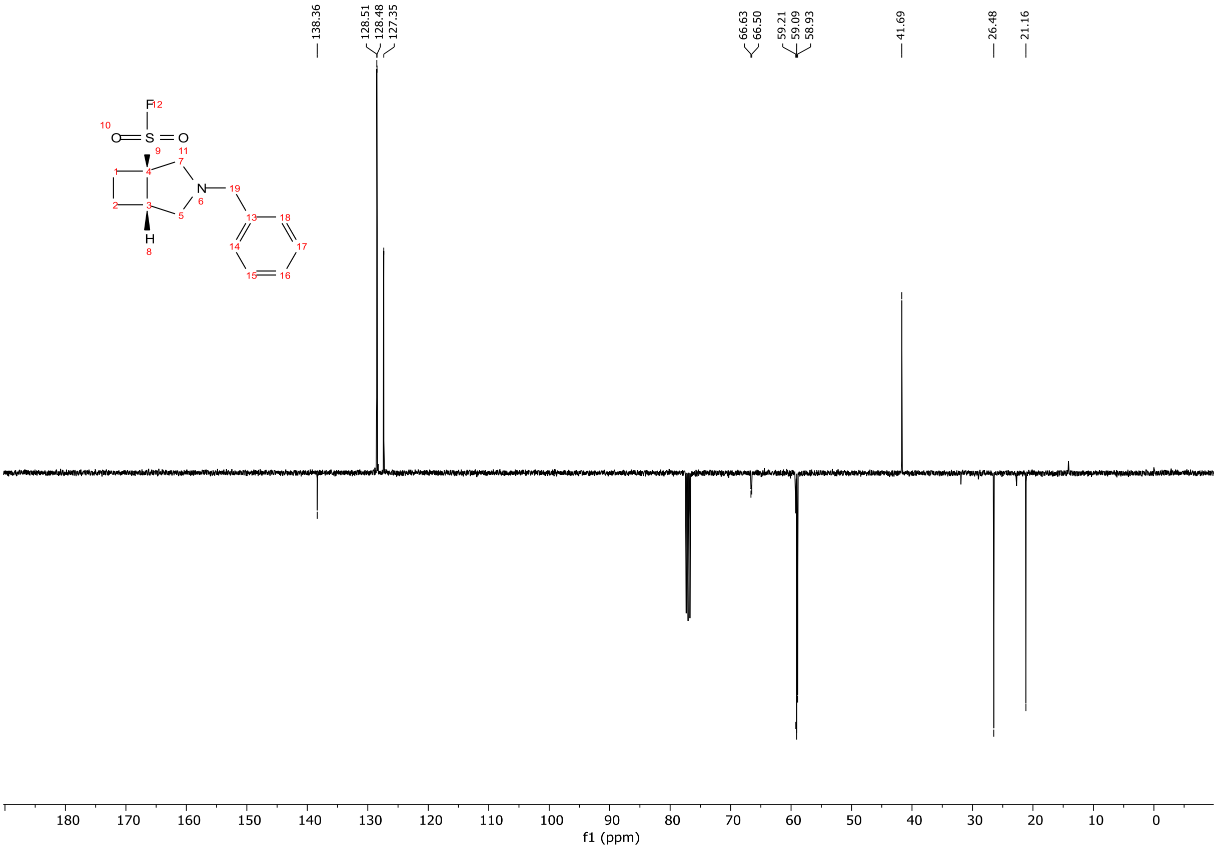

Figure S15 *^{13}C NMR spectrum (101 MHz, CDCl₃) of* ***3****.*

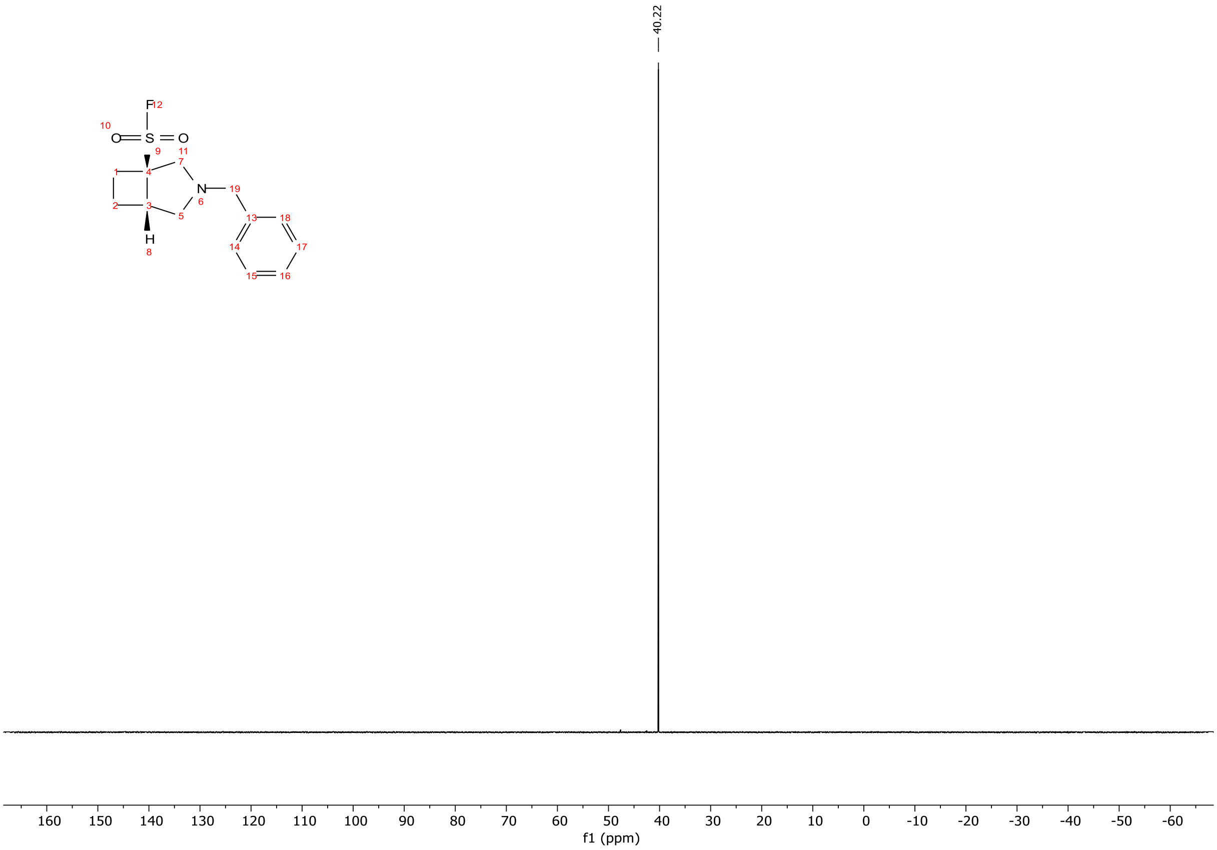

Figure S16 *^{19}F NMR (377 MHz, CDCl₃) spectrum of* ***3****.*

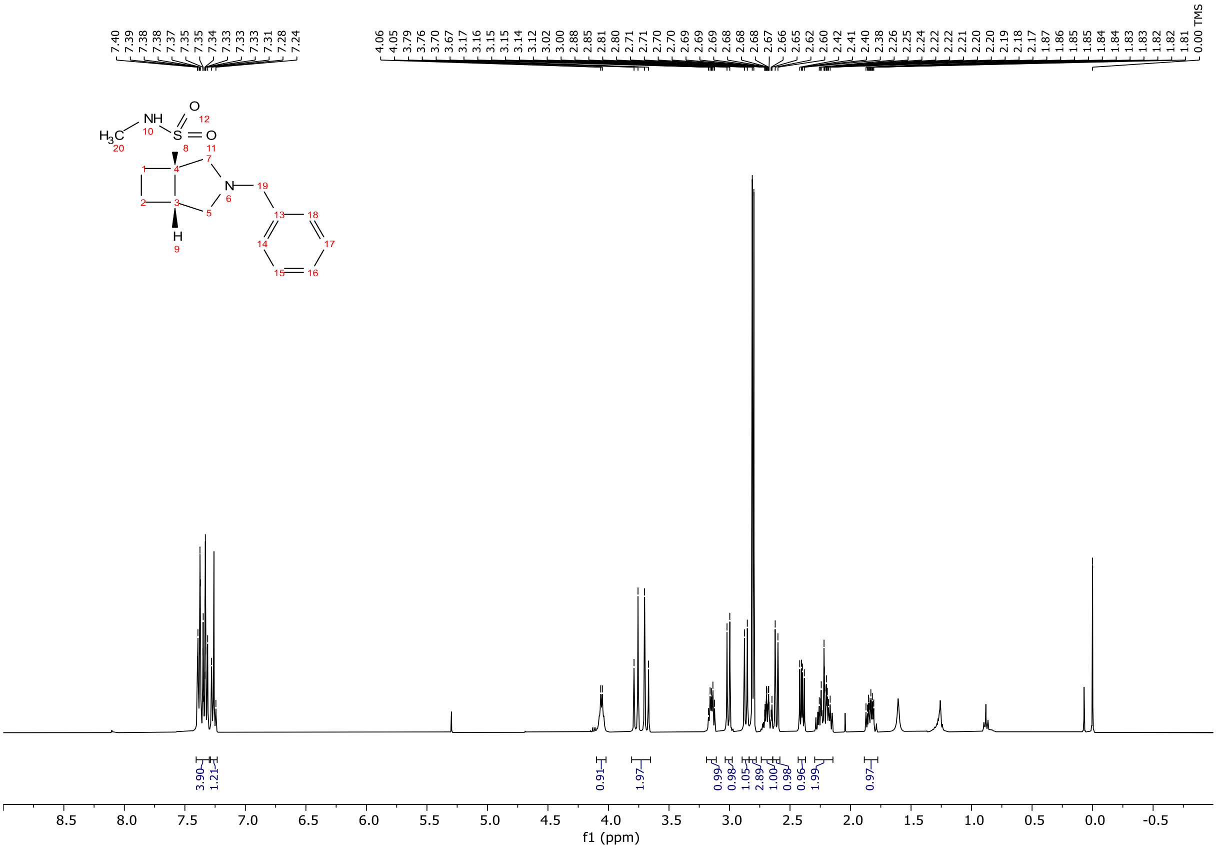

Figure S17 *^{1}H NMR spectrum (400 MHz, $CDCl_3$) of **13a**.*

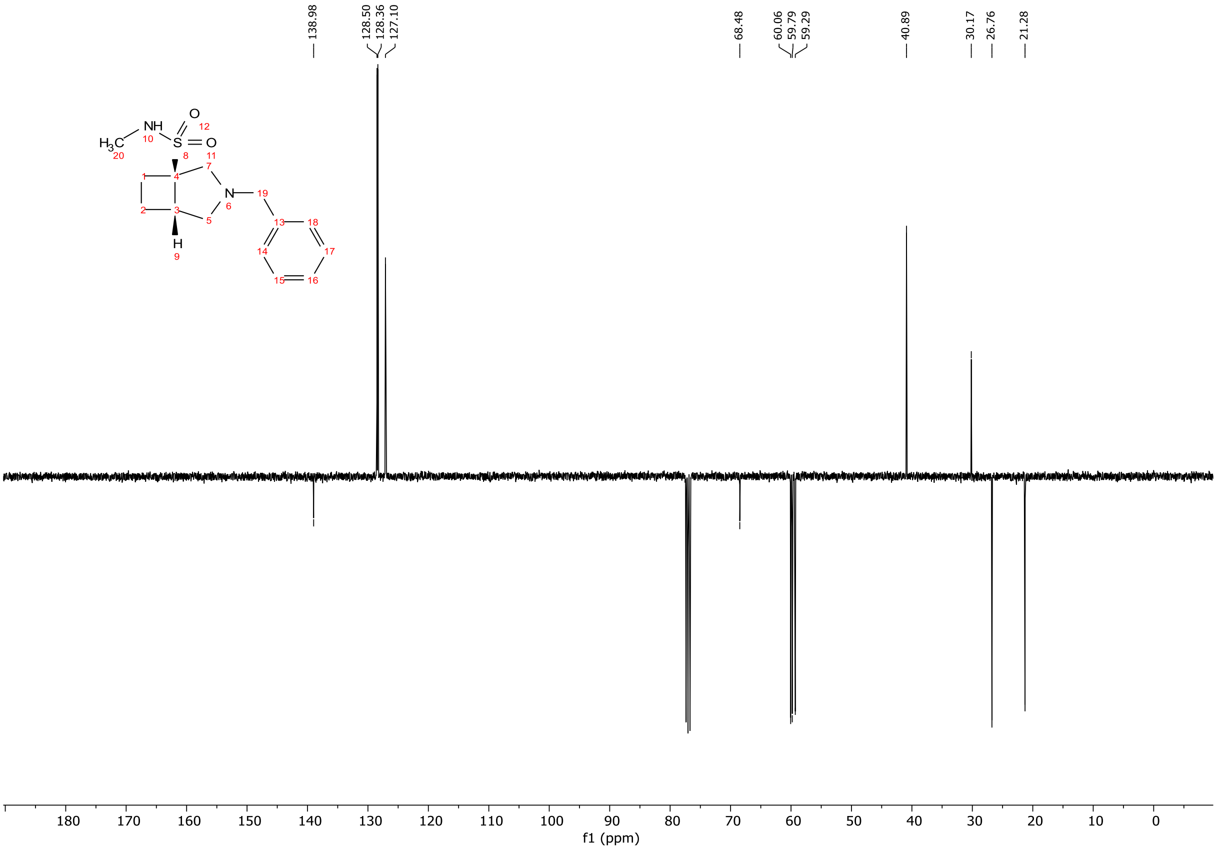

***Figure S18**. ^{13}C NMR spectrum (101 MHz, $CDCl_3$) of **13a**.*

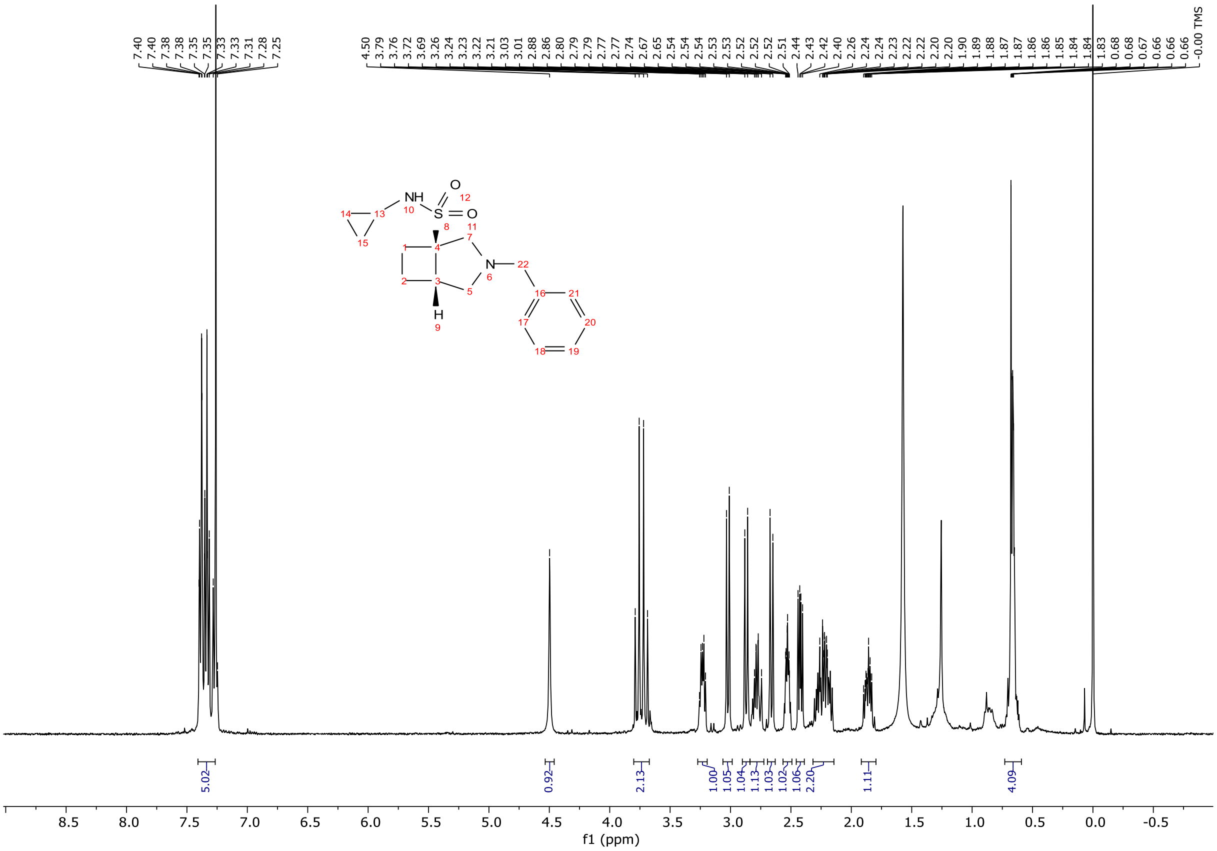

***Figure S19** ^{1}H NMR spectrum (400 MHz, $CDCl_3$) of **13b**.*

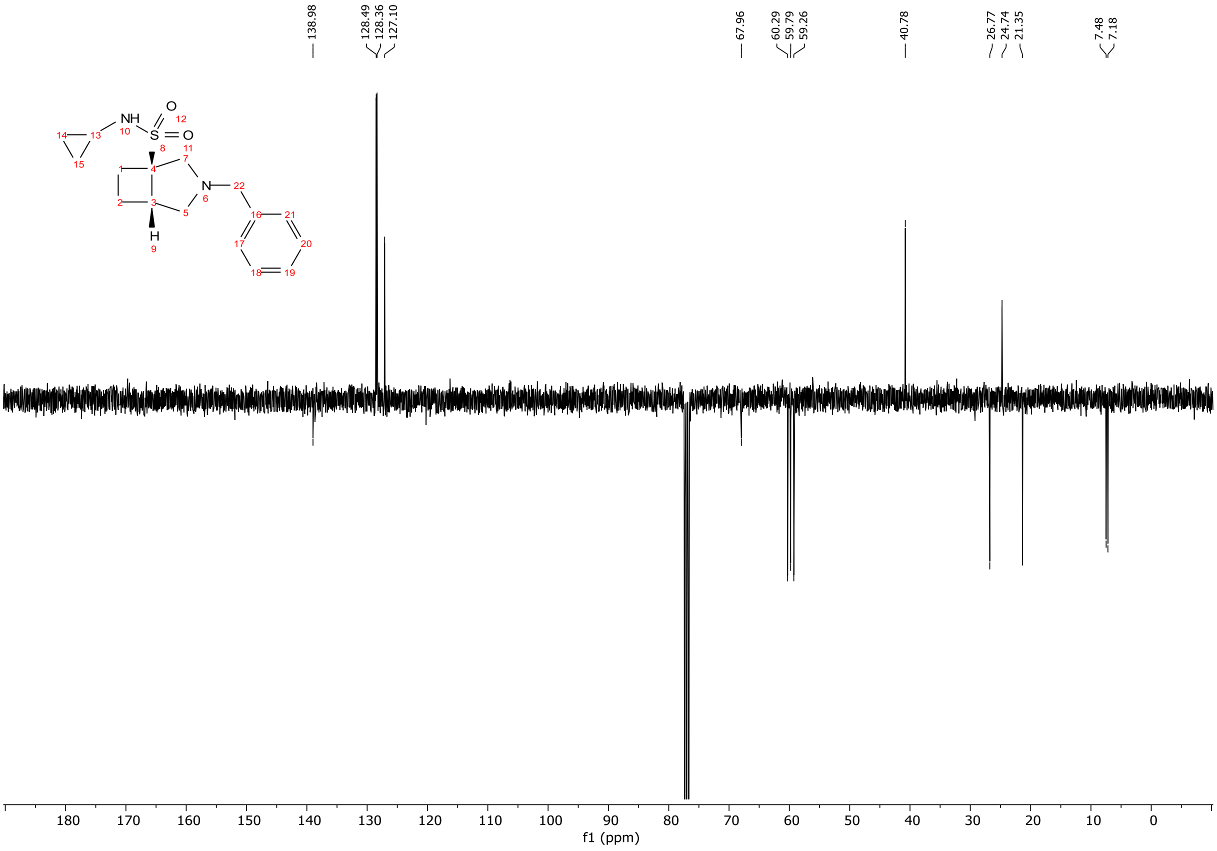

***Figure S20** ^{13}C NMR spectrum (101 MHz, $CDCl_3$) of **13b**.*

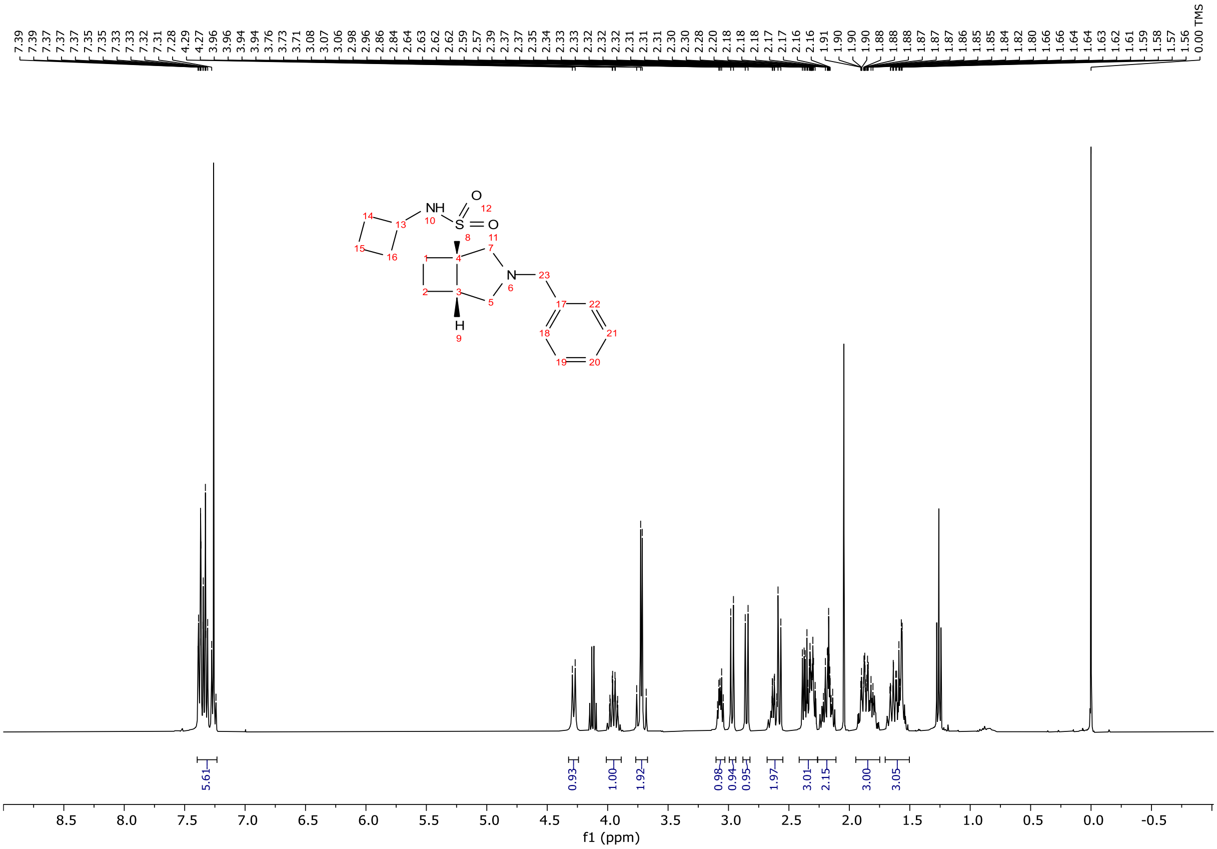

Figure S21 *^{1}H NMR spectrum (400 MHz, $CDCl_3$) of **13c**.*

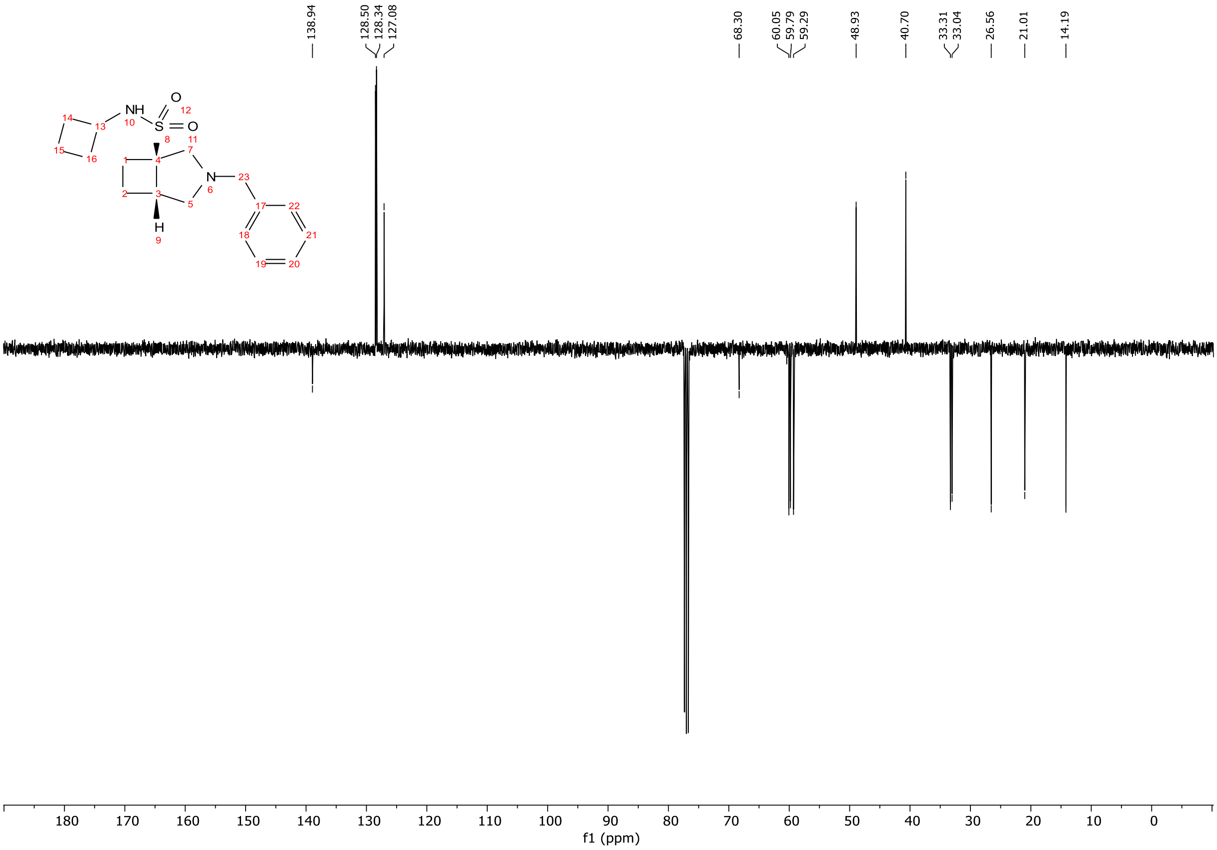

Figure S22 *^{13}C NMR spectrum (101 MHz, $CDCl_3$) of **13c**.*

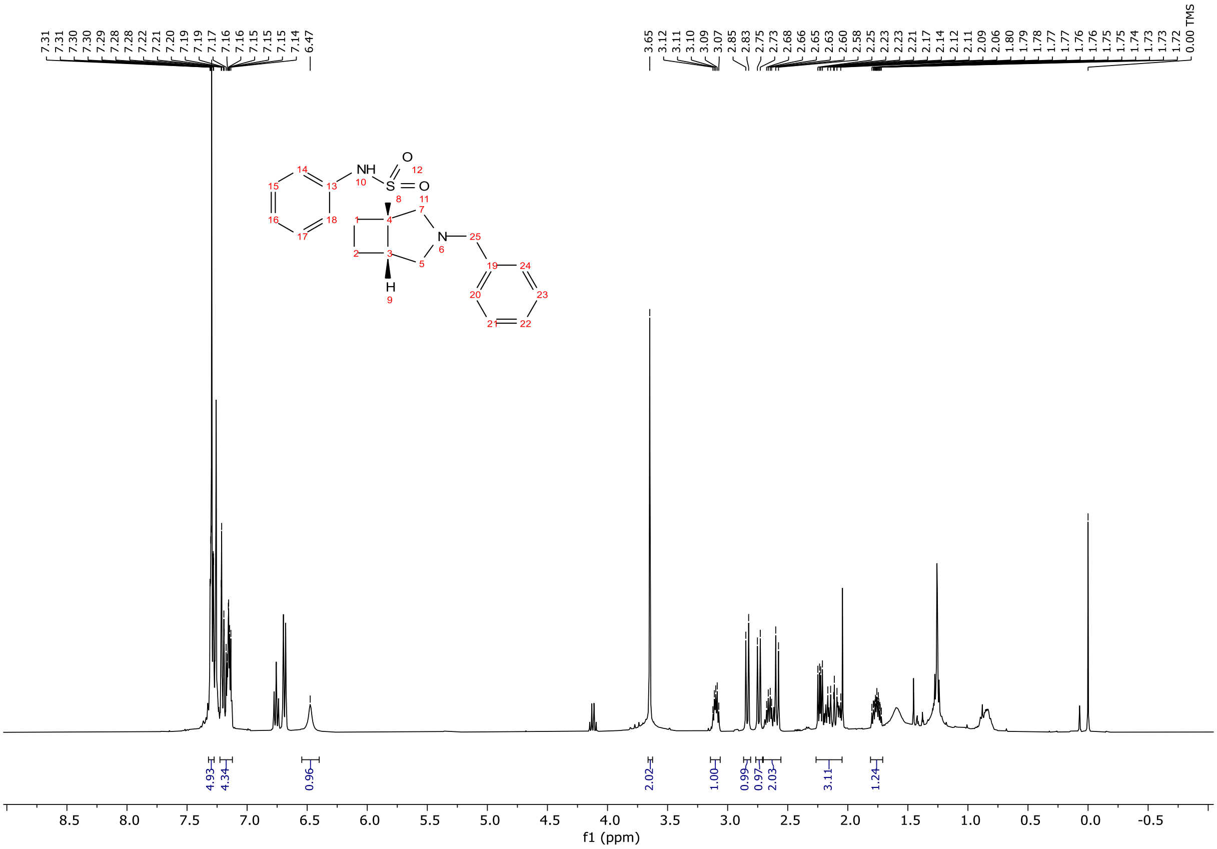

Figure S23 *^{1}H NMR spectrum (400 MHz, $CDCl_3$) of* ***13d****.*

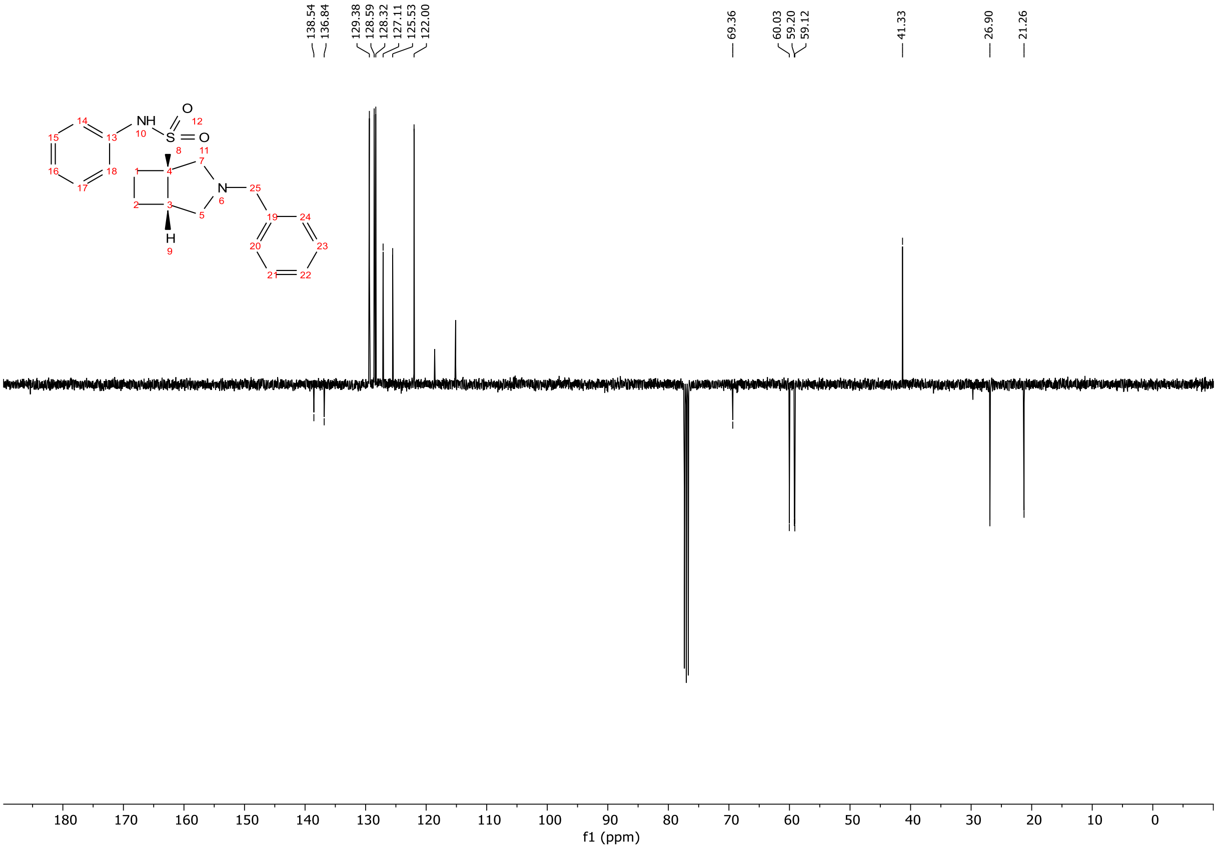

Figure S24 *^{13}C NMR spectrum (101 MHz, $CDCl_3$) of* ***13d****.*

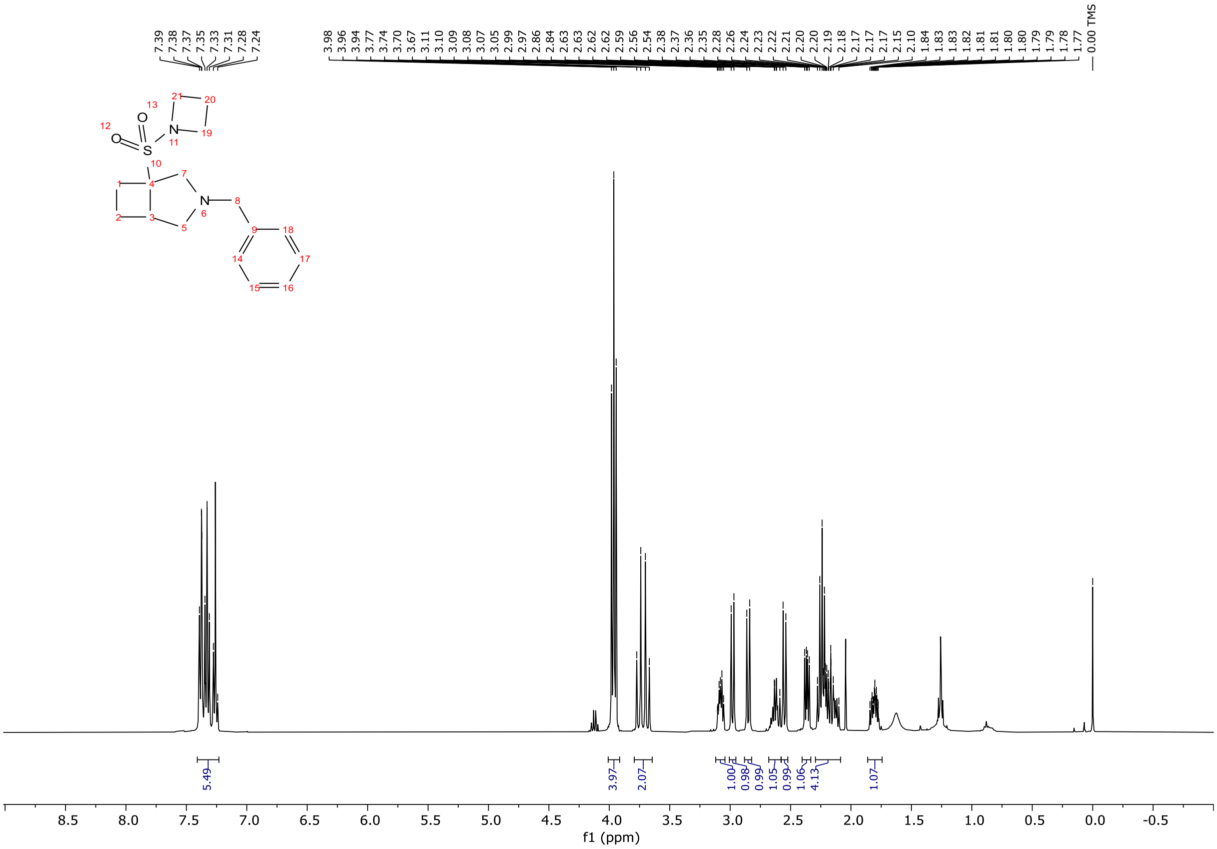

Figure S25 *^{1}H NMR spectrum (400 MHz, $CDCl_3$) of* ***13e****.*

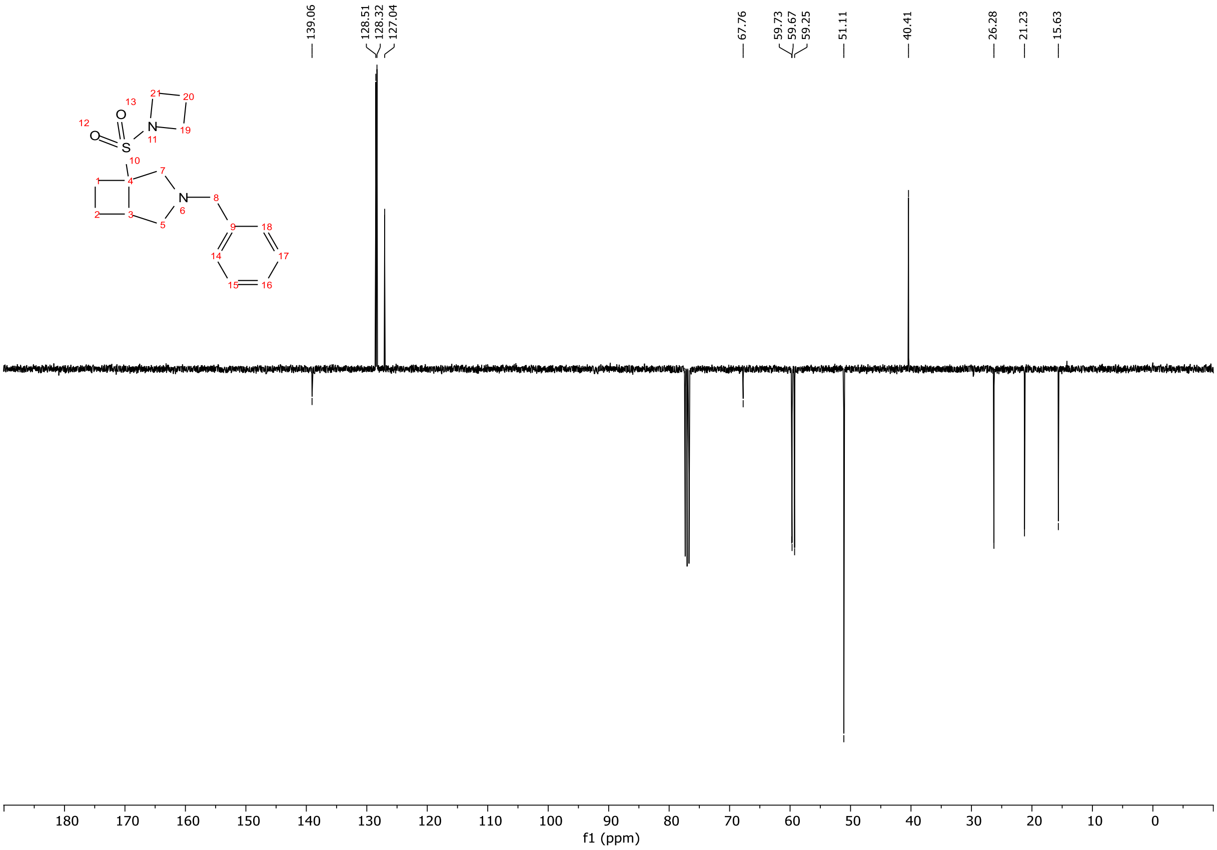

Figure S26 *^{13}C NMR spectrum (101 MHz, $CDCl_3$) of* ***13e****.*

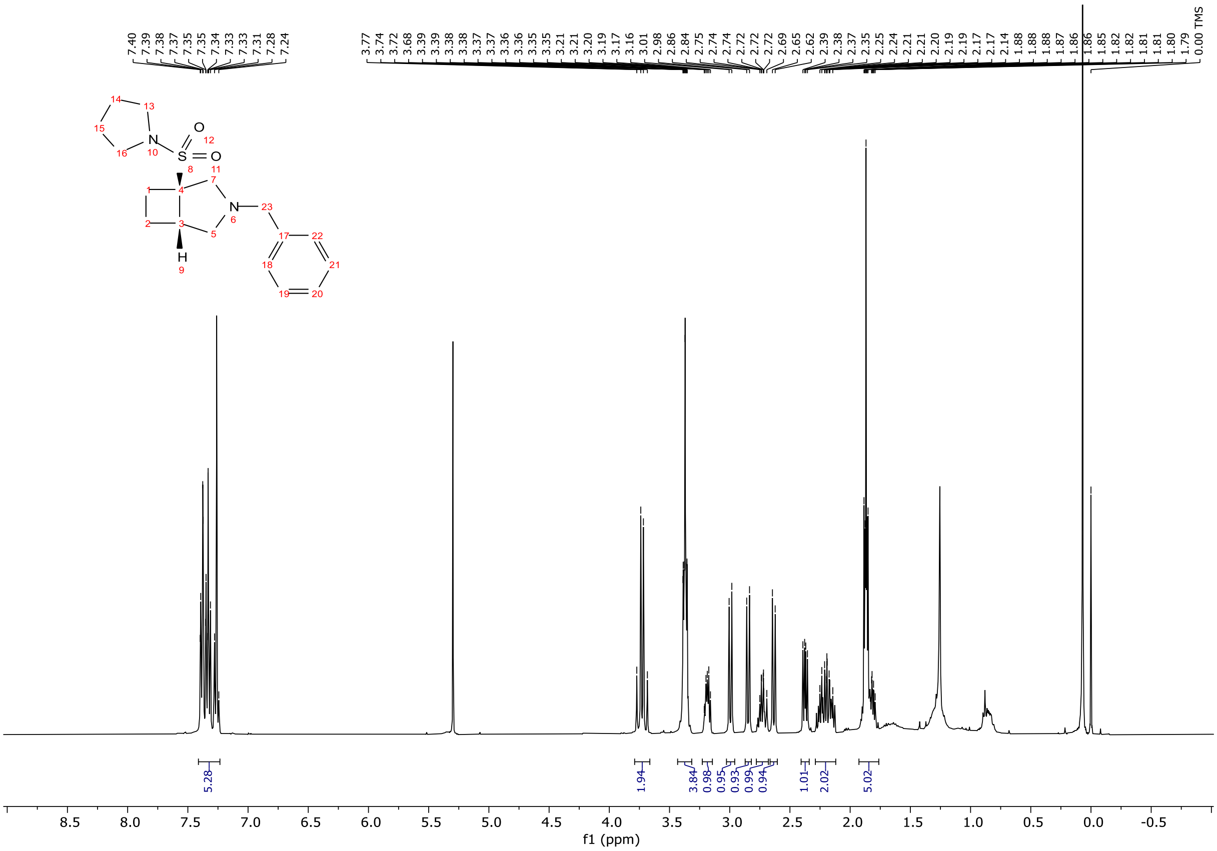

Figure S27 *^{1}H NMR spectrum (400 MHz, $CDCl_3$) of* ***13f***.

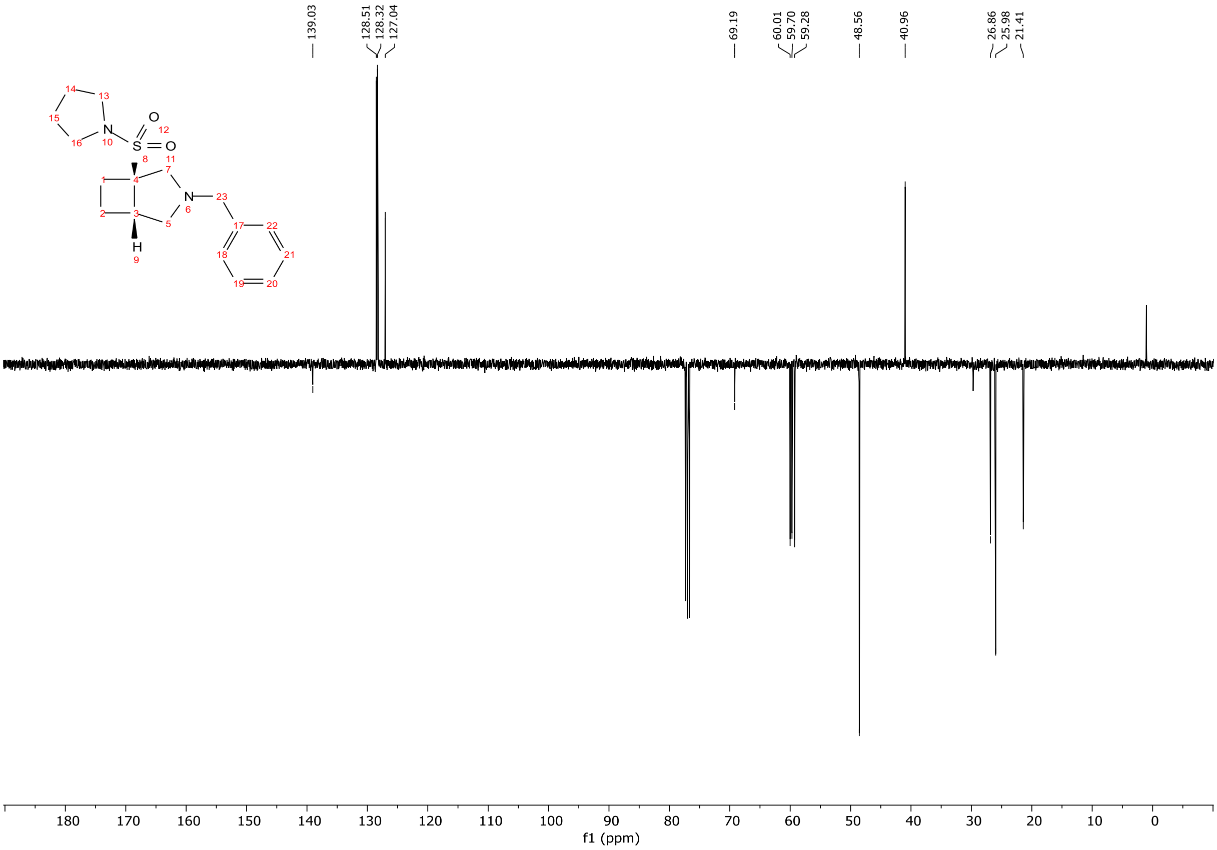

Figure S28 *^{13}C NMR spectrum (101 MHz, $CDCl_3$) of* ***13f***.

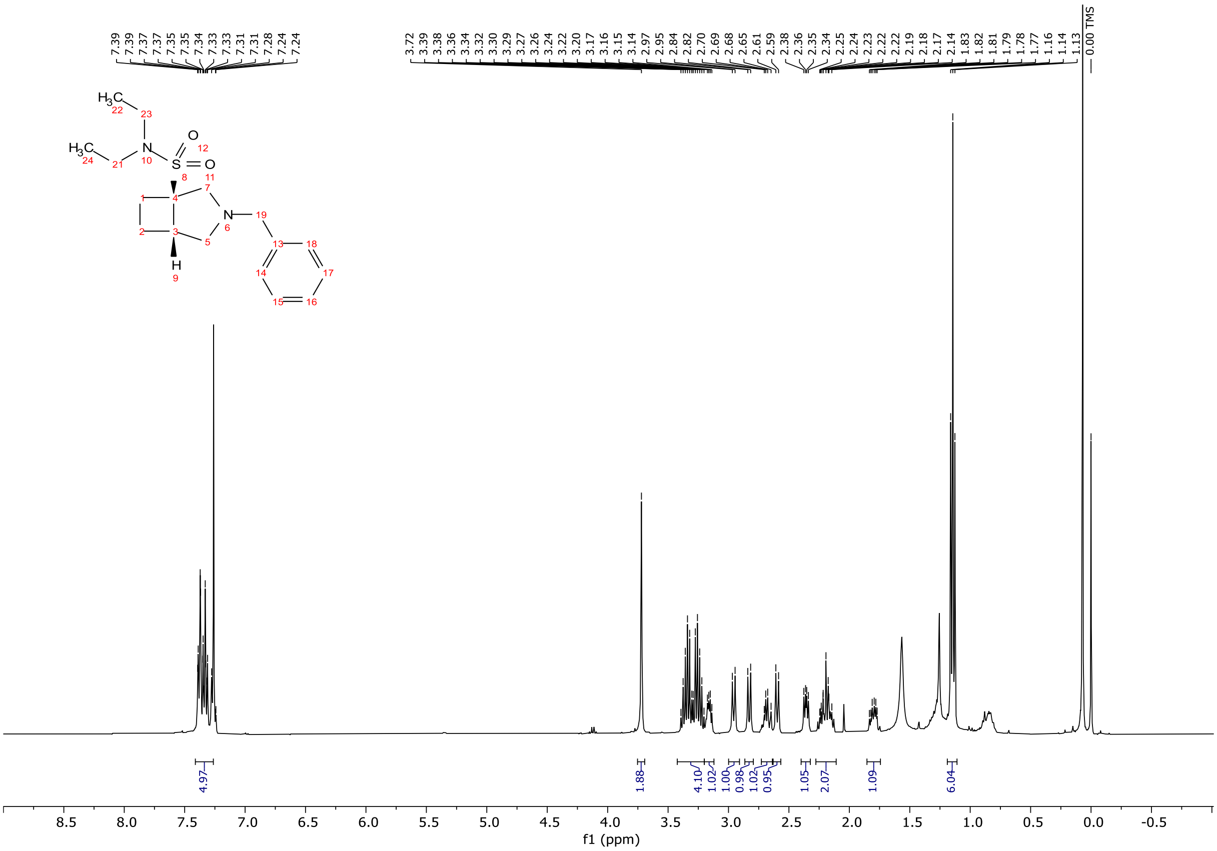

Figure S29 ^{1}H NMR spectrum (400 MHz, $CDCl_3$) of **13g**.

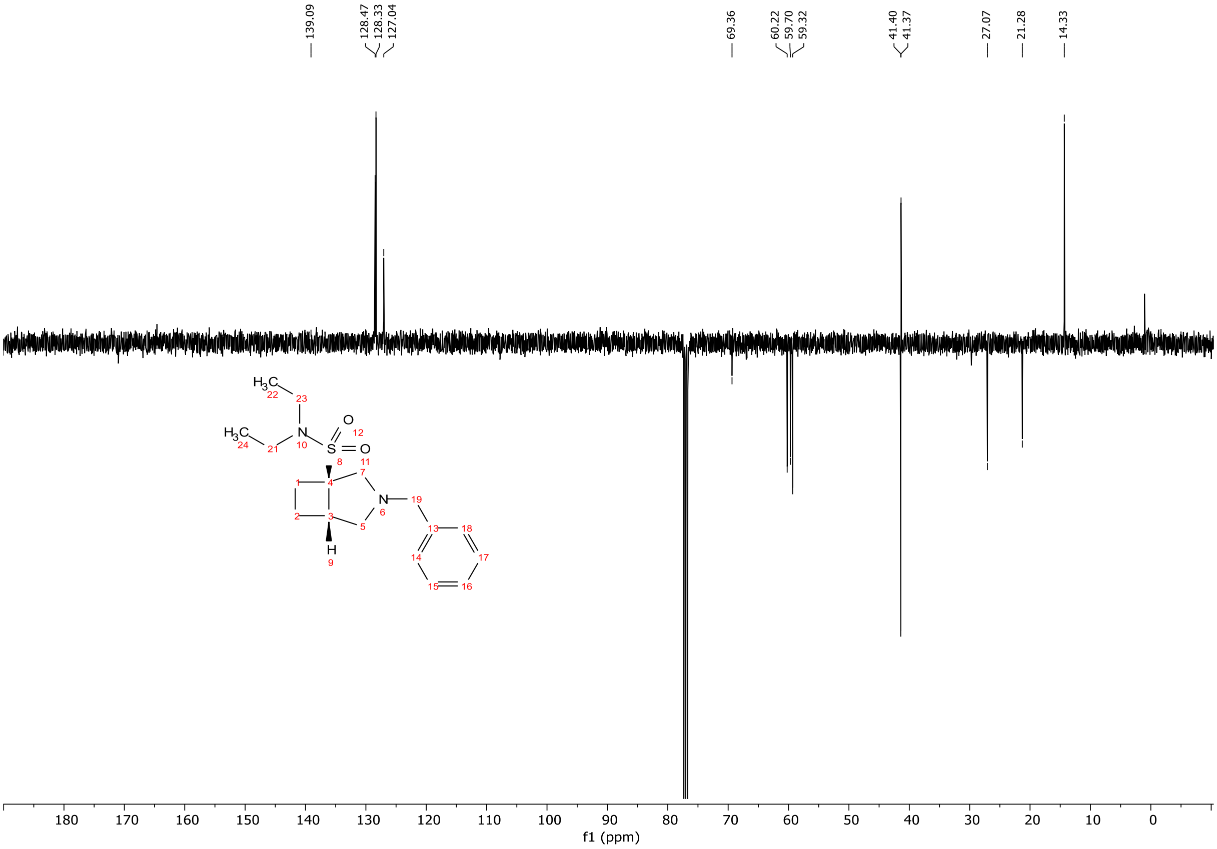

Figure S30 ^{13}C NMR spectrum (101 MHz, $CDCl_3$) of **13g**.

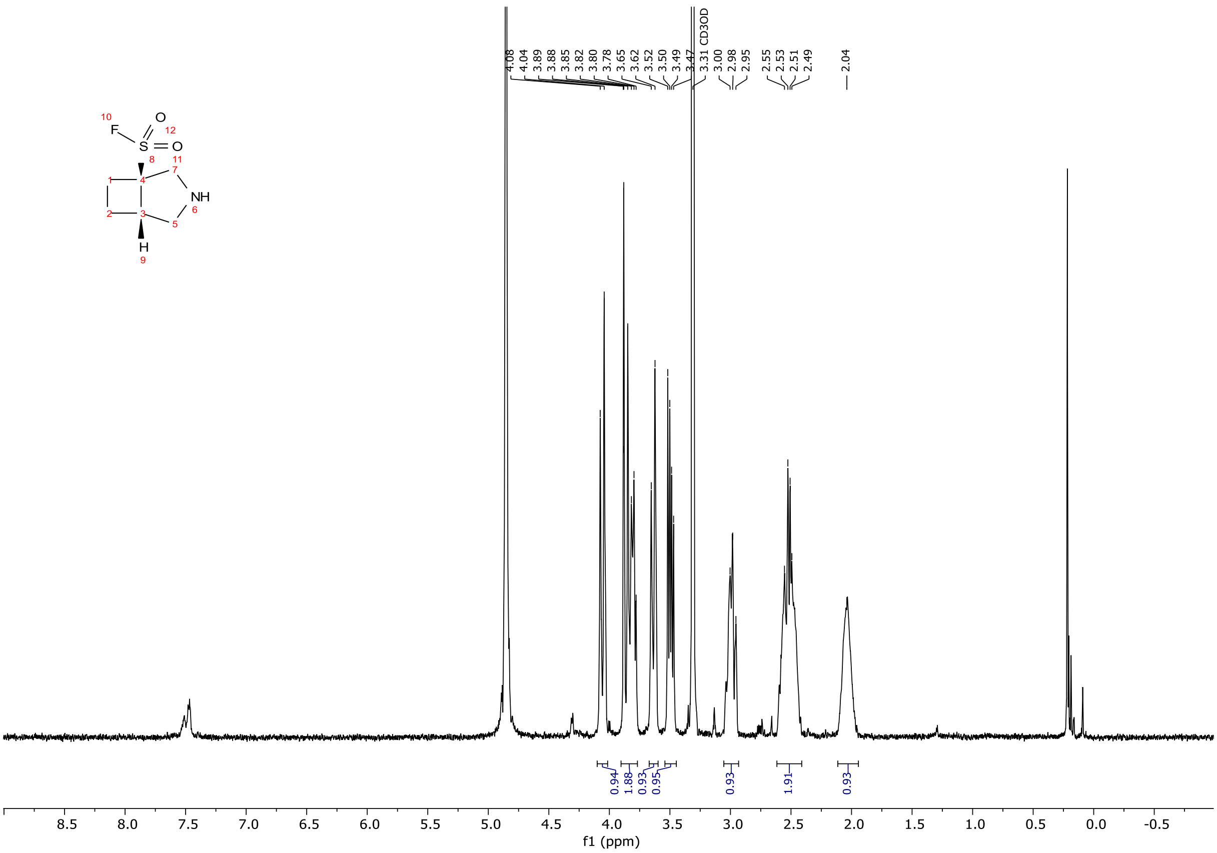

Figure S31 *^{1}H NMR spectrum (400 MHz, CD_3OD) of **12**.*

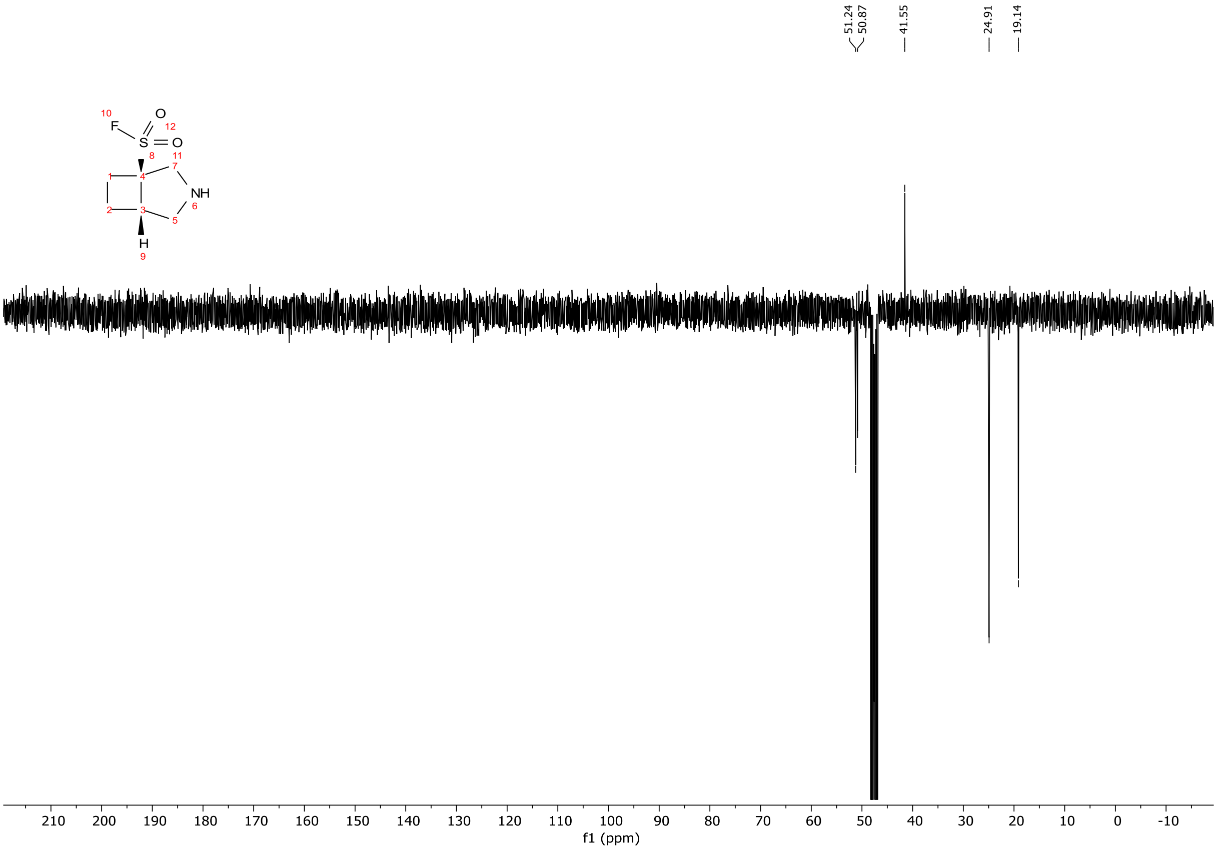

Figure S32 *^{13}C NMR spectrum (101 MHz, CD_3OD) of **12**.*

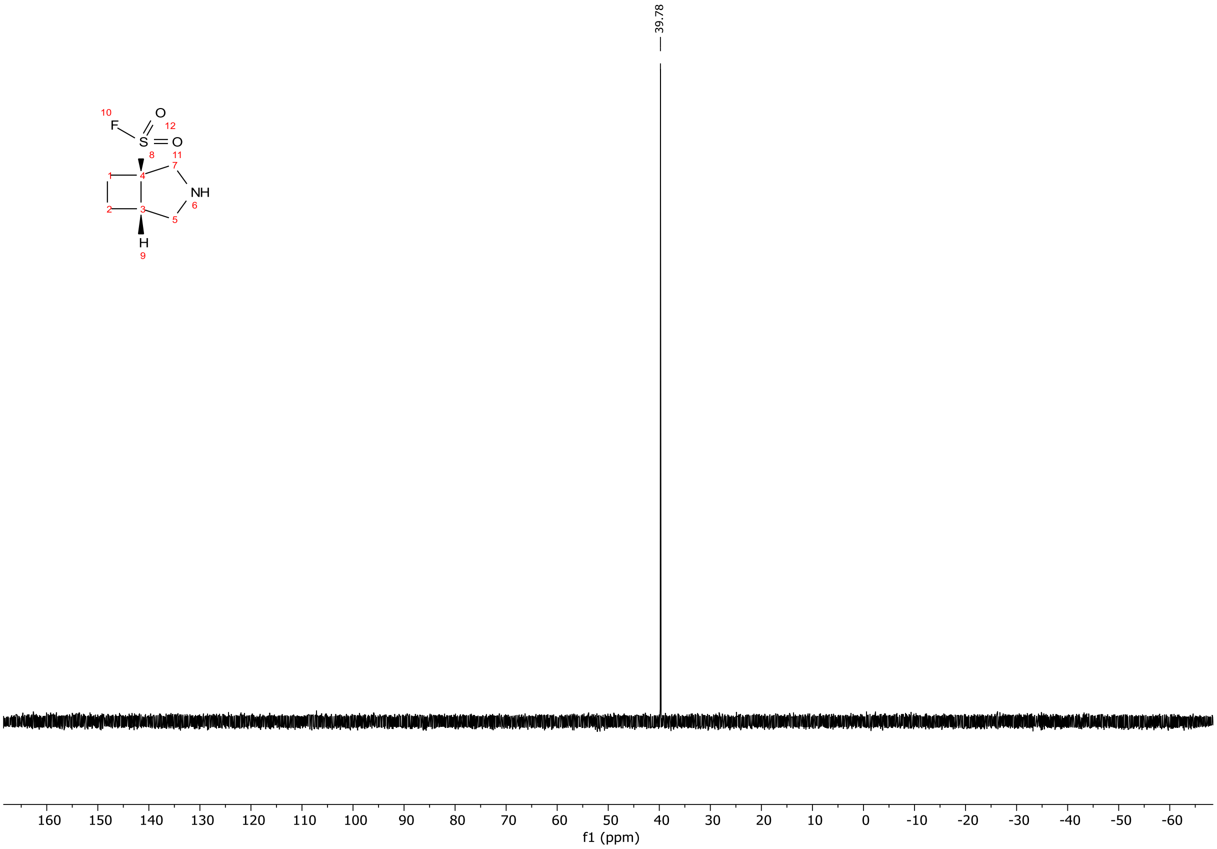

Figure S33 *^{19}F NMR spectrum (377 MHz, CD_3OD) of* ***12***.

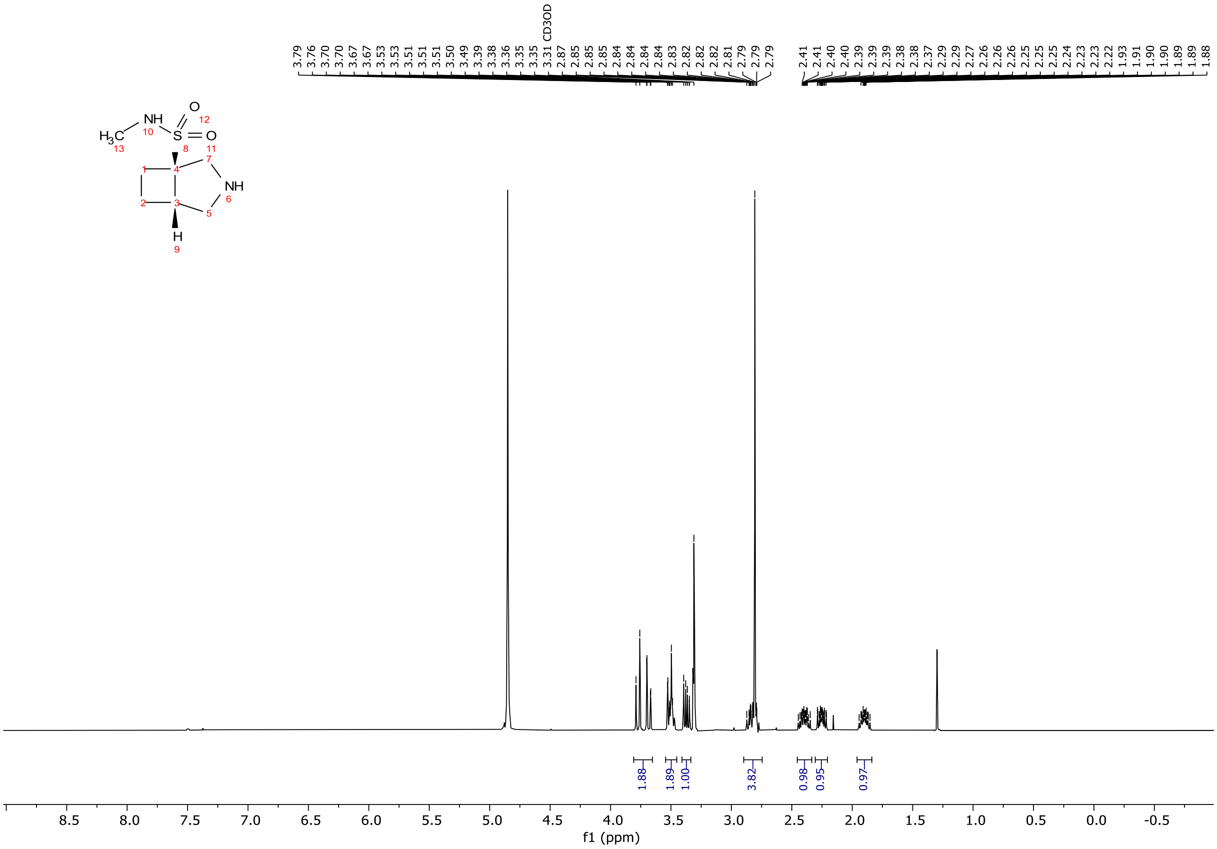

Figure S34 *^{1}H NMR spectrum (400 MHz, CD_3OD) of* ***14a***.

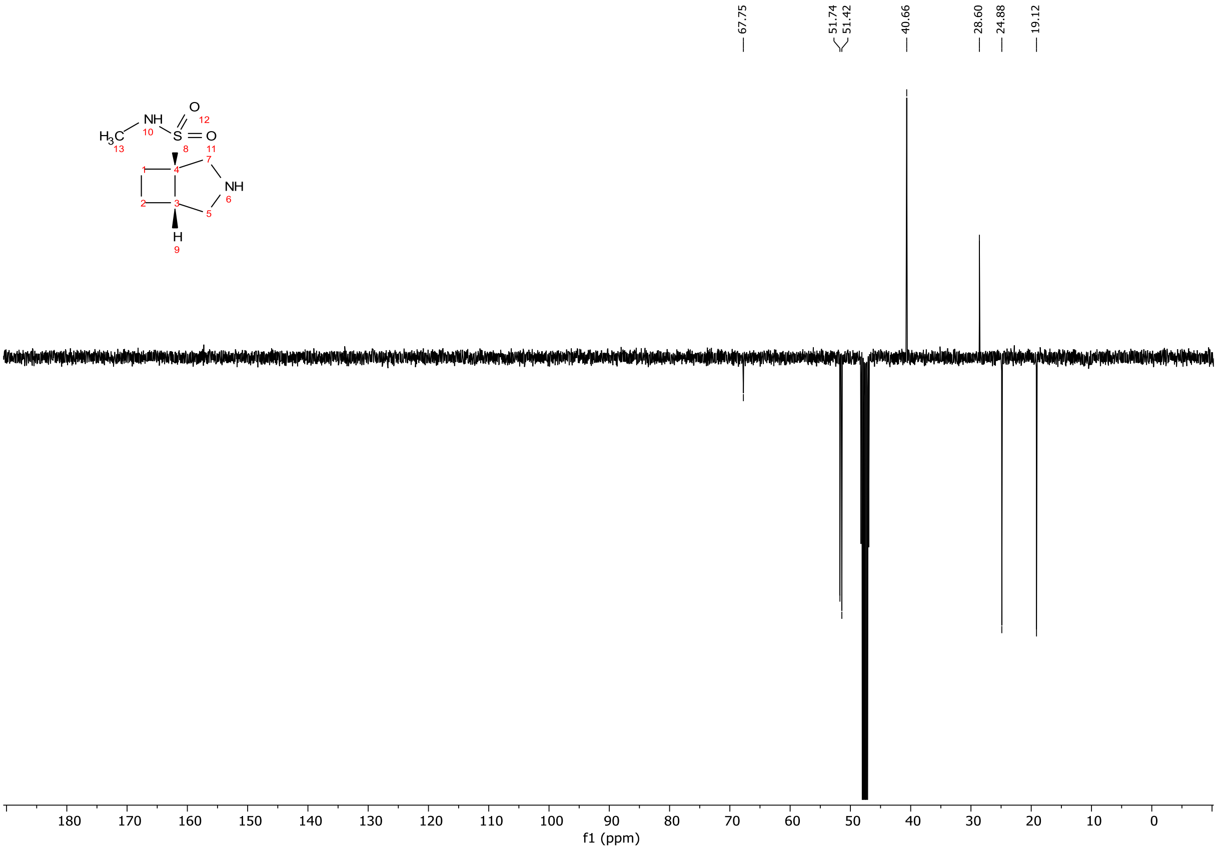

Figure S35 *^{13}C NMR spectrum (101 MHz, CD_3OD) of* ***14a***.

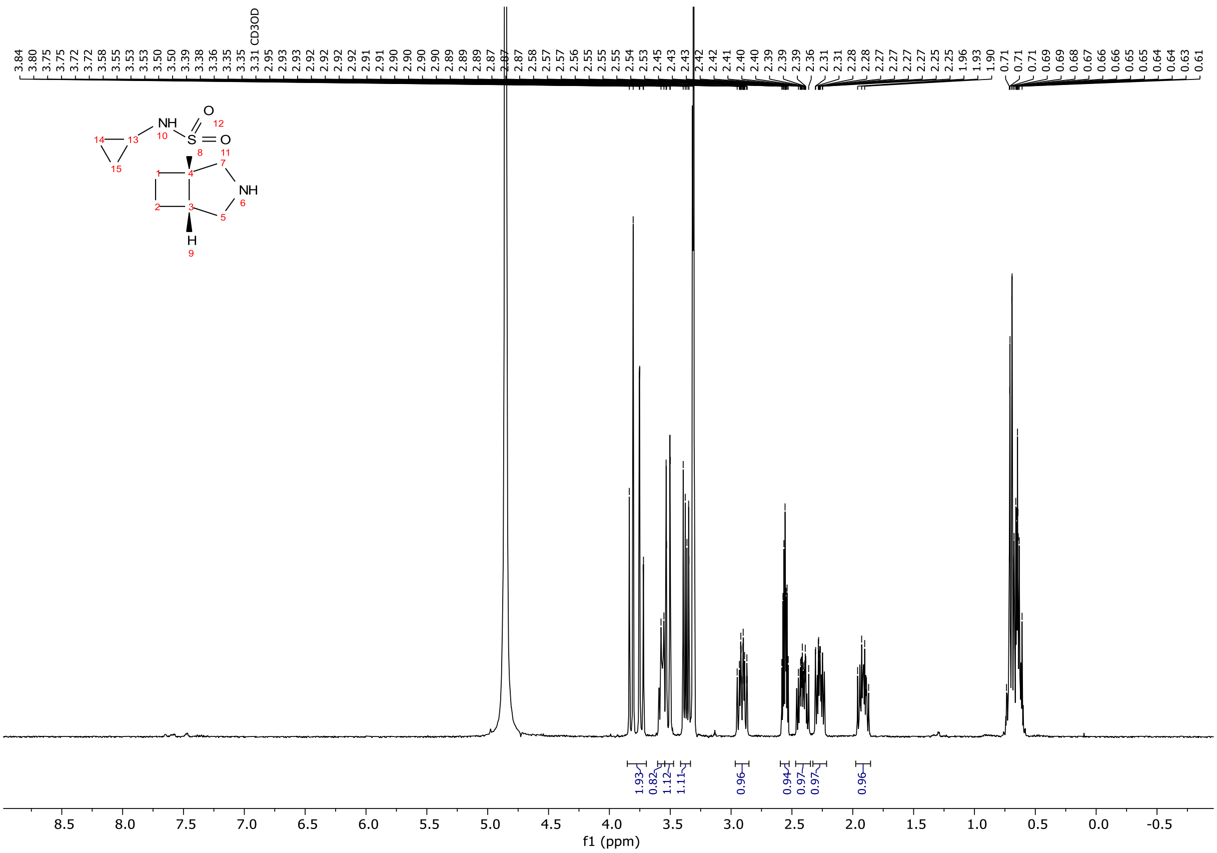

Figure S36 *^{1}H NMR spectrum (400 MHz, CD_3OD) of **14b**.*

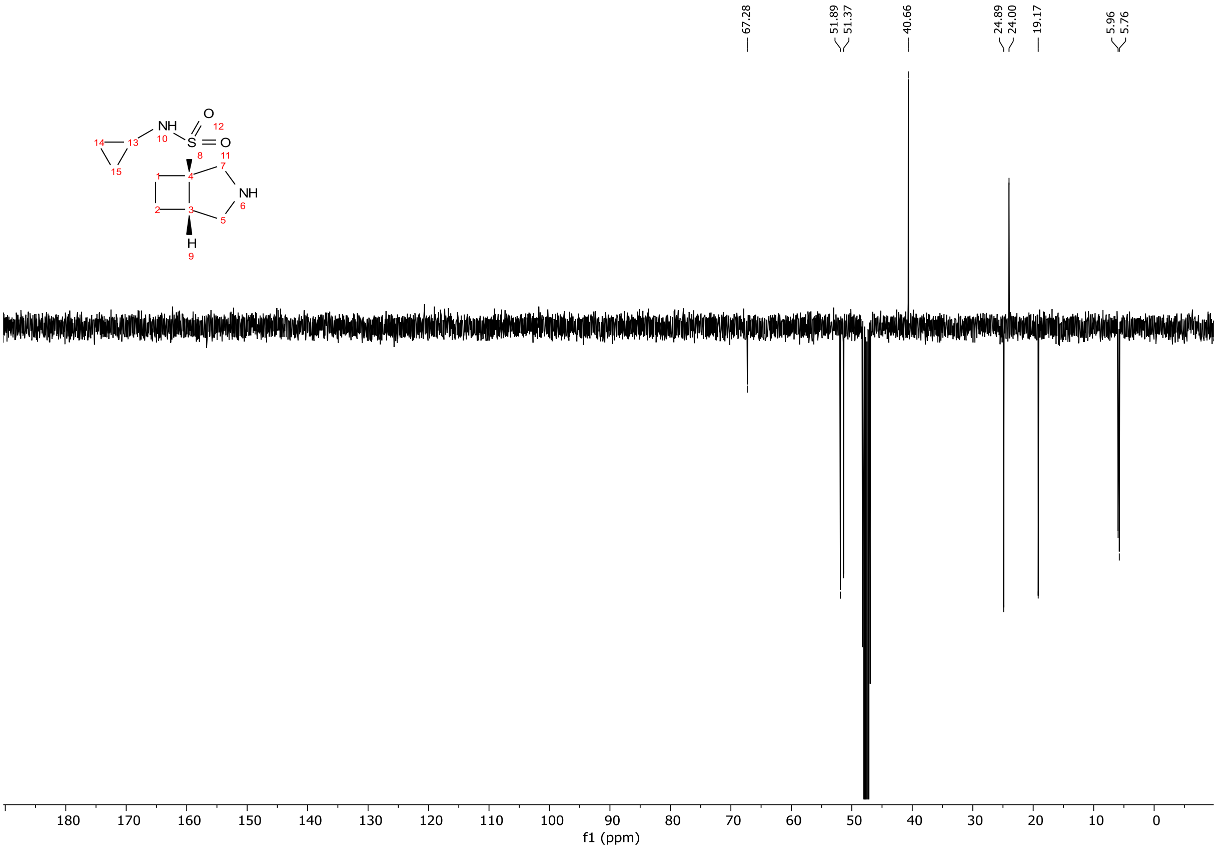

Figure S37 *^{13}C NMR spectrum (101 MHz, CD_3OD) of **14b**.*

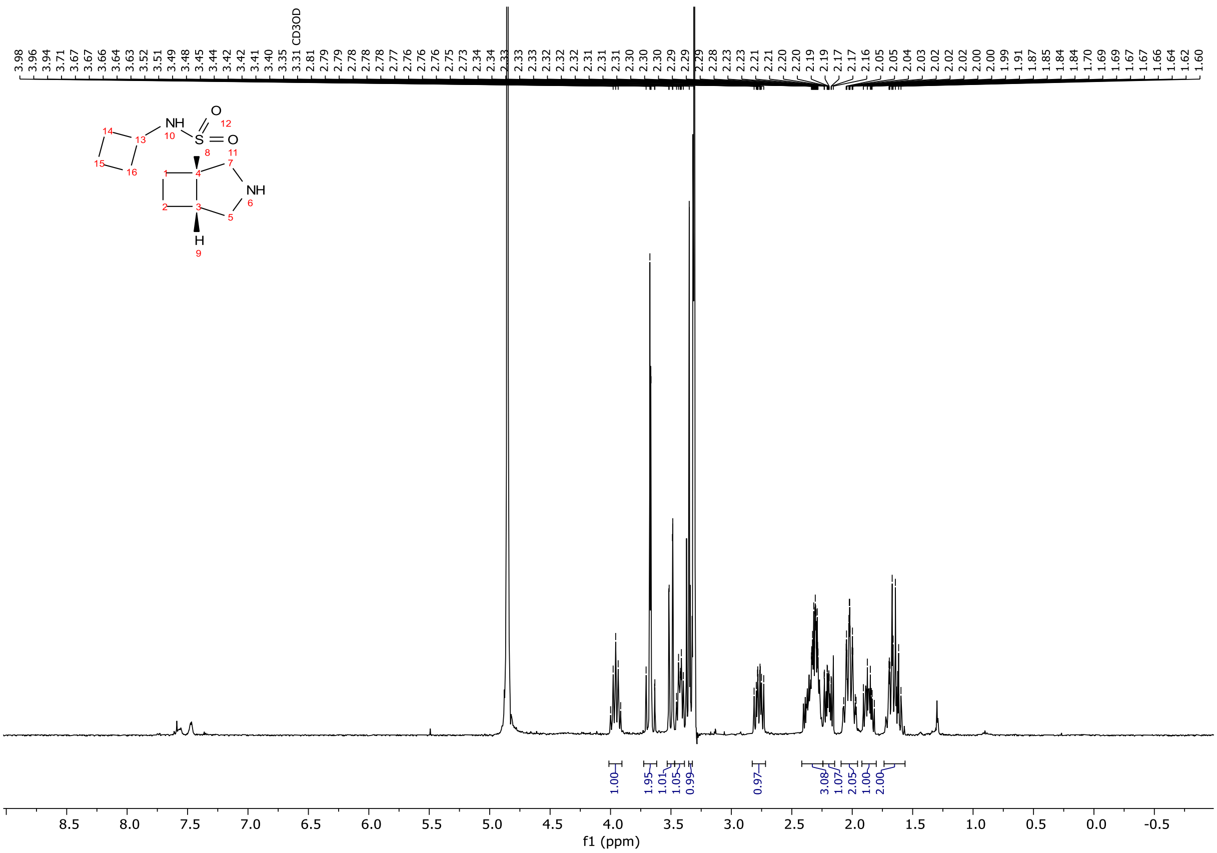

Figure S38 *^{1}H NMR spectrum (400 MHz, CD_3OD) of* ***14c***.

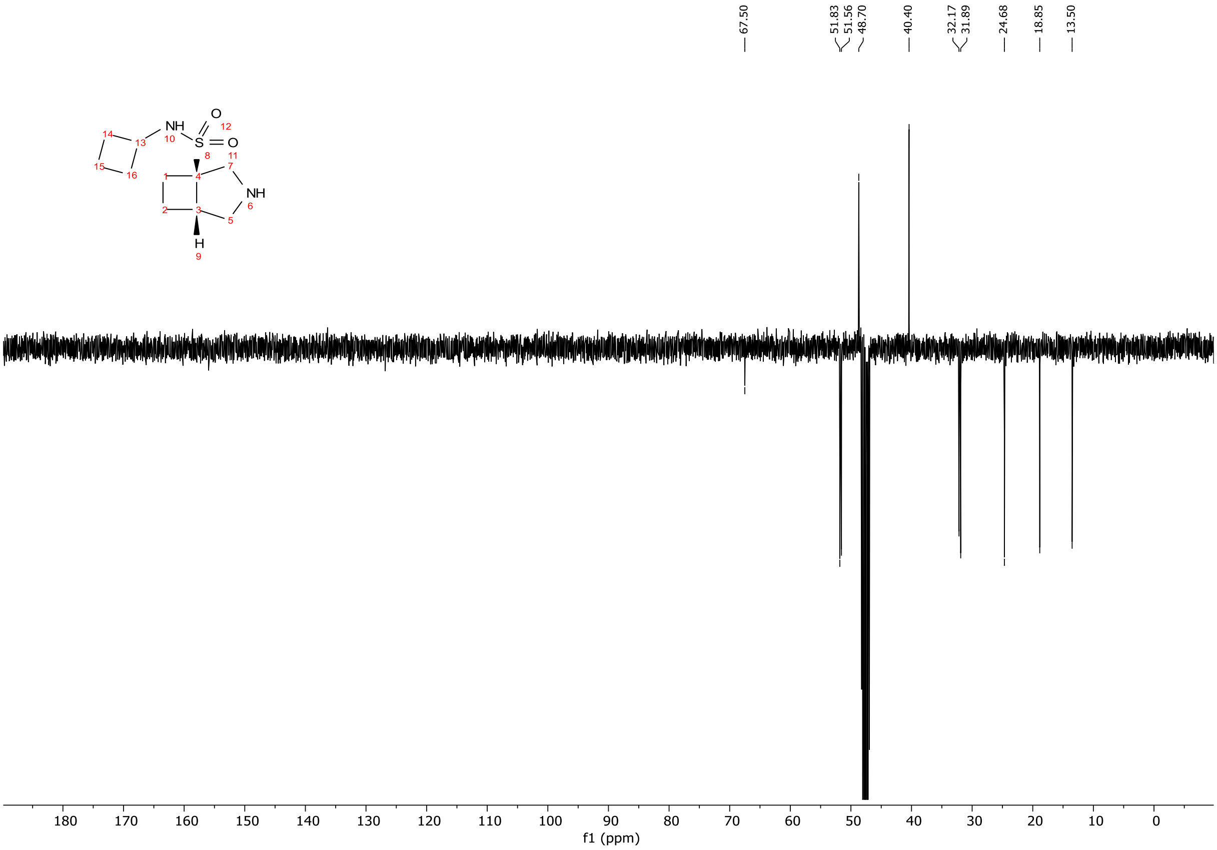

Figure S39 *^{13}C NMR spectrum (101 MHz, CD_3OD) of* ***14c***.

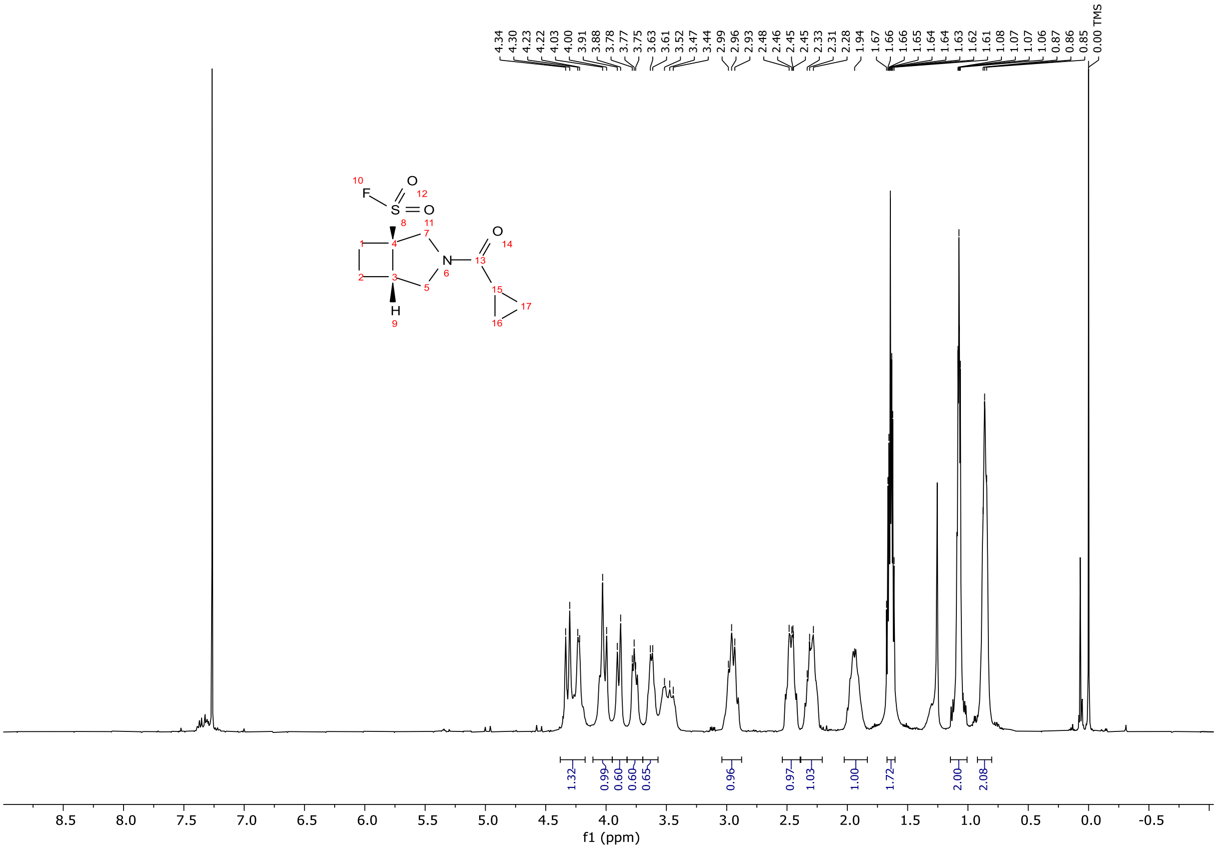

Figure S40 *^{1}H NMR spectrum (400 MHz, CDCl$_3$) of* ***1a***.

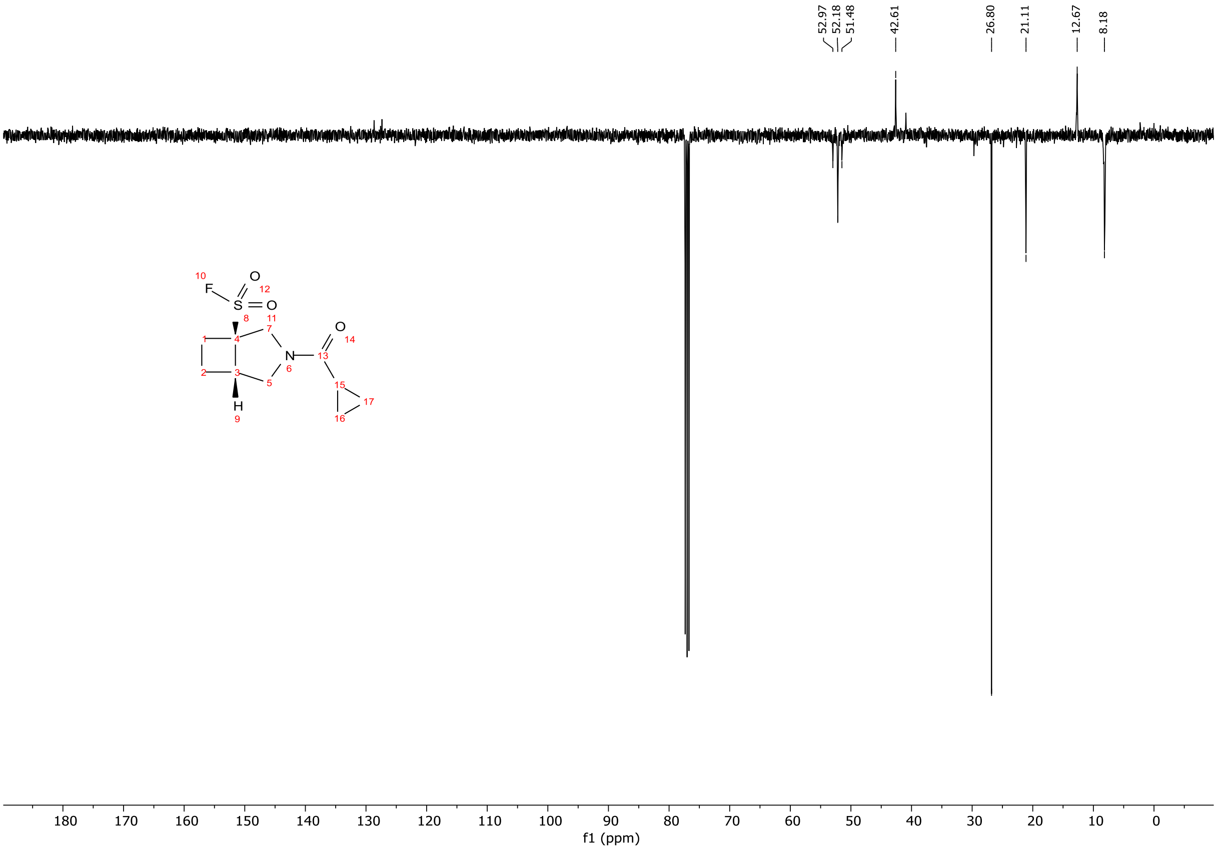

Figure S41 *^{13}C NMR spectrum (101 MHz, CDCl$_3$) of* ***1a***.

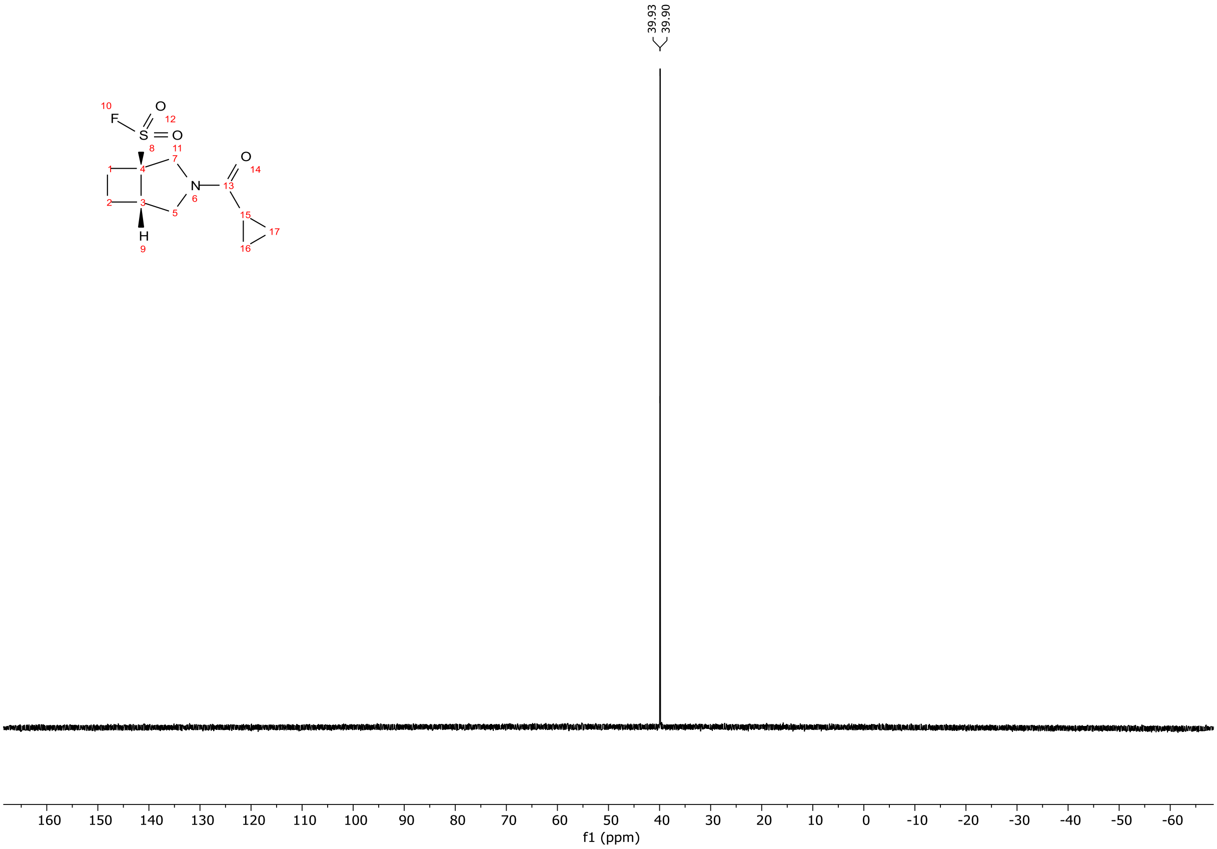

Figure S42 *^{19}F NMR spectrum (377 MHz, $CDCl_3$) of* ***1a***.

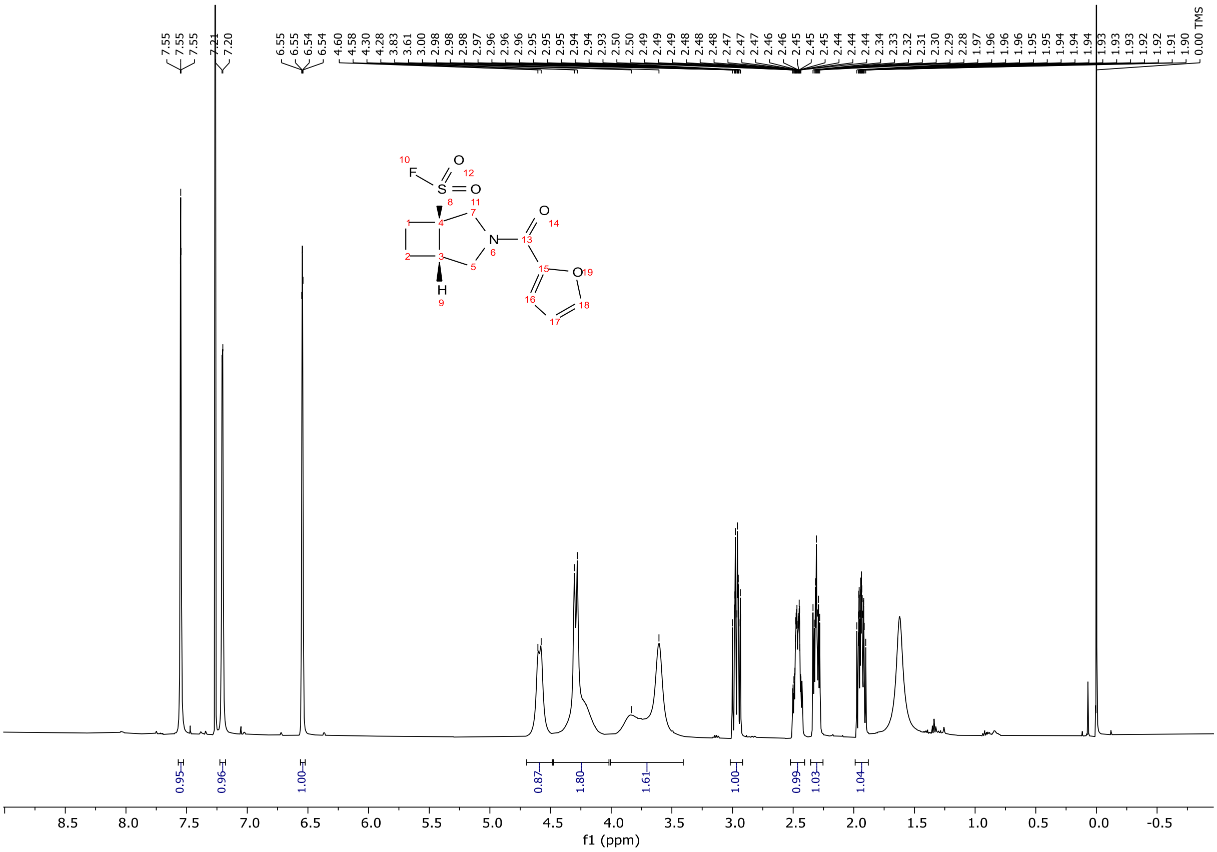

Figure S43 *^{1}H NMR spectrum (500 MHz, $CDCl_3$) of* ***1b***.

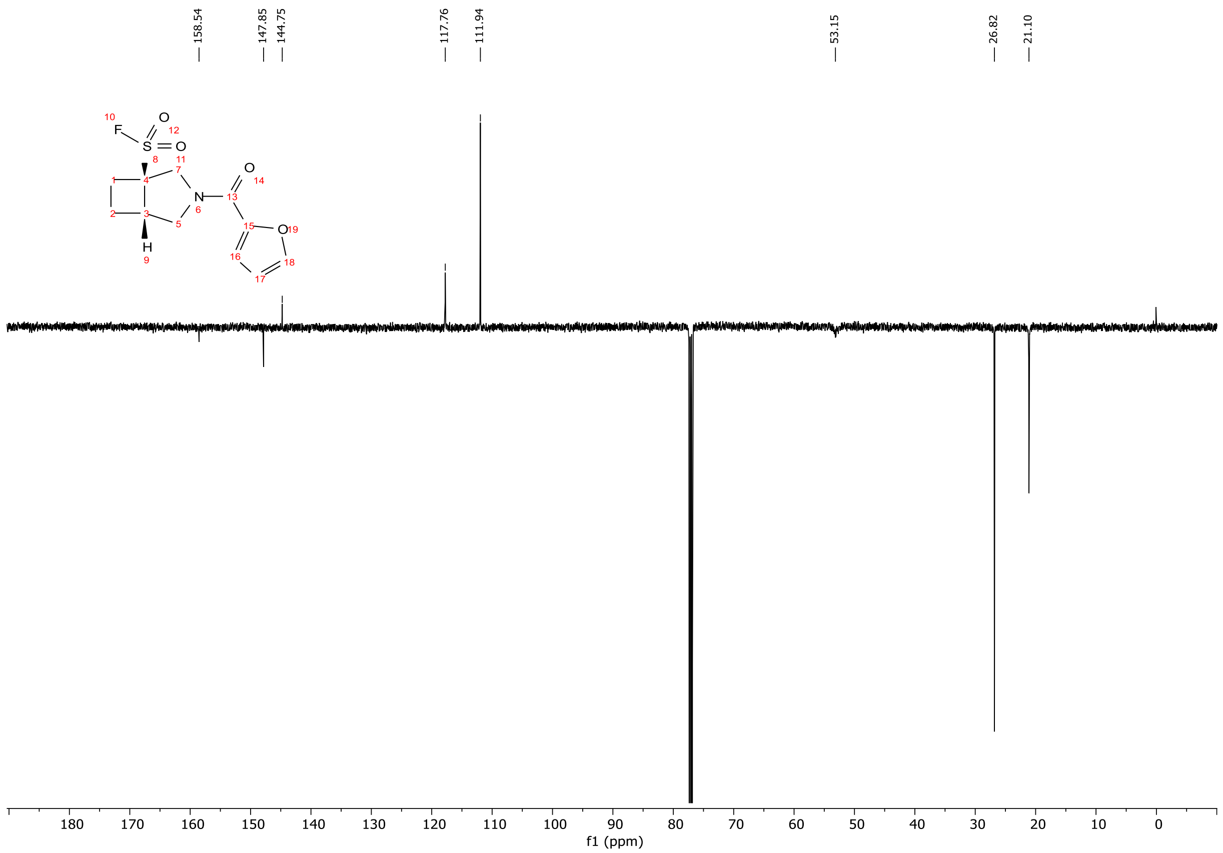

Figure S44 *^{13}C NMR spectrum (126 MHz, $CDCl_3$) of **1b**.*

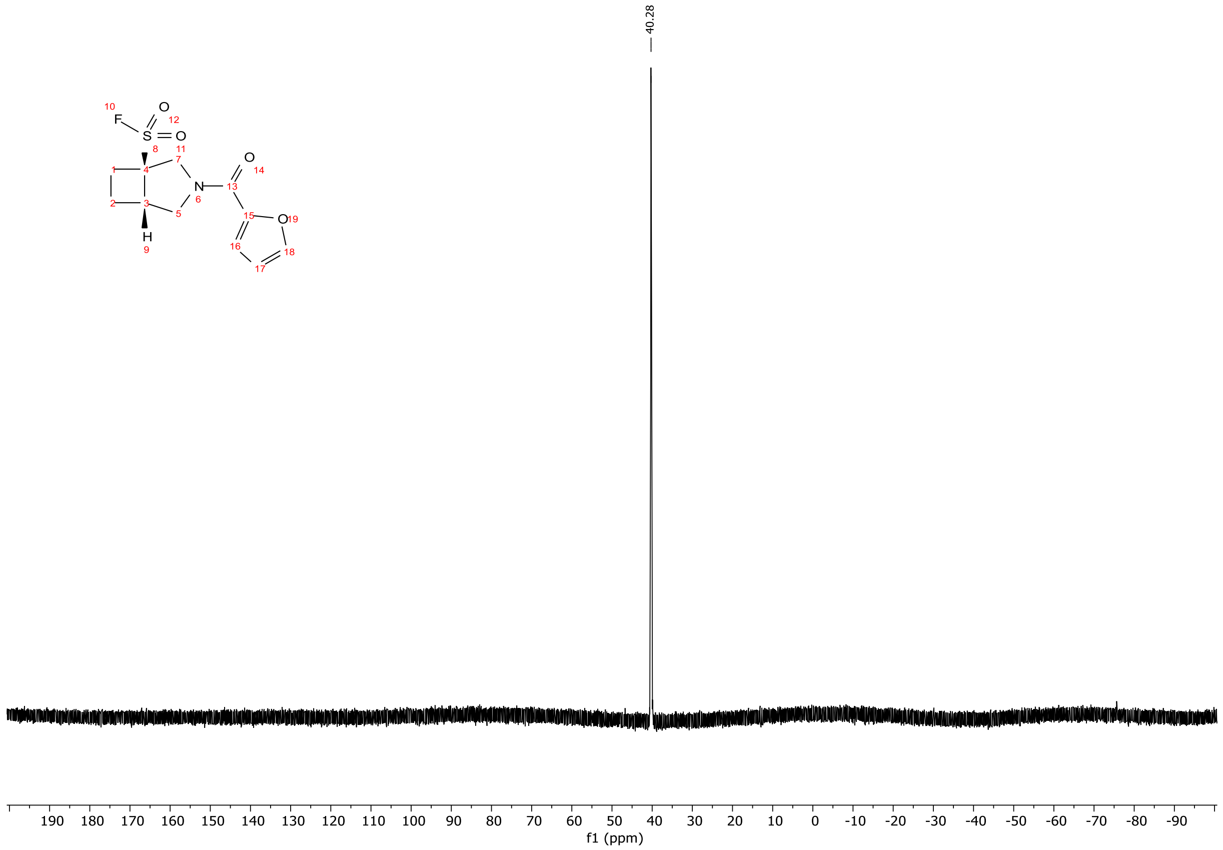

Figure S45 *^{19}F NMR spectrum (471 MHz, $CDCl_3$) of **1b**.*

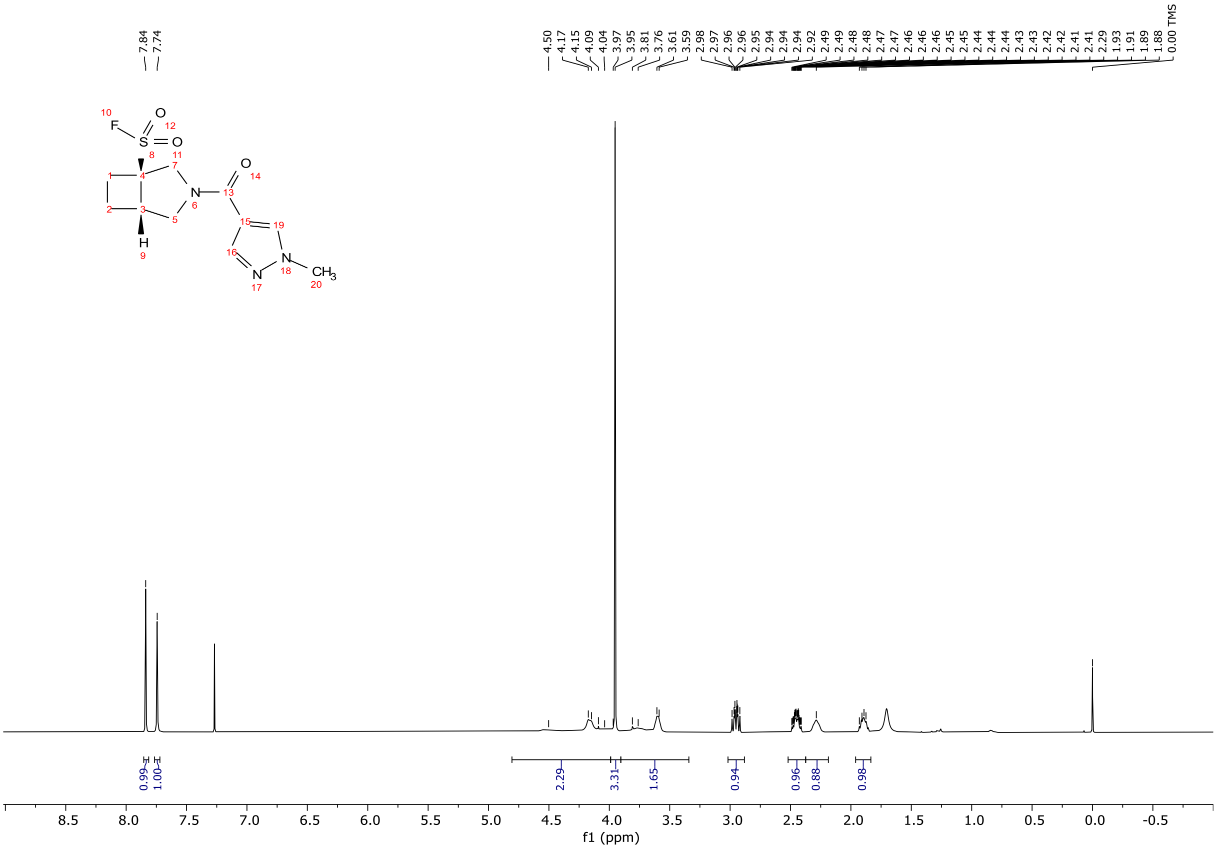

Figure S46 *^{1}H NMR spectrum (500 MHz, $CDCl_3$) of **1c**.*

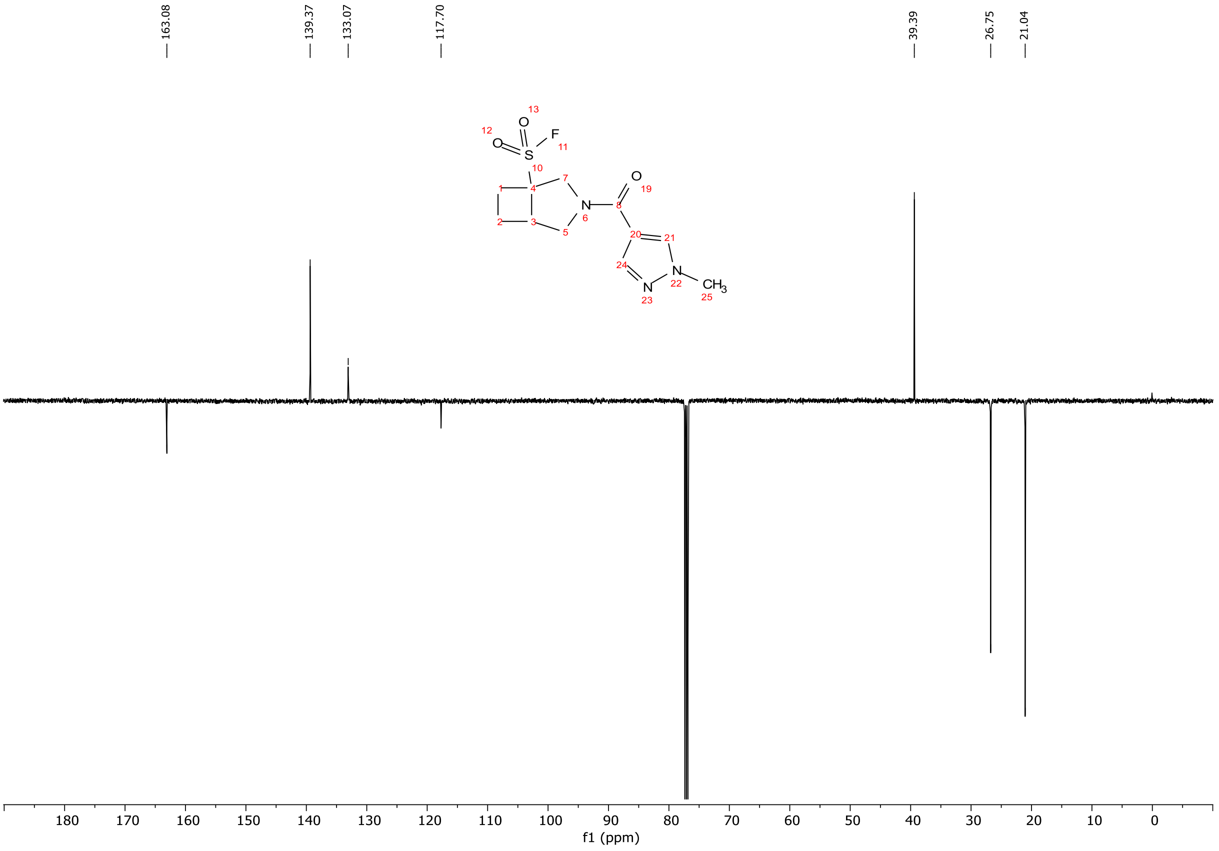

Figure S47 *^{13}C NMR spectrum (126 MHz, $CDCl_3$) of **1c**.*

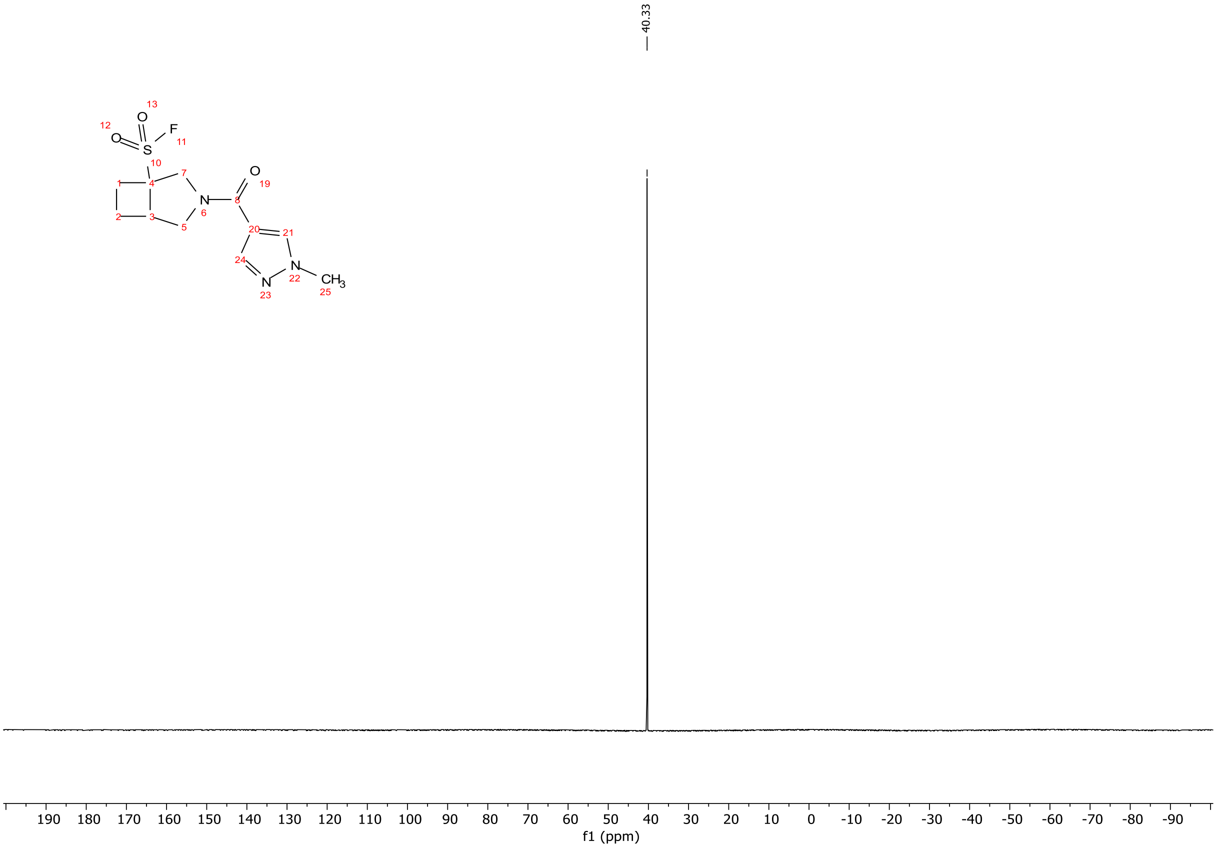

Figure S48 *^{19}F NMR spectrum (471 MHz, $CDCl_3$) of **1c**.*

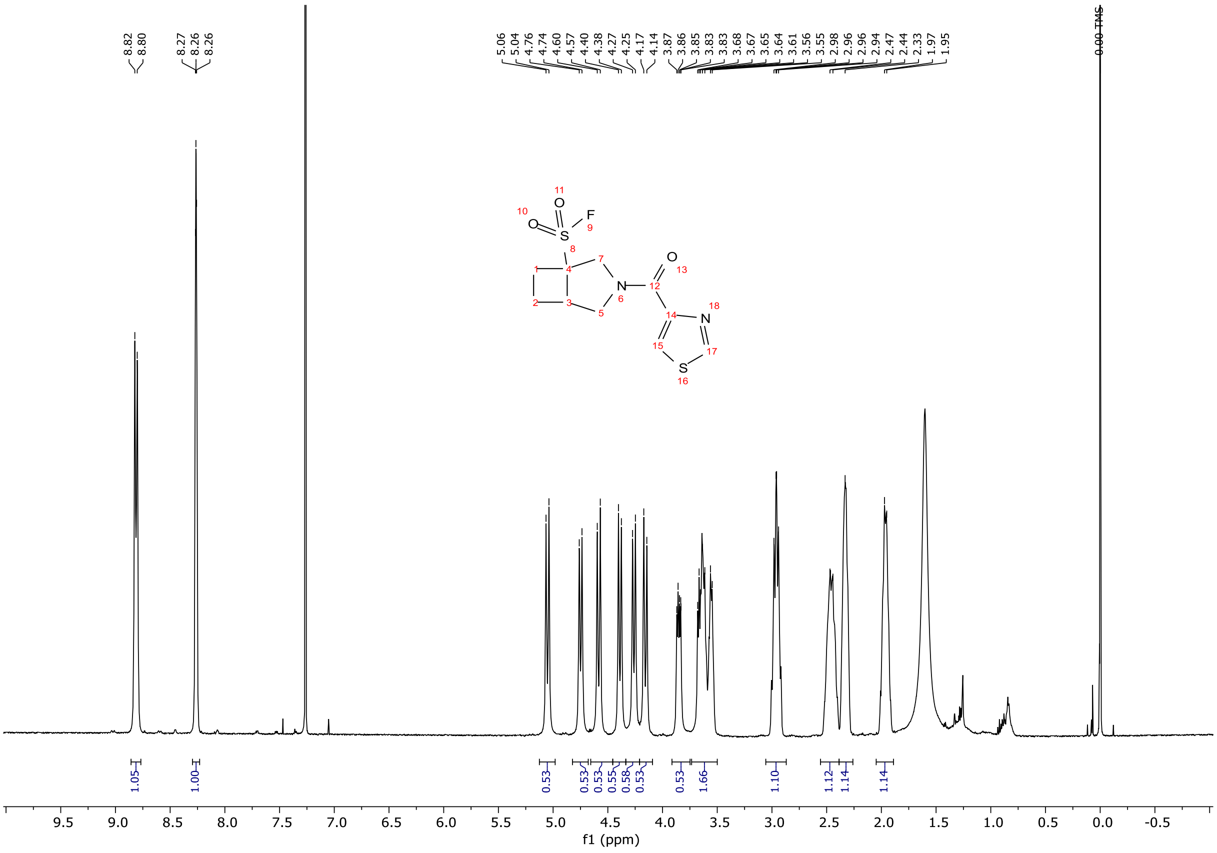

Figure S49 *^{1}H NMR spectrum (500 MHz, $CDCl_3$) of **1d**.*

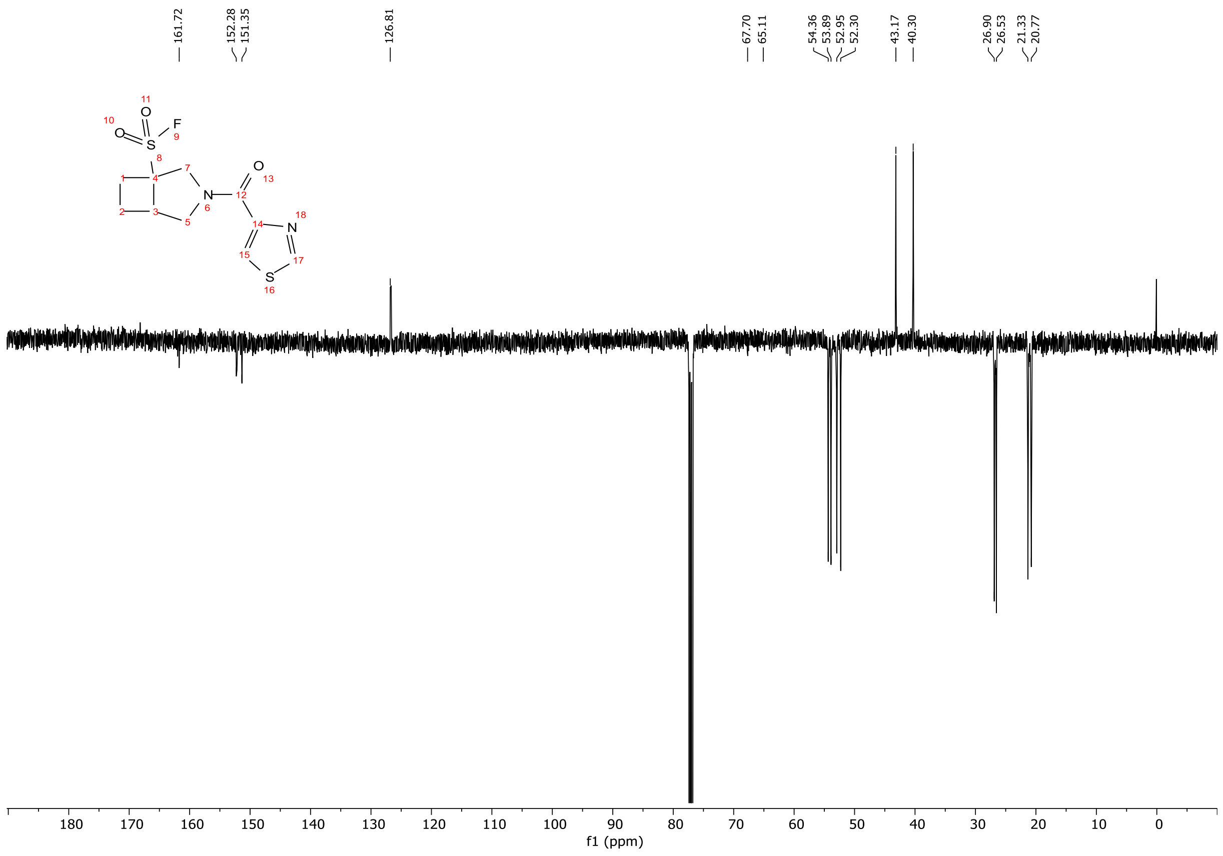

Figure S50 ^{13}C NMR spectrum (126 MHz, $CDCl_3$) of **1d**.

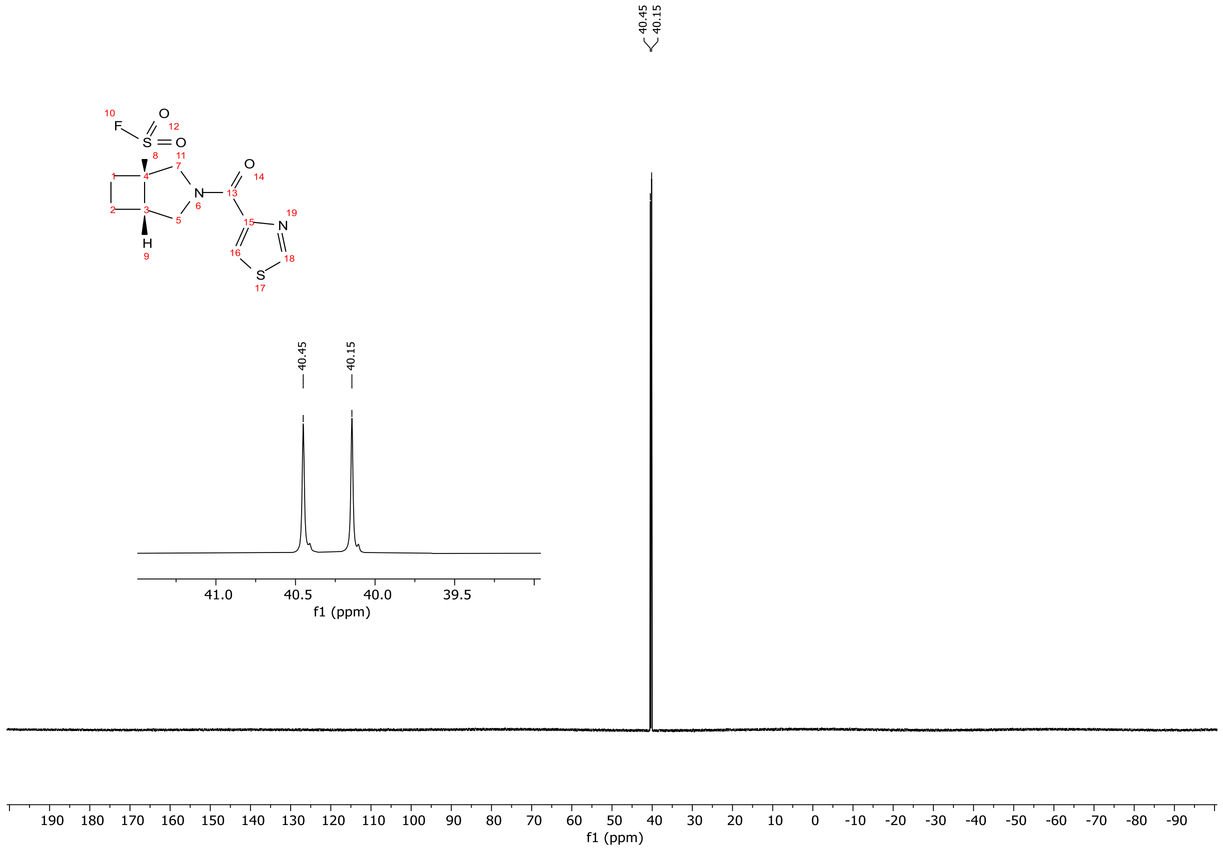

Figure S51 ^{19}F NMR spectrum (471 MHz, $CDCl_3$) of **1d**.

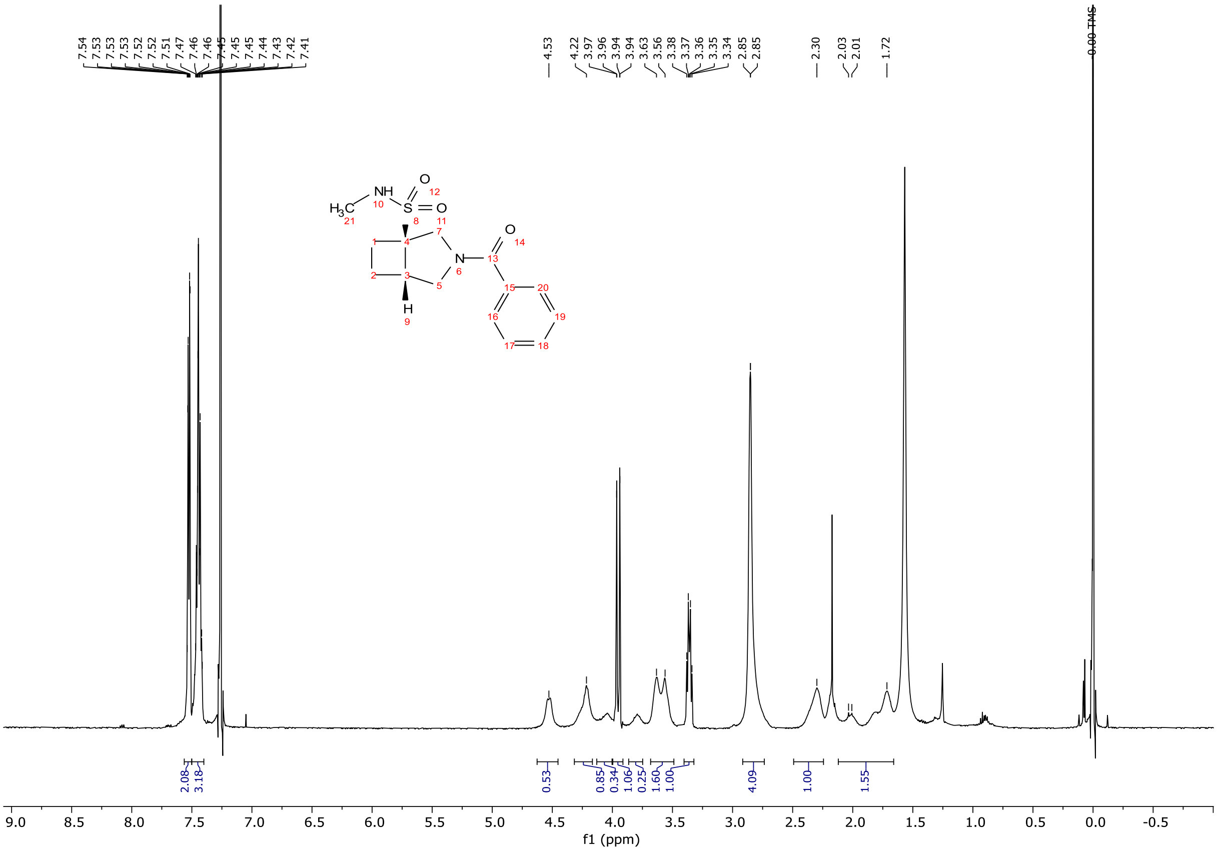

Figure S52 *^{1}H NMR spectrum (500 MHz, CDCl₃) of* ***2aa***.

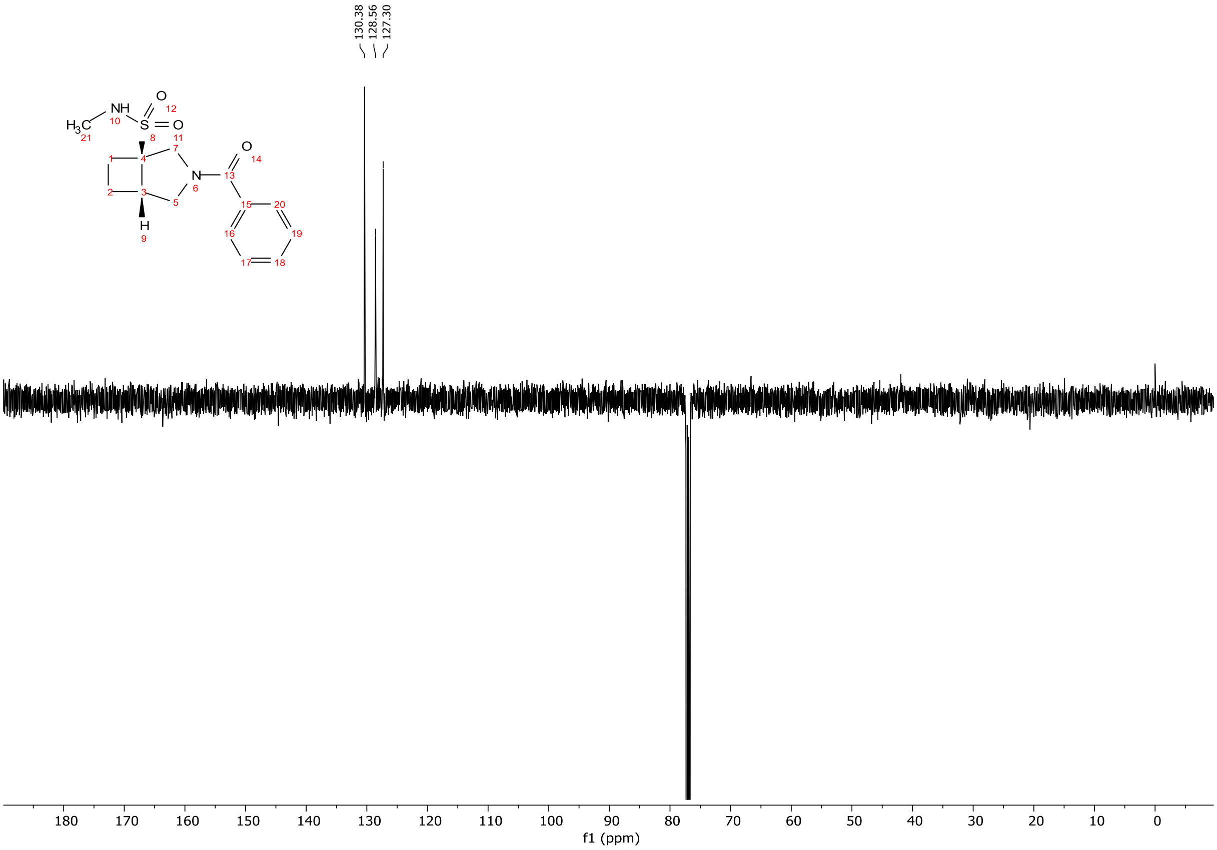

Figure S53 *^{13}C NMR spectrum (126 MHz, CDCl₃) of* ***2aa***.

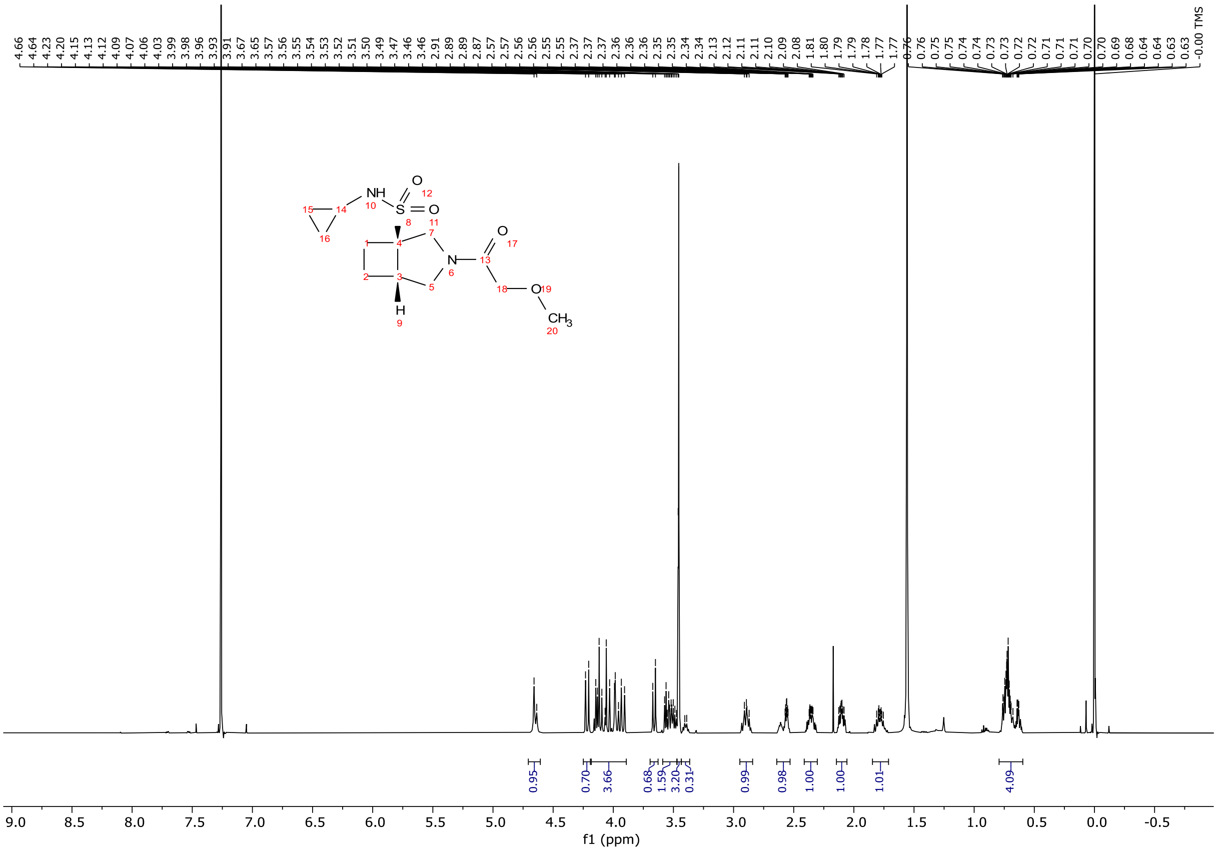

Figure S54 *^{1}H NMR spectrum (500 MHz, CDCl₃) of* ***2bb****.*

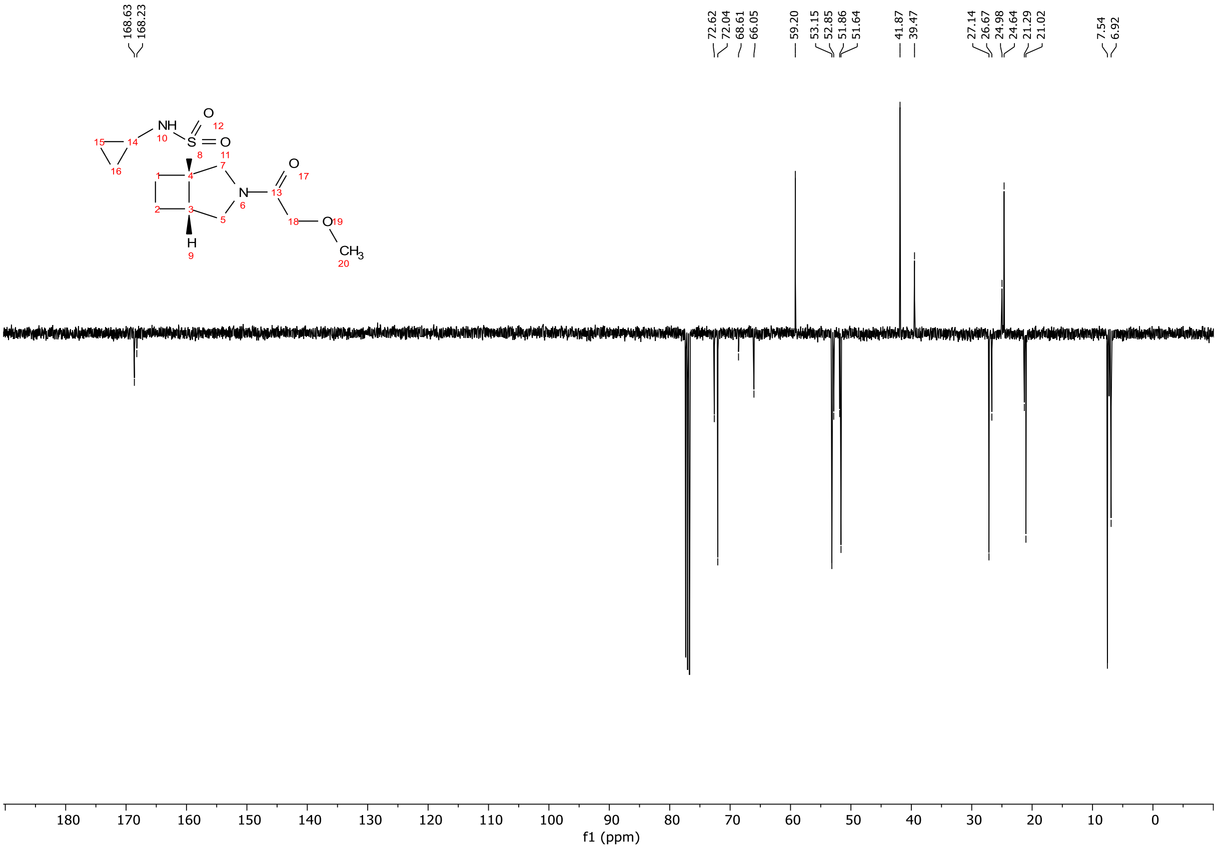

Figure S55 *^{13}C NMR spectrum (101 MHz, CDCl₃) of* ***2bb****.*

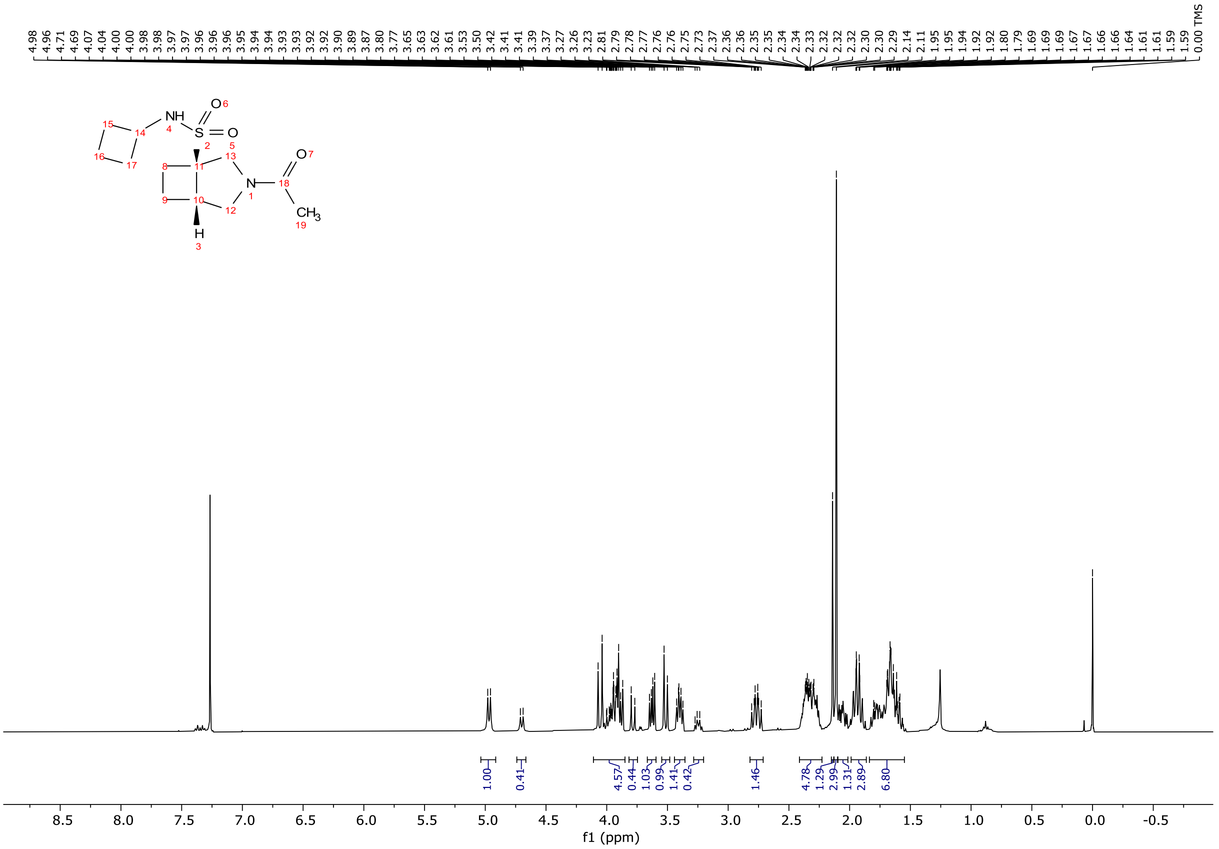

Figure S56 *^1H NMR spectrum (400 MHz, $CDCl_3$) of **2cc**.*

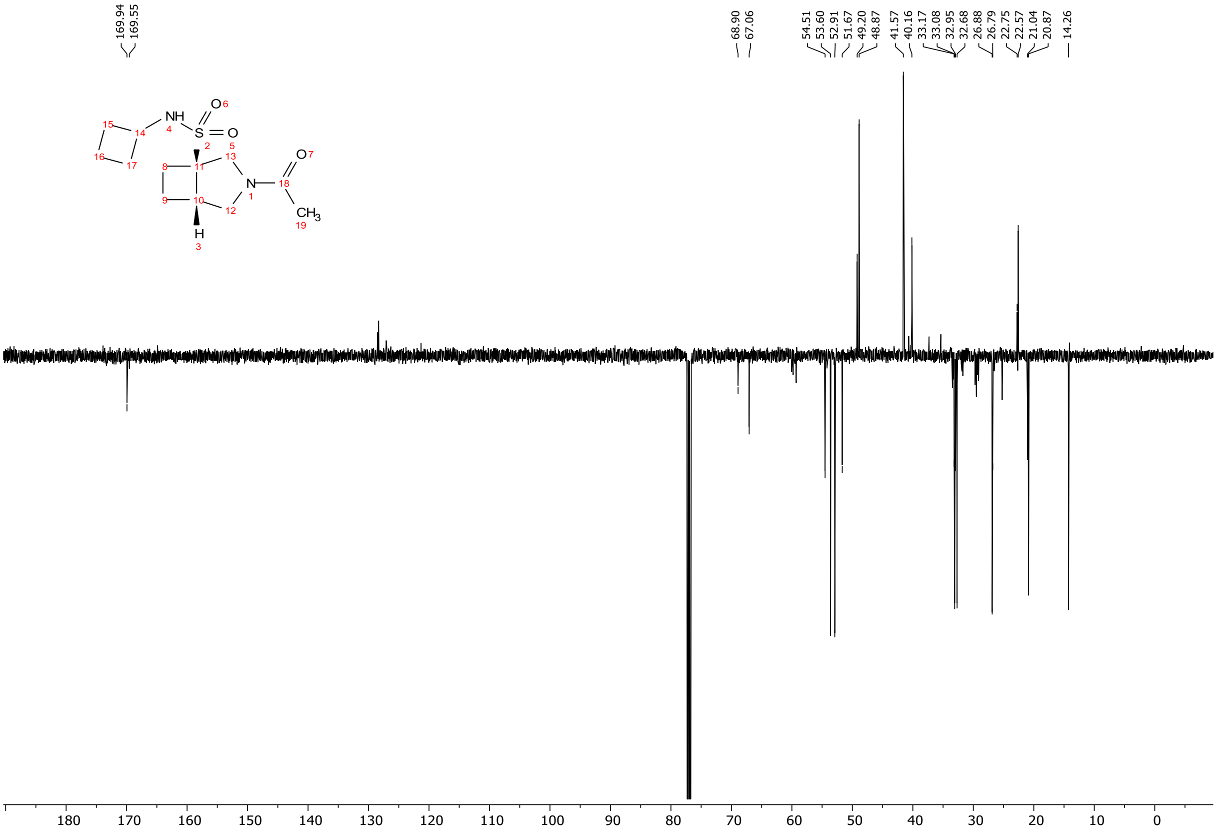

Figure S57 *^{13}C NMR spectrum (126 MHz, $CDCl_3$) of **2cc**.*

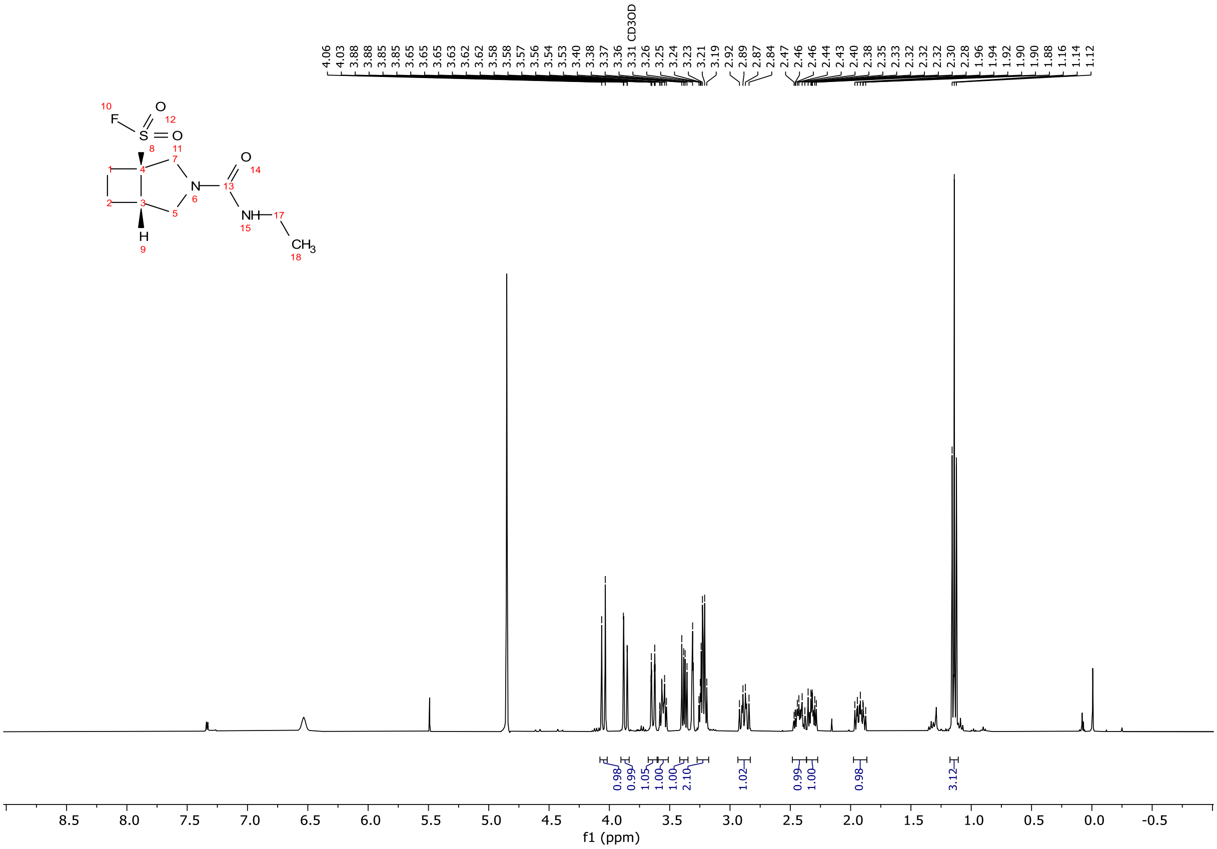

Figure S58 *^{1}H NMR spectrum (400 MHz, CD₃OD) of **1e**.*

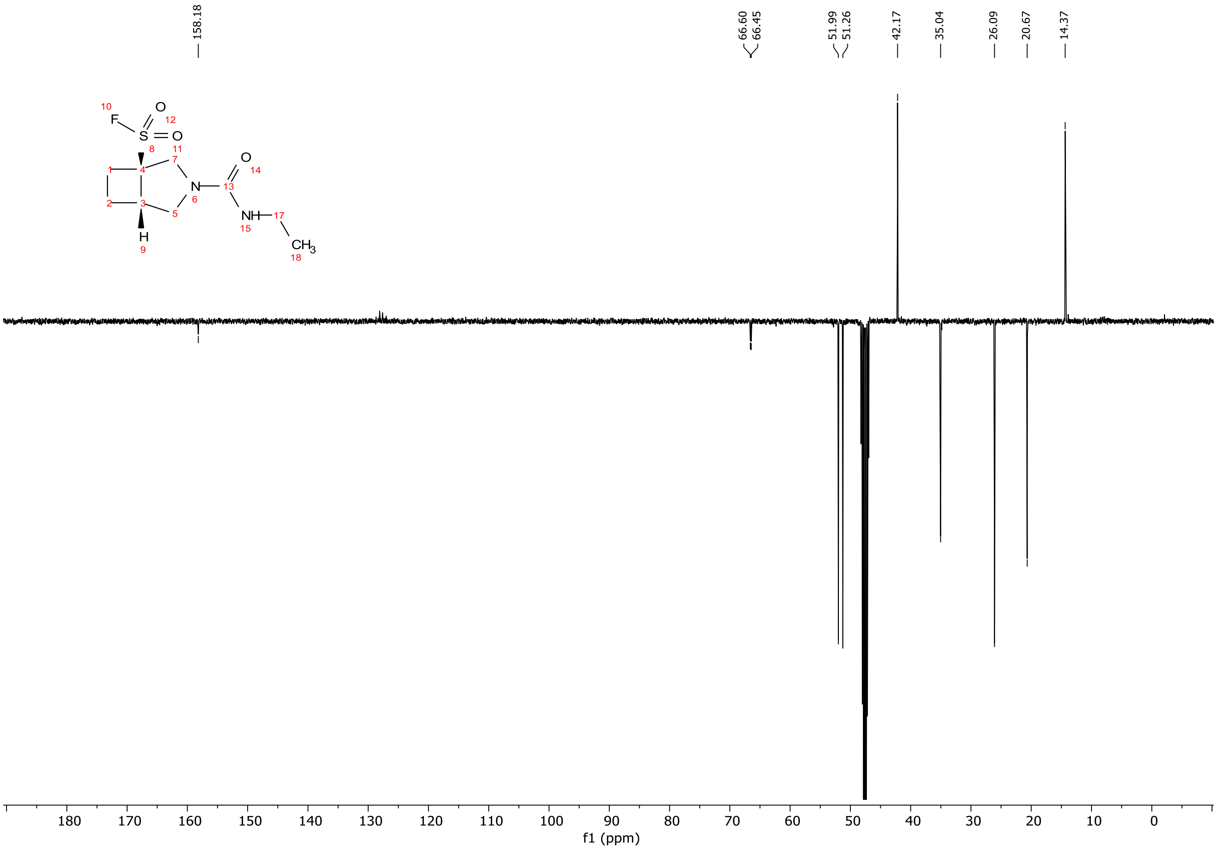

Figure S59 *^{13}C NMR spectrum (101 MHz, CD₃OD) of **1e**.*

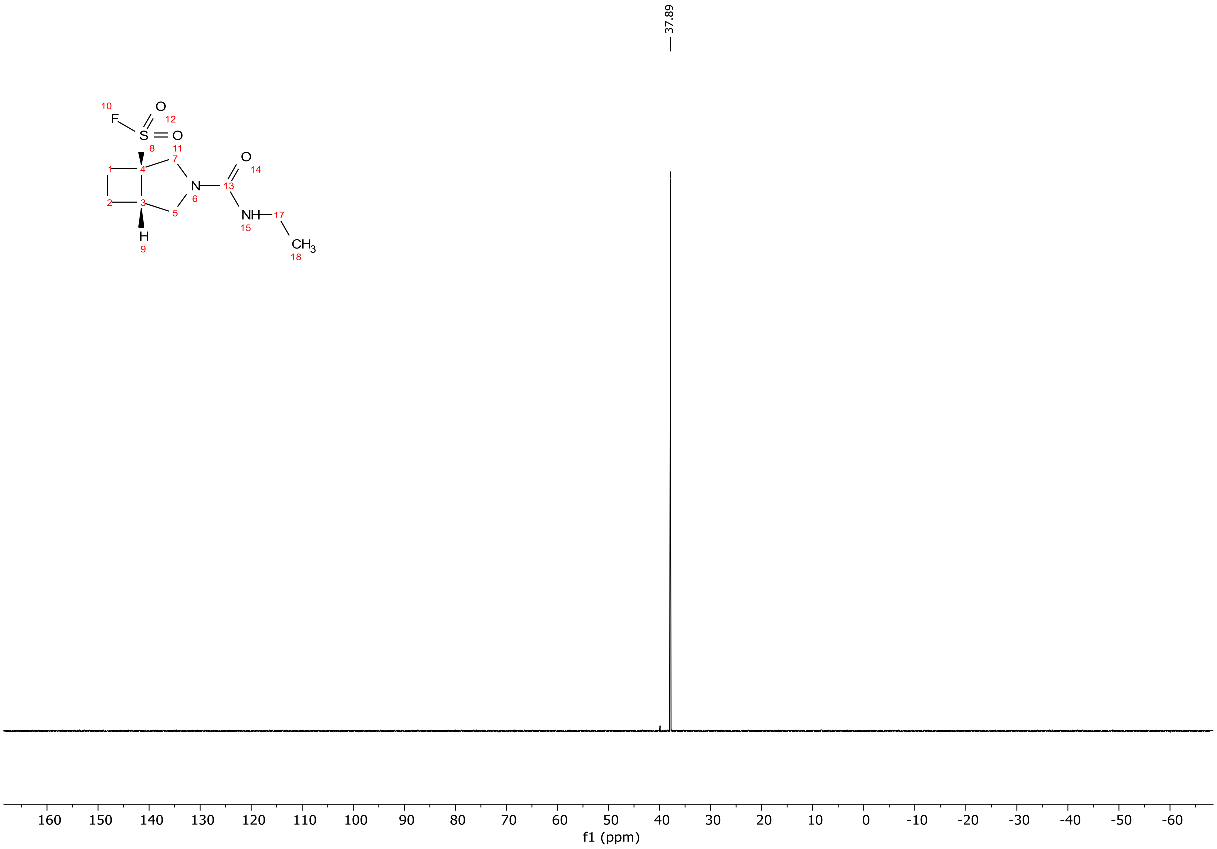

Figure S60 *^{19}F NMR spectrum (377 MHz, CD_3OD) of* ***1e***.

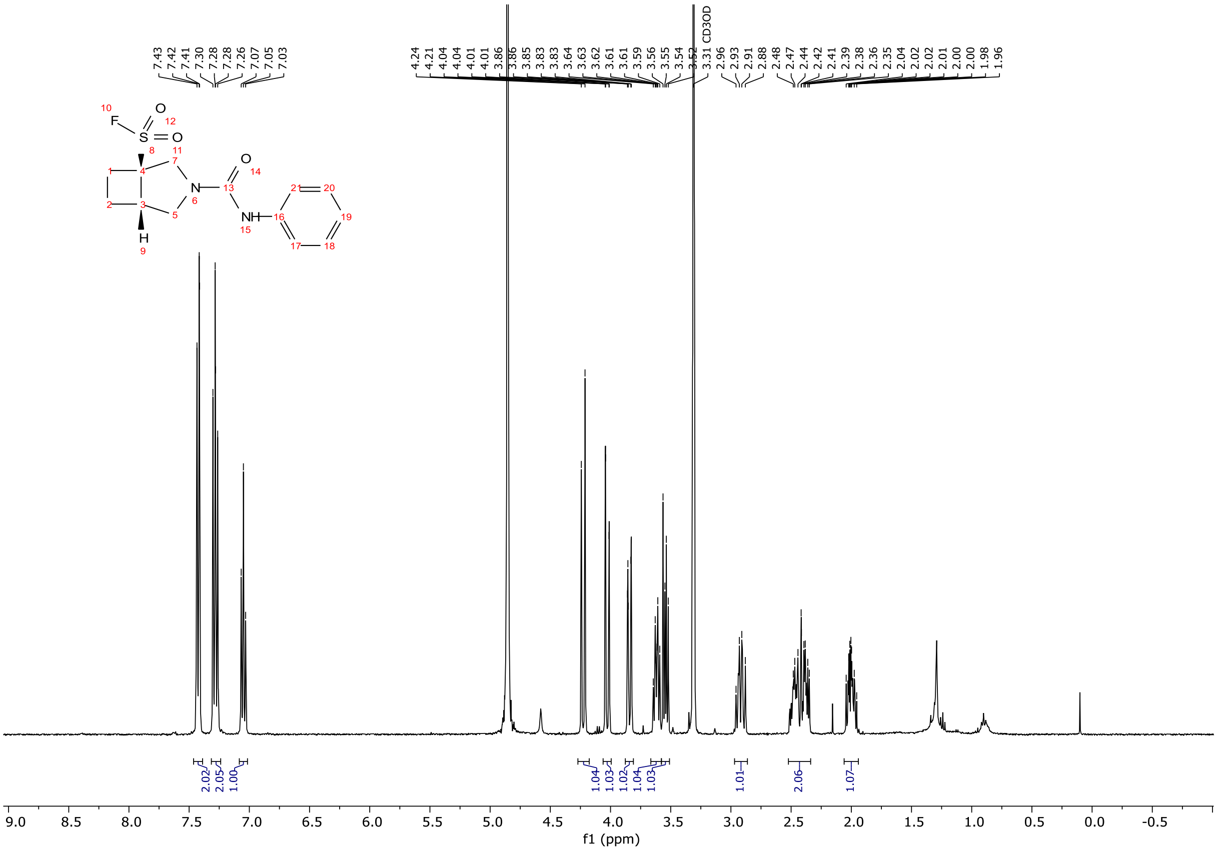

Figure S61 *^{1}H NMR spectrum (400 MHz, CD_3OD) of* ***1f***.

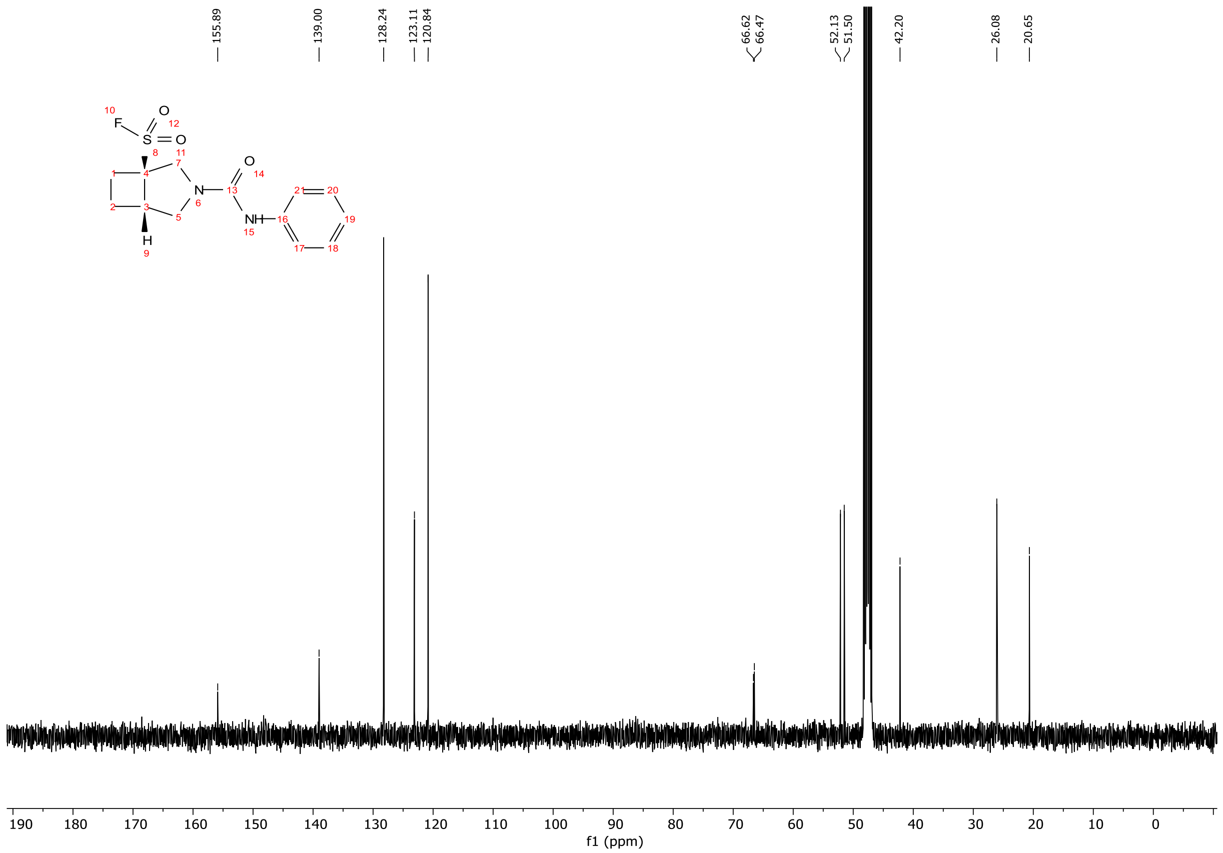

Figure S62 *^{13}C NMR spectrum (101 MHz, CD_3OD) of **1f**.*

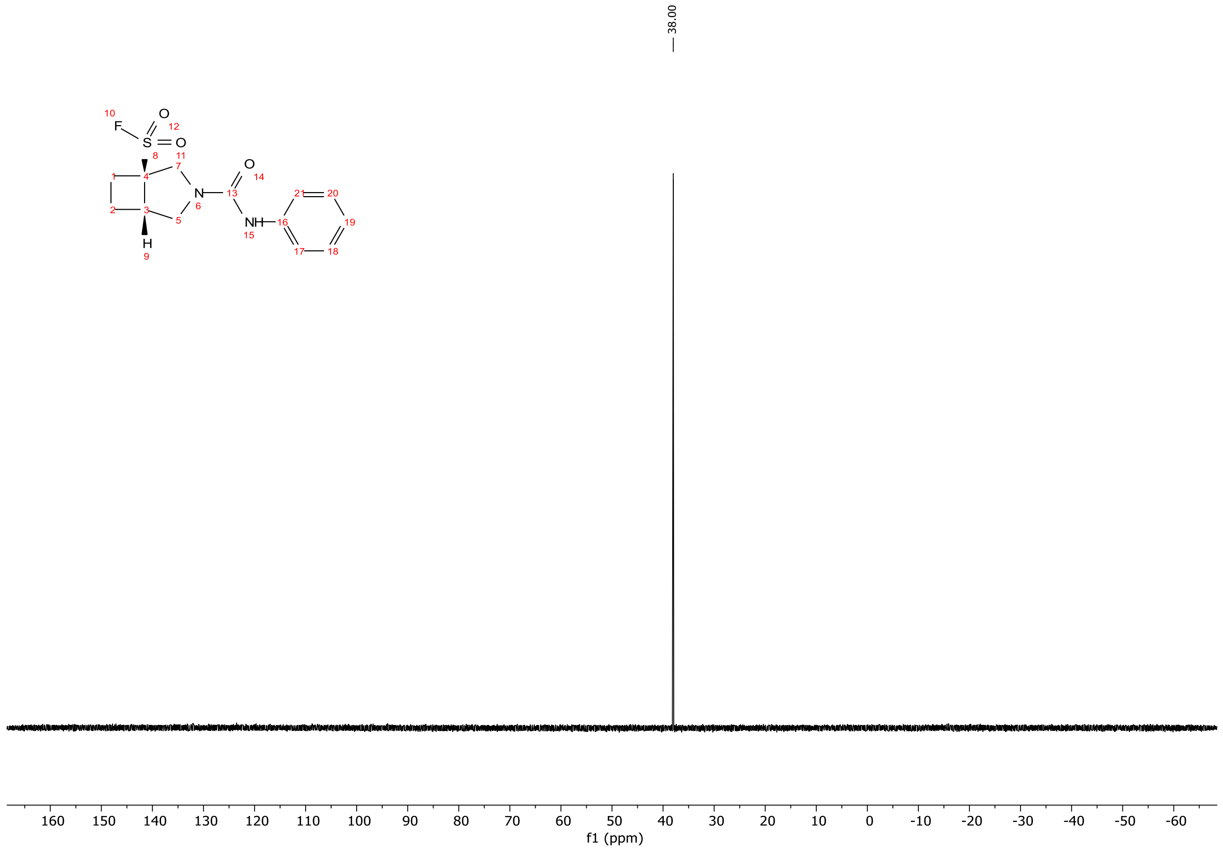

Figure S63 *^{19}F NMR spectrum (377 MHz, CD_3OD) of **1f**.*

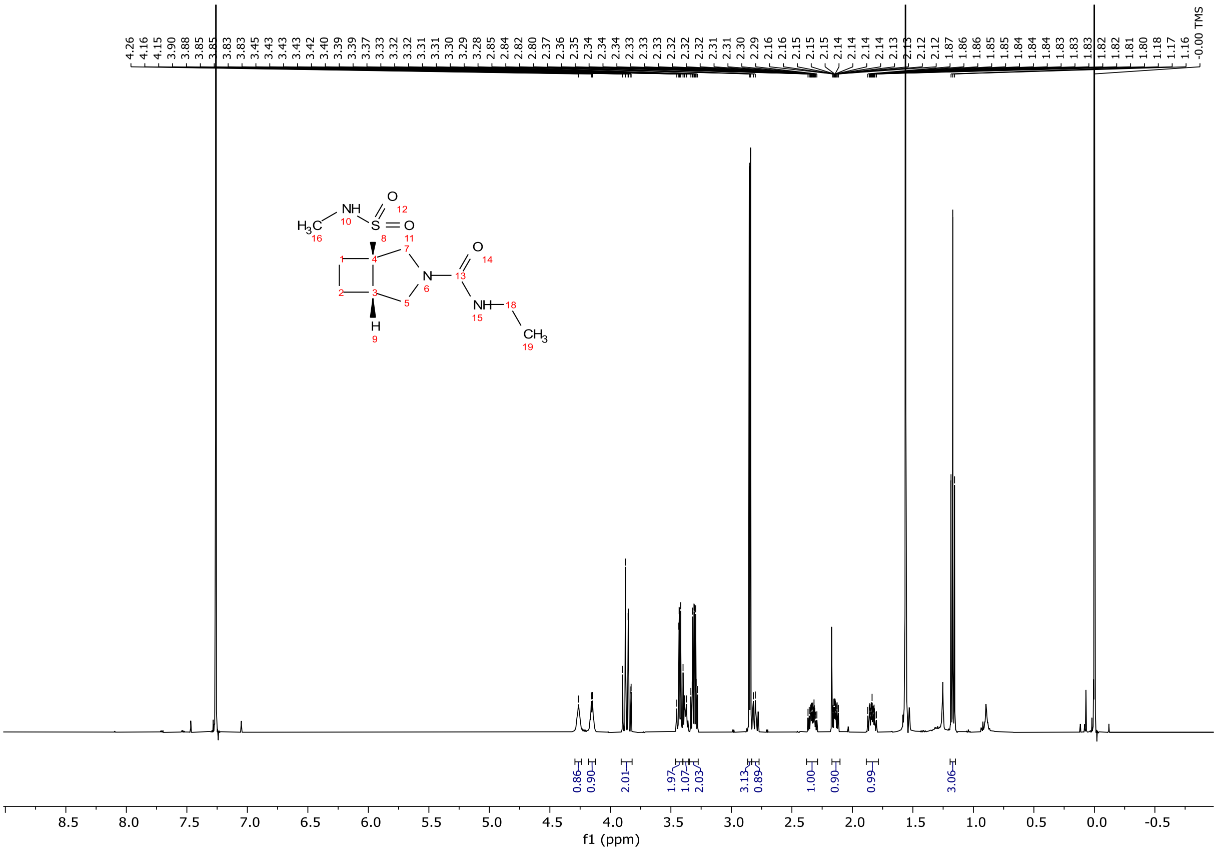

Figure S64 ^1H *NMR spectrum (500 MHz, $CDCl_3$) of* ***2ad***.

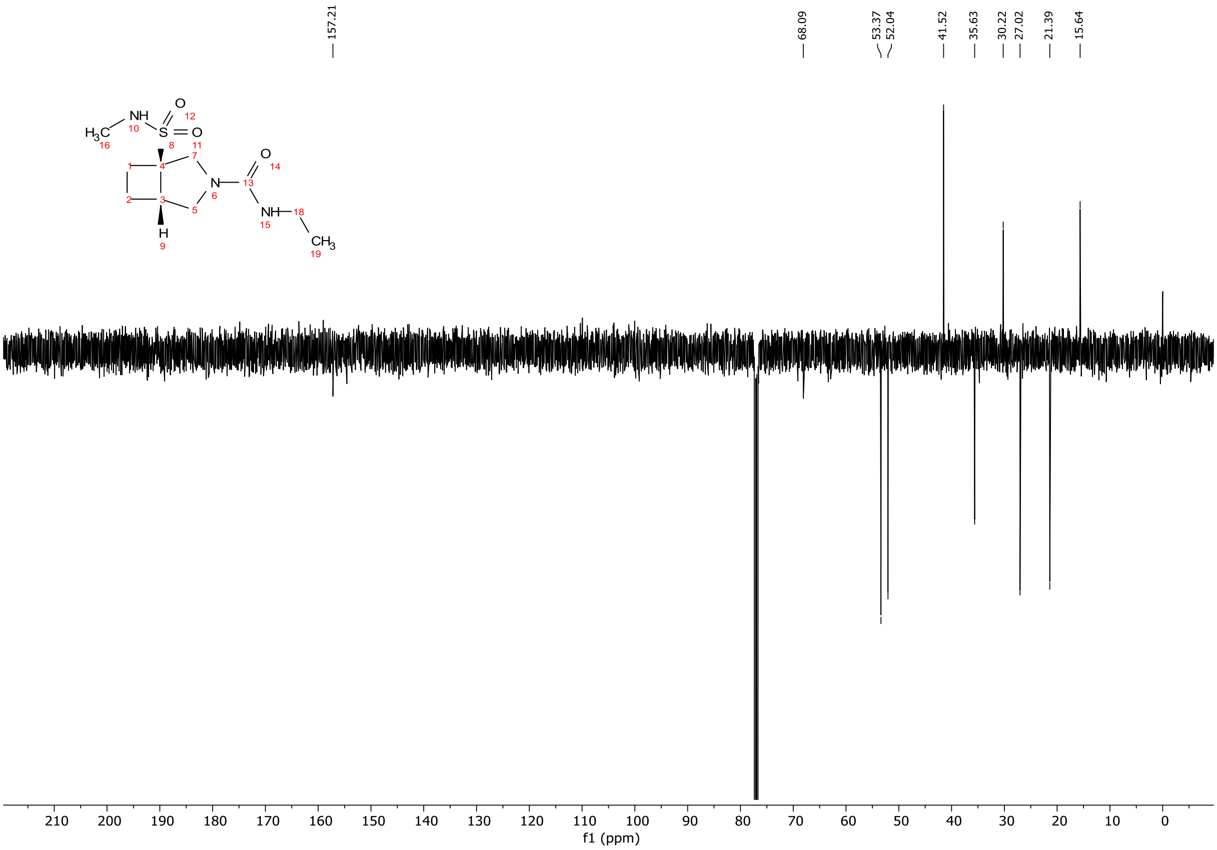

Figure S65 ^{13}C *NMR spectrum (126 MHz, $CDCl_3$) of* ***2ad***.

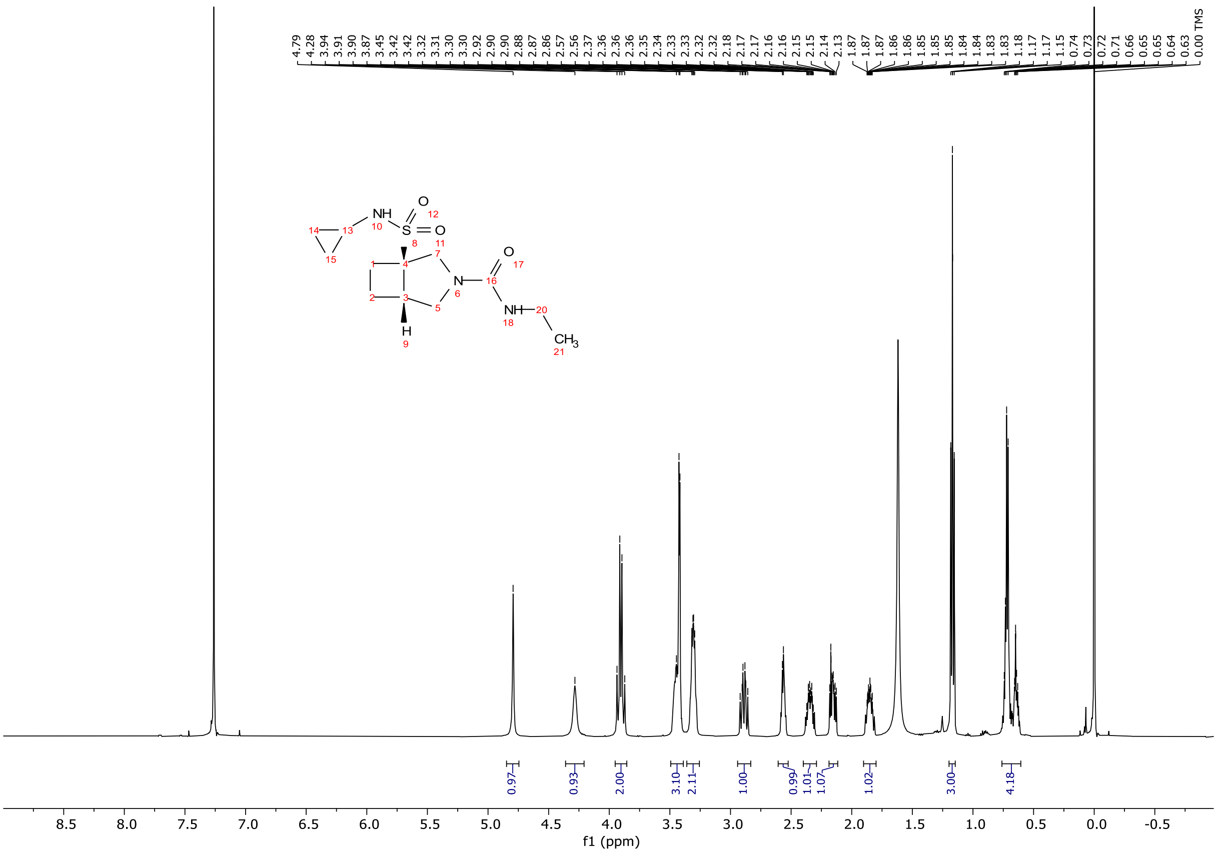

Figure S66 ^{1}H NMR spectrum (500 MHz, $CDCl_3$) of **2be**.

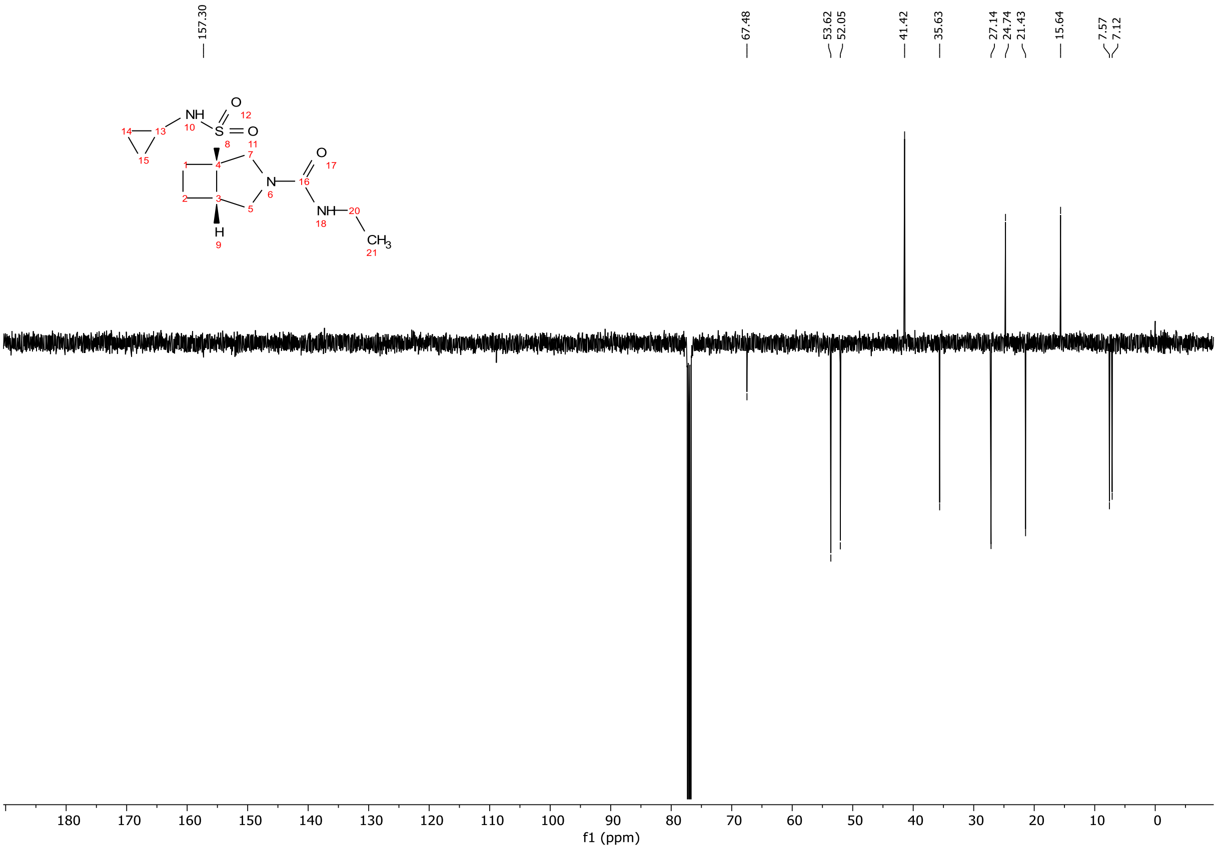

Figure S67 ^{13}C NMR spectrum (126 MHz, $CDCl_3$) of **2be**.

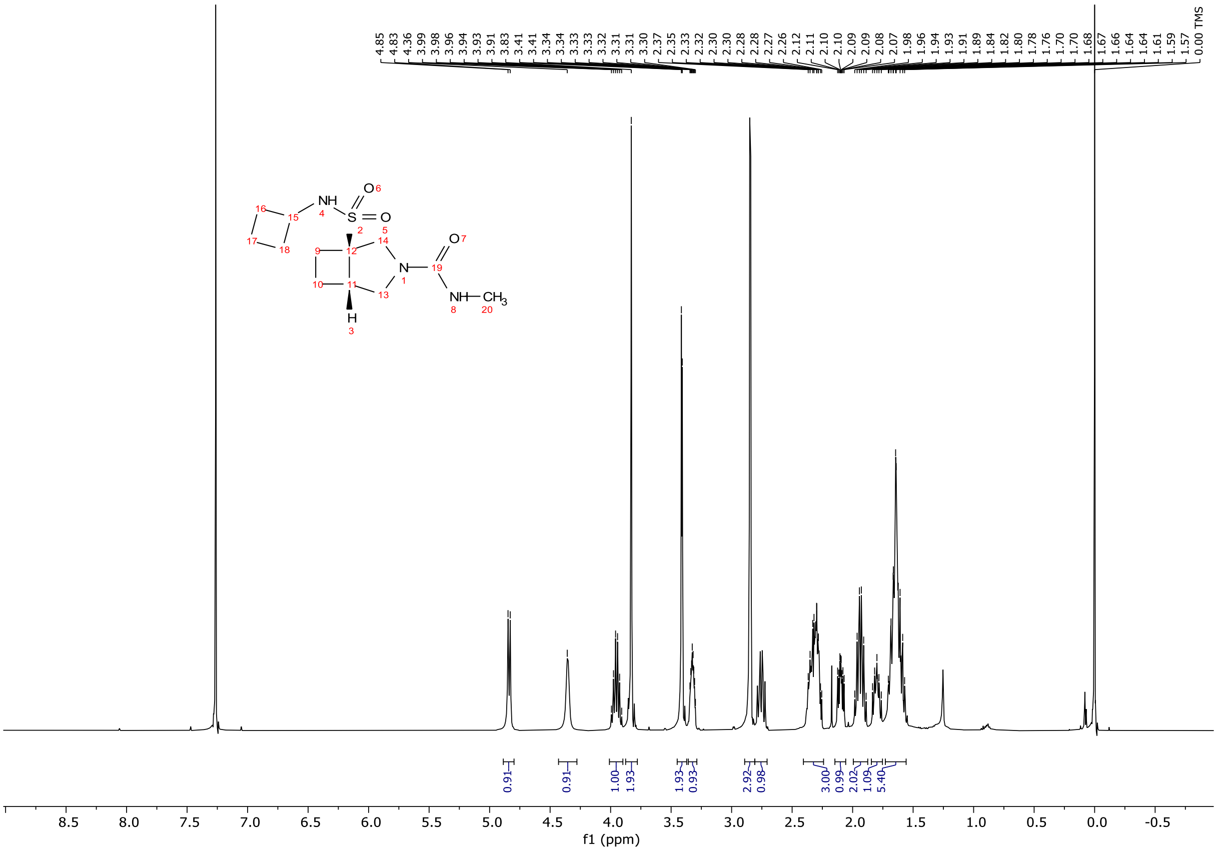

Figure S68 *^{1}H NMR spectrum (500 MHz, CDCl₃) of **2cf**.*

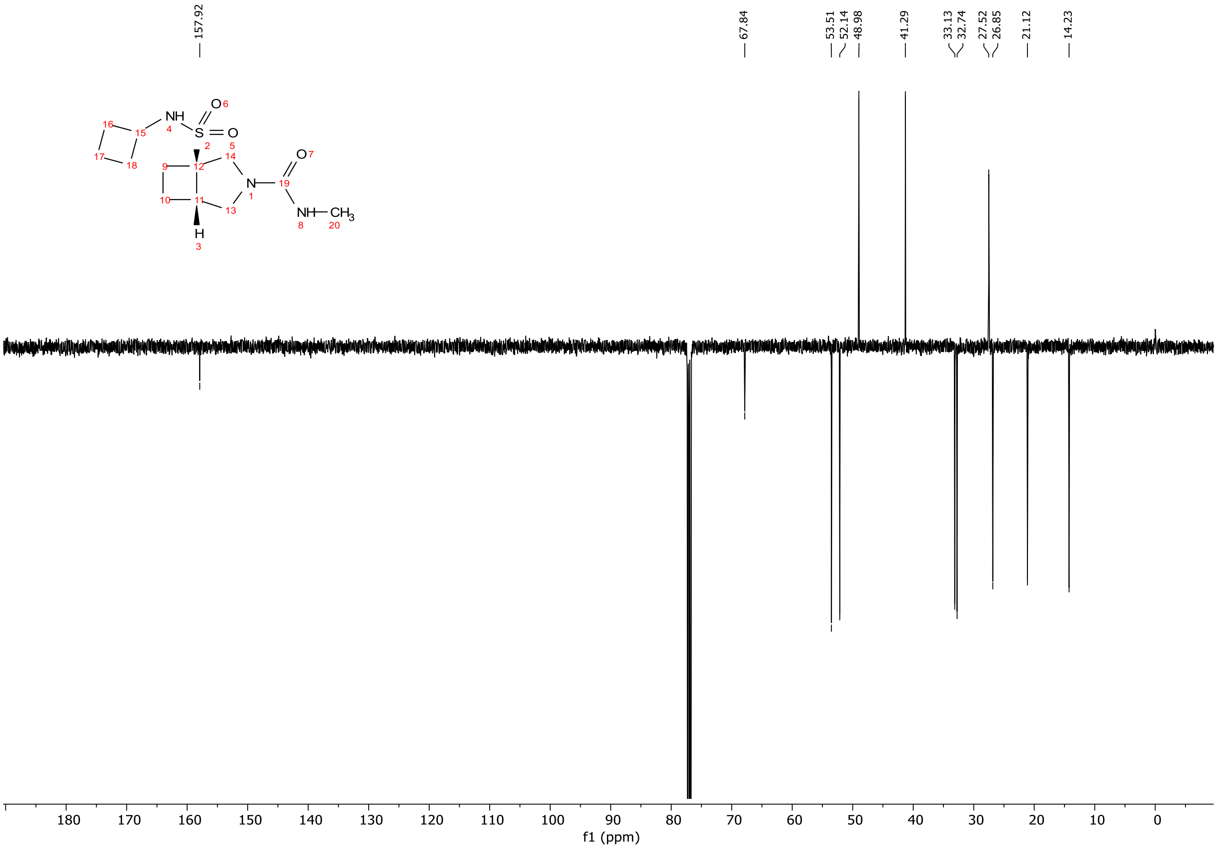

Figure S69 *^{13}C NMR spectrum (126 MHz, CDCl₃) of **2cf**.*

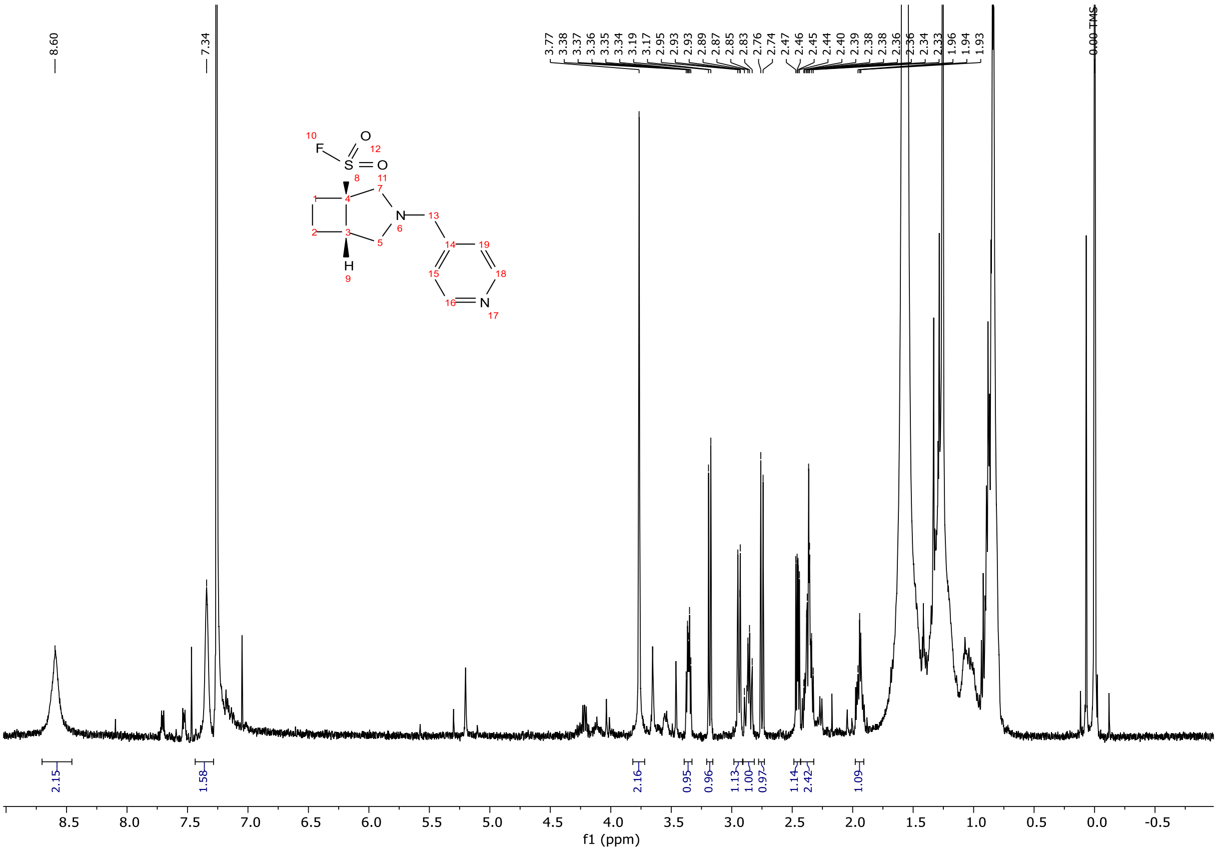

Figure S70 *^{1}H NMR spectrum (500 MHz, $CDCl_3$) of* ***1g***.

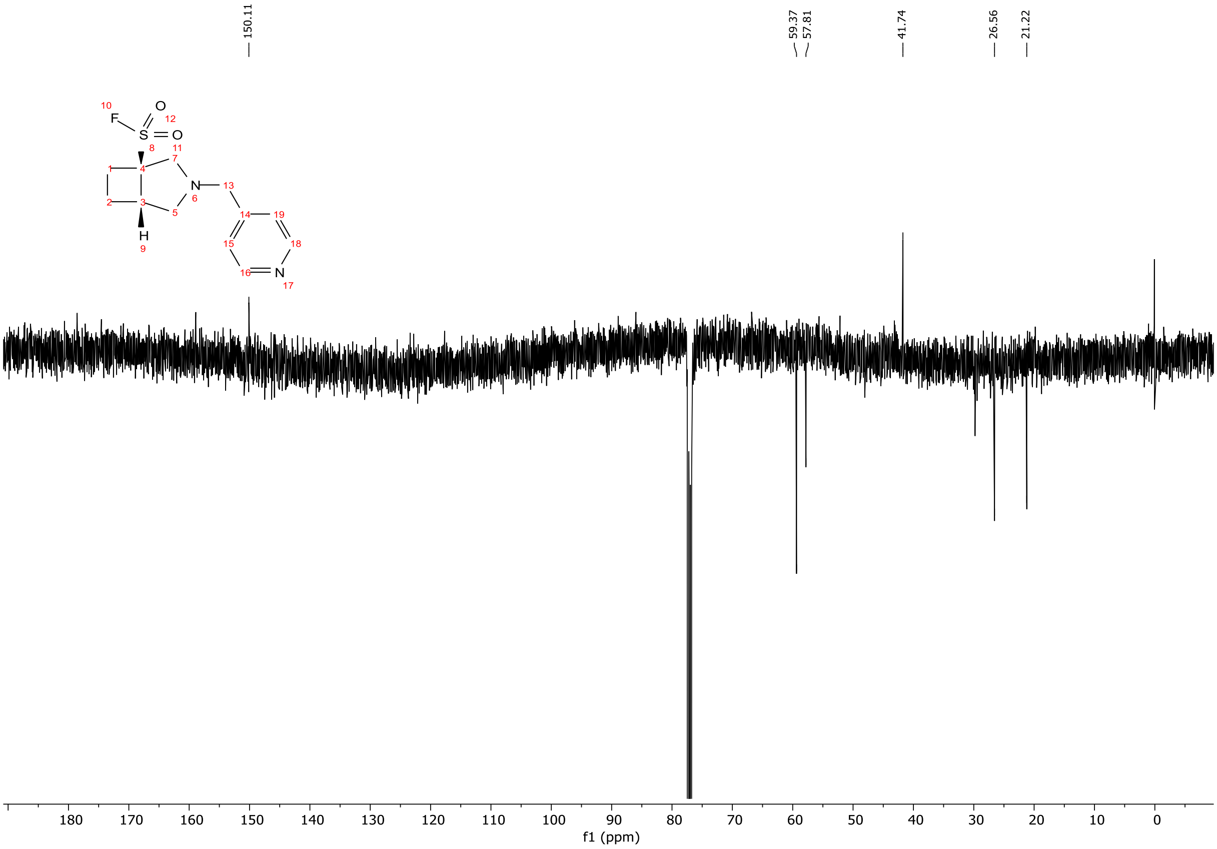

Figure S71 *^{13}C NMR spectrum (126 MHz, $CDCl_3$) of* ***1g***.

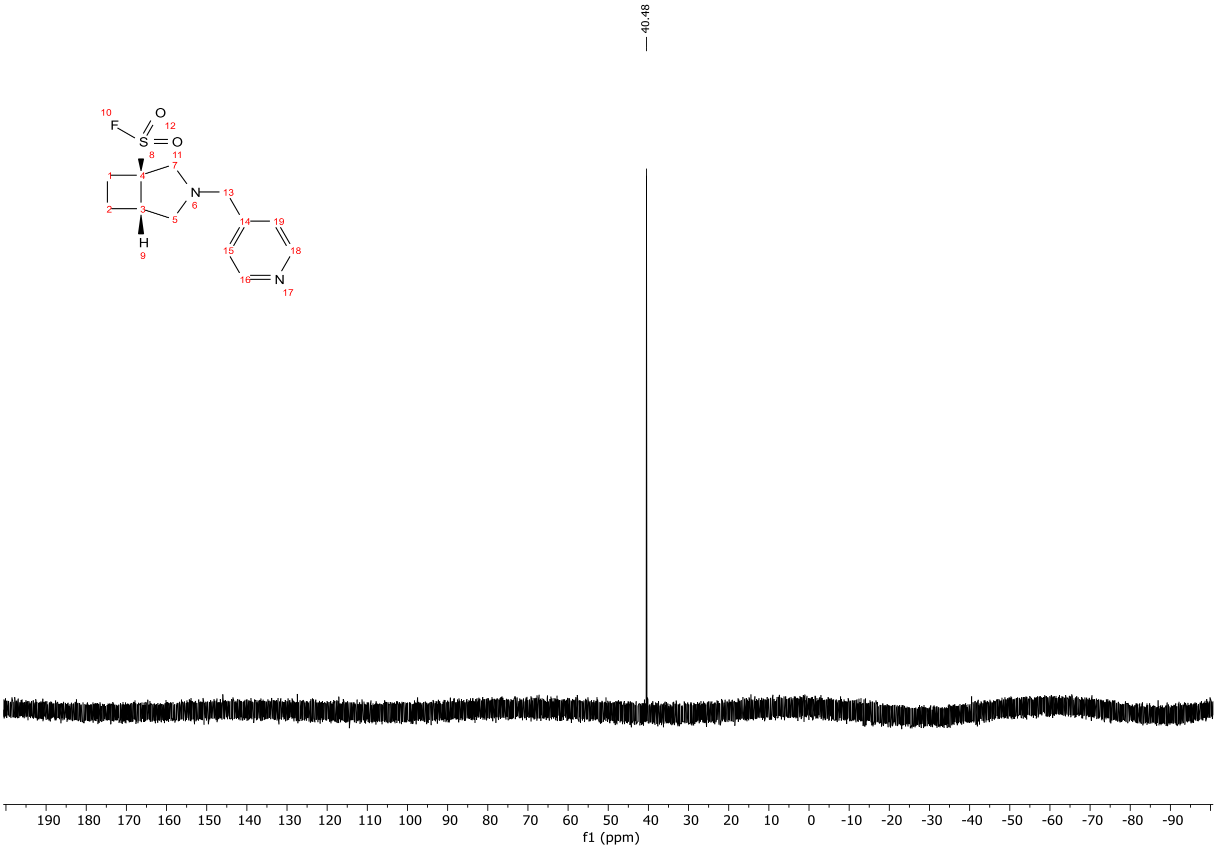

Figure S72 *^{19}F NMR spectrum (471 MHz, $CDCl_3$) of* ***1g***.

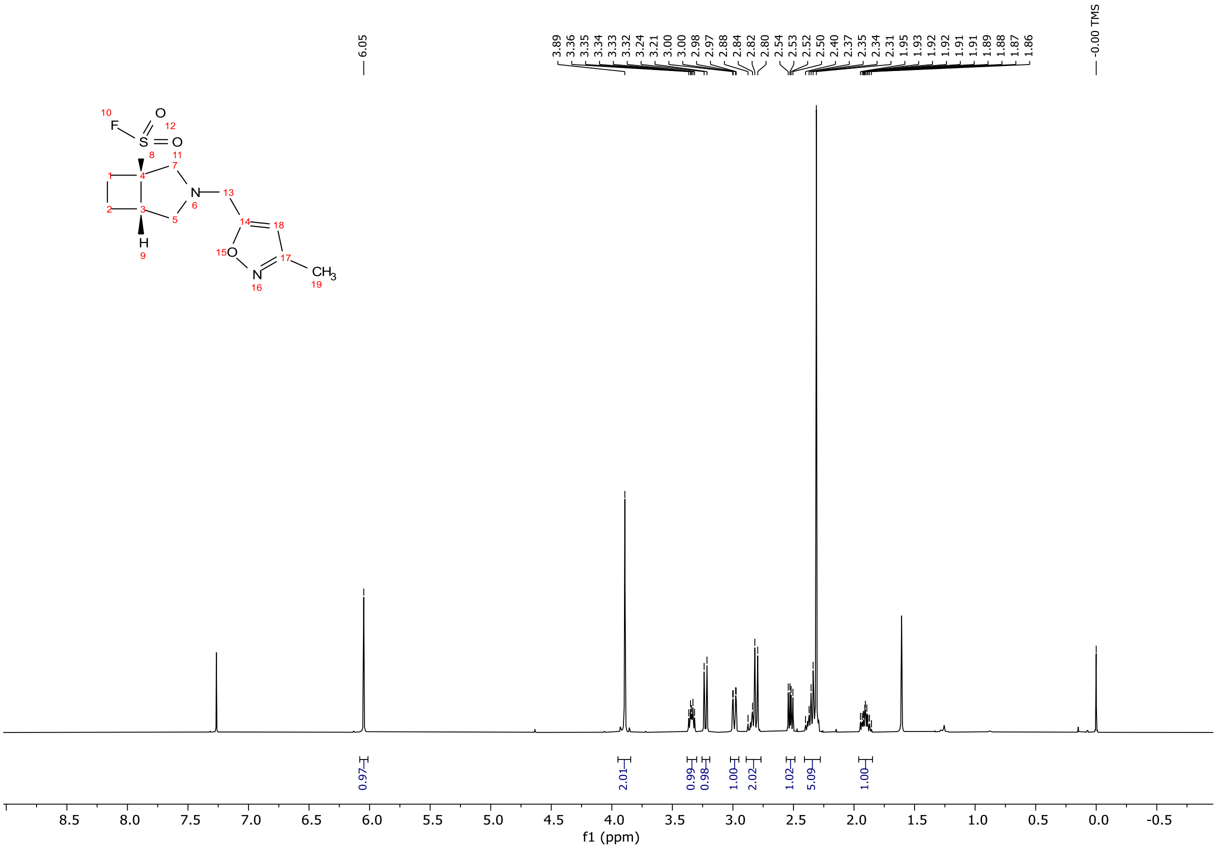

Figure S73 *^{1}H NMR spectrum (400 MHz, $CDCl_3$) of* ***1h***.

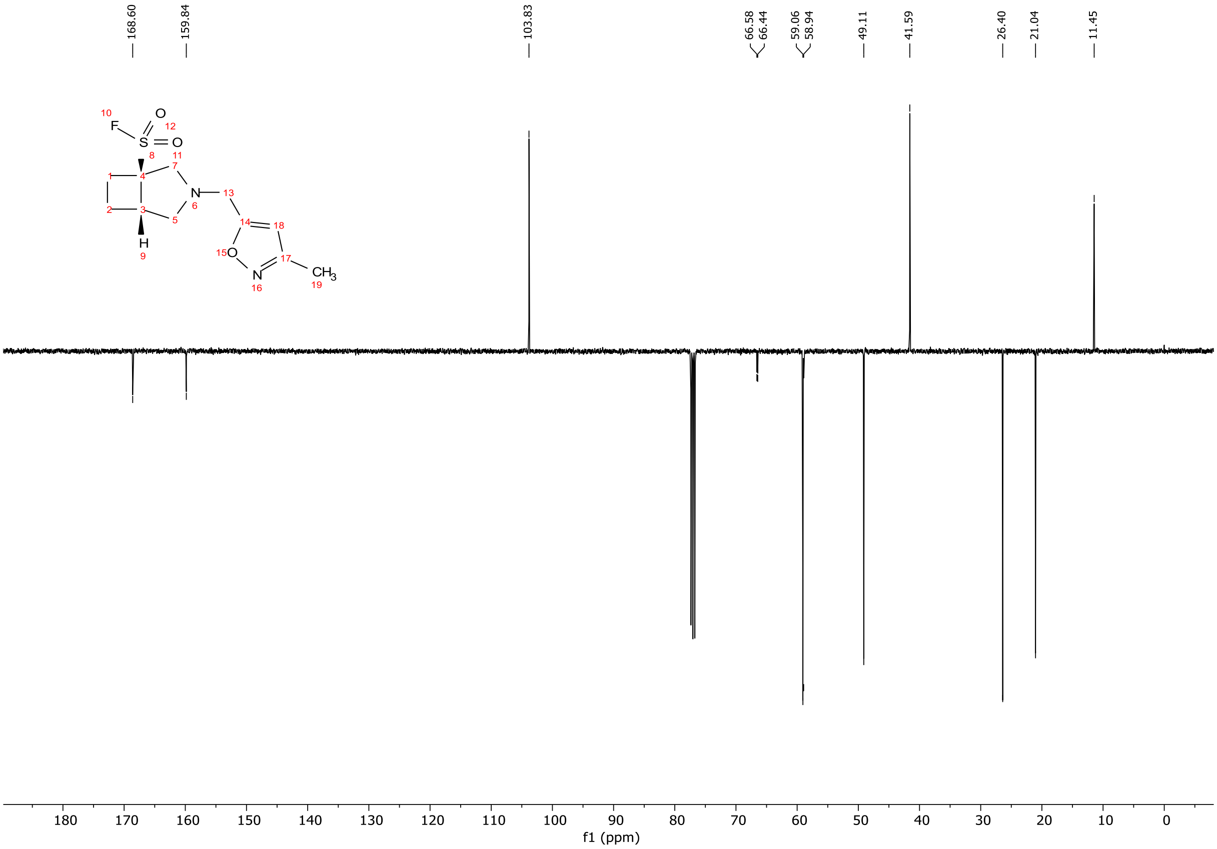

Figure S74 *^{13}C NMR spectrum (101 MHz, CDCl₃) of **1h**.*

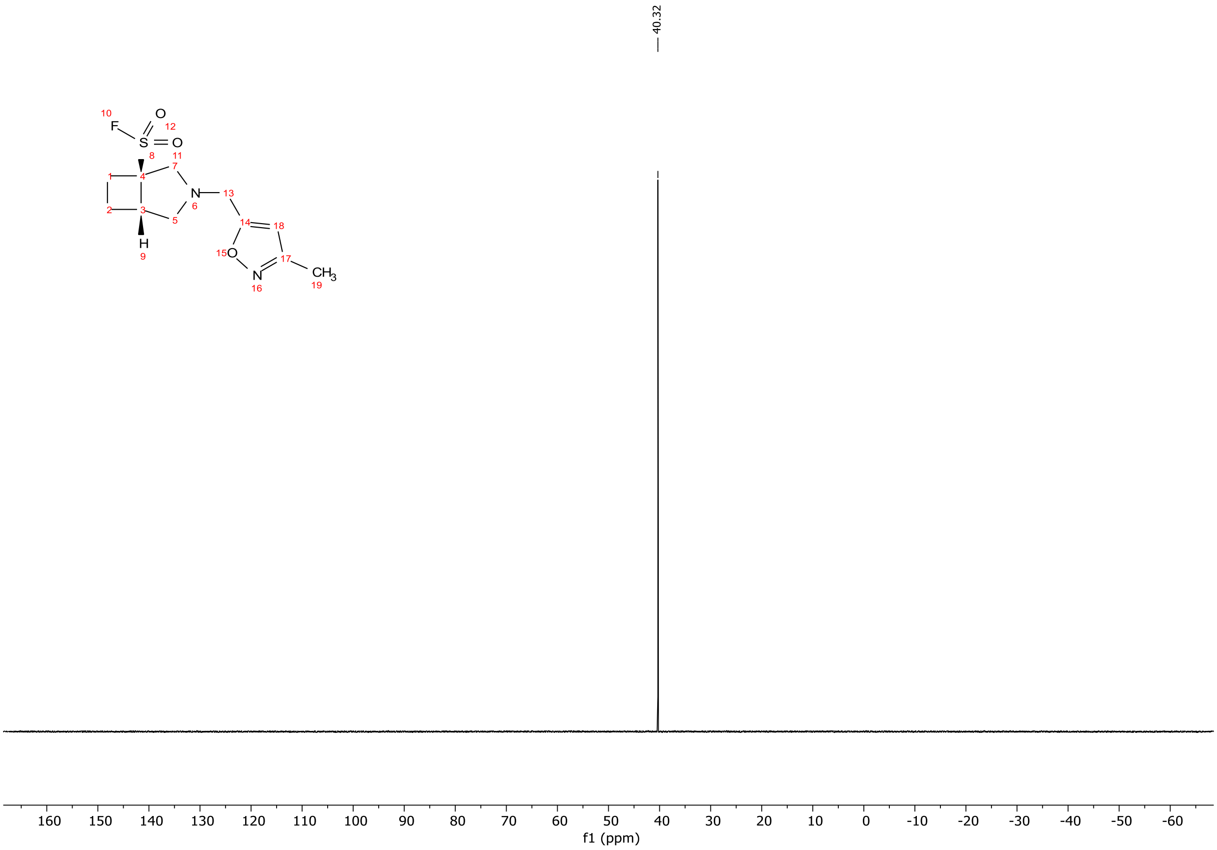

Figure S75 *^{19}F NMR spectrum (377 MHz, CDCl₃) of **1h**.*

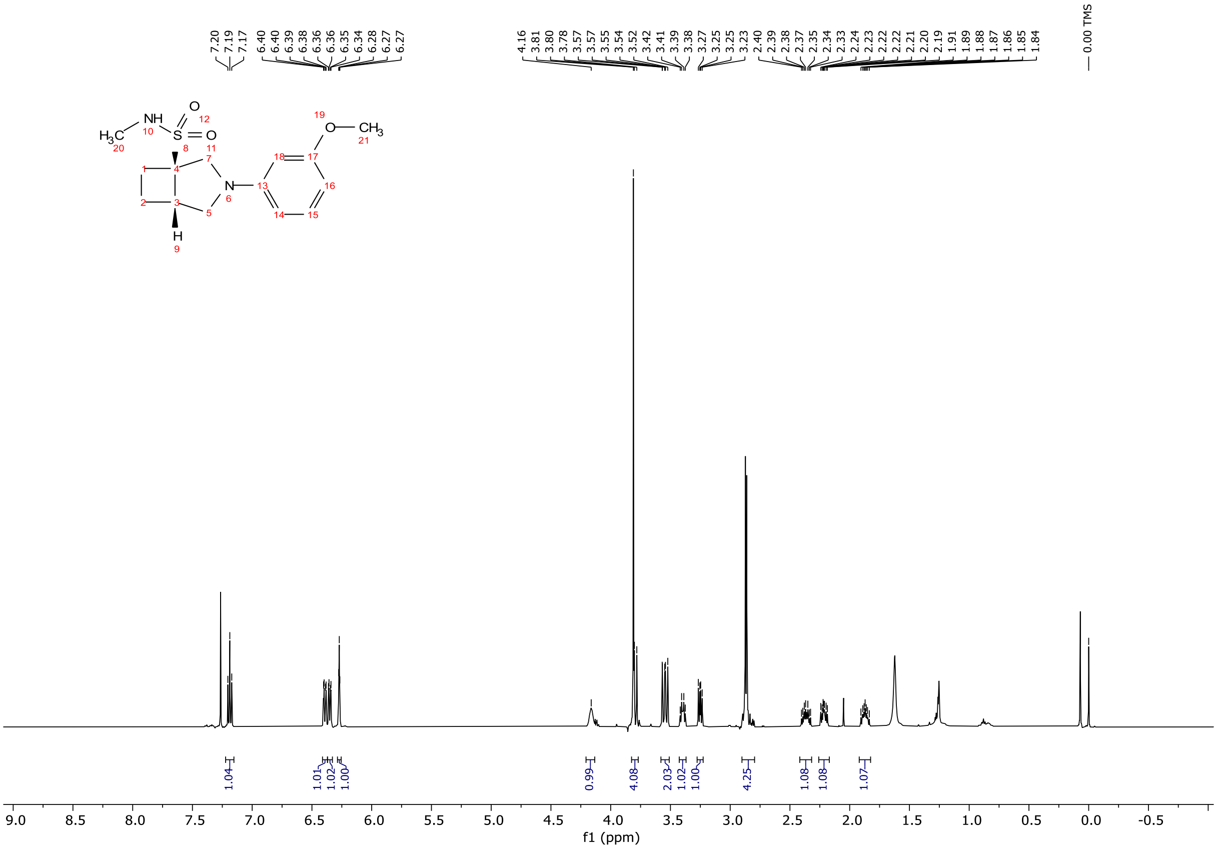

Figure S76 *^{1}H NMR spectrum (500 MHz, CDCl₃) of* ***2ag****.*

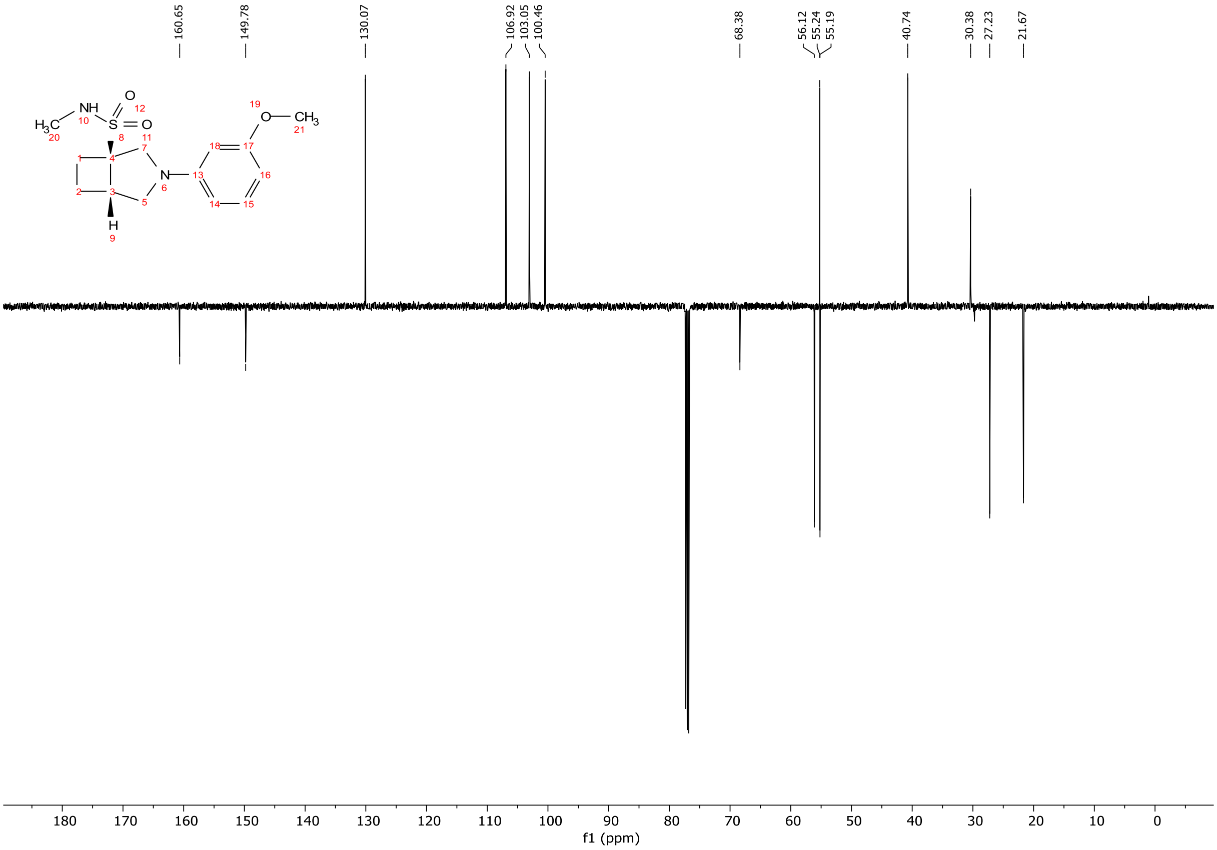

Figure S77 *^{13}C NMR spectrum (126 MHz, CDCl₃) of* ***2ag****.*

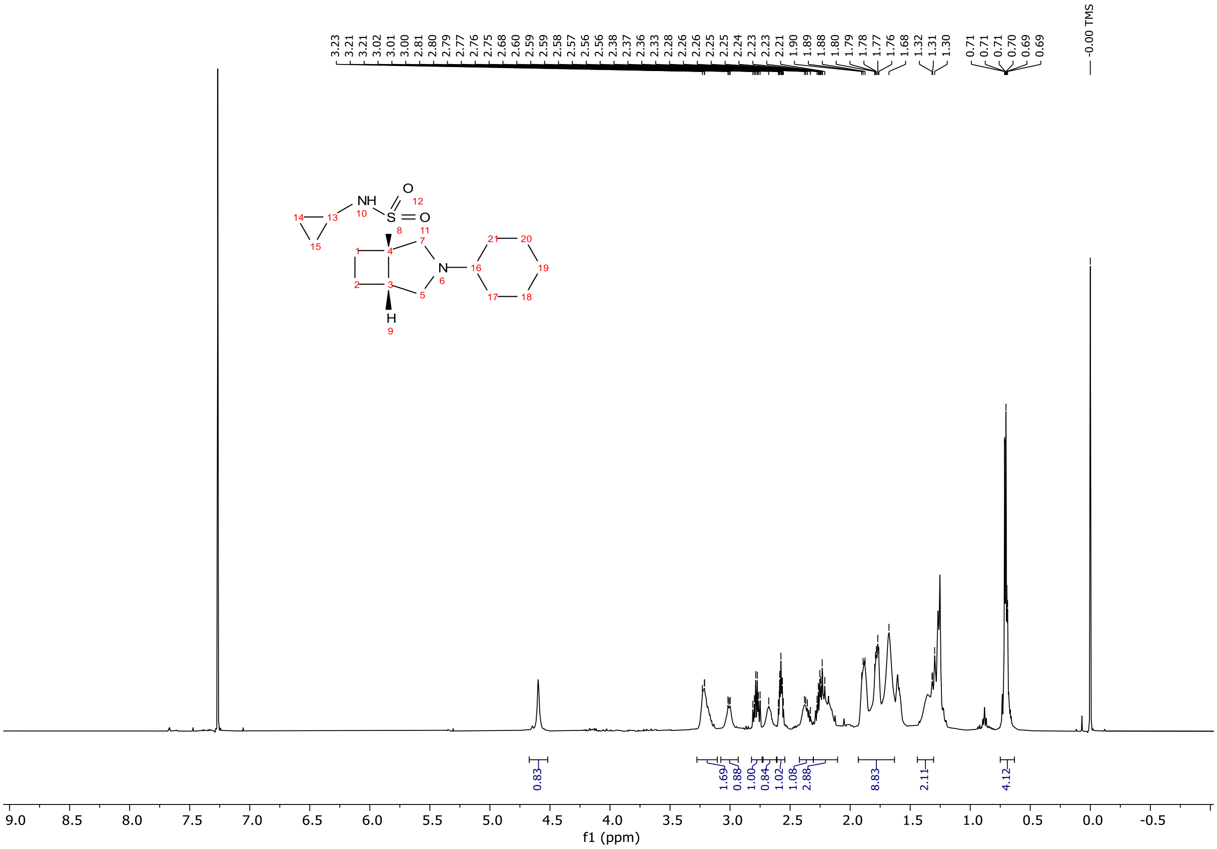

Figure S78 *^{1}H NMR spectrum (500 MHz, $CDCl_3$) of* ***2bh****.*

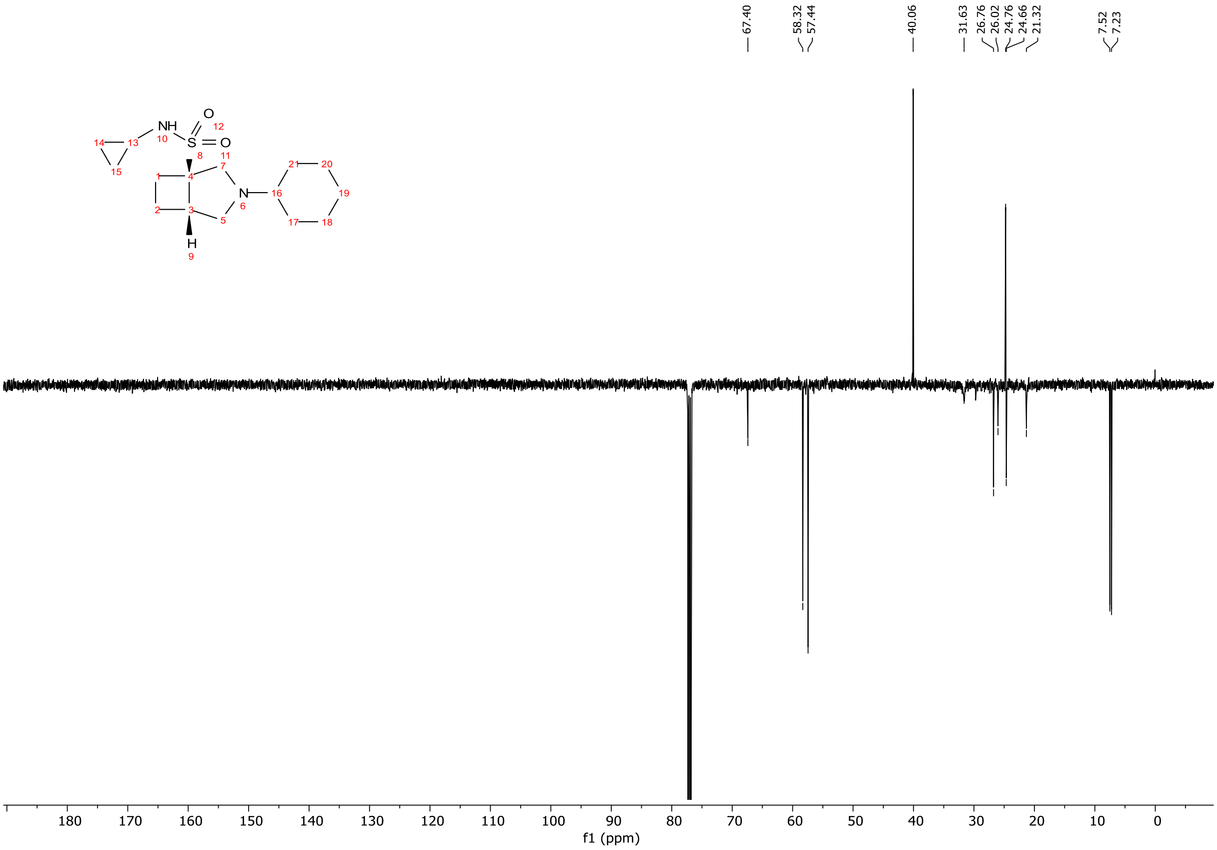

Figure S79 *^{13}C NMR spectrum (126 MHz, $CDCl_3$) of* ***2bh****.*

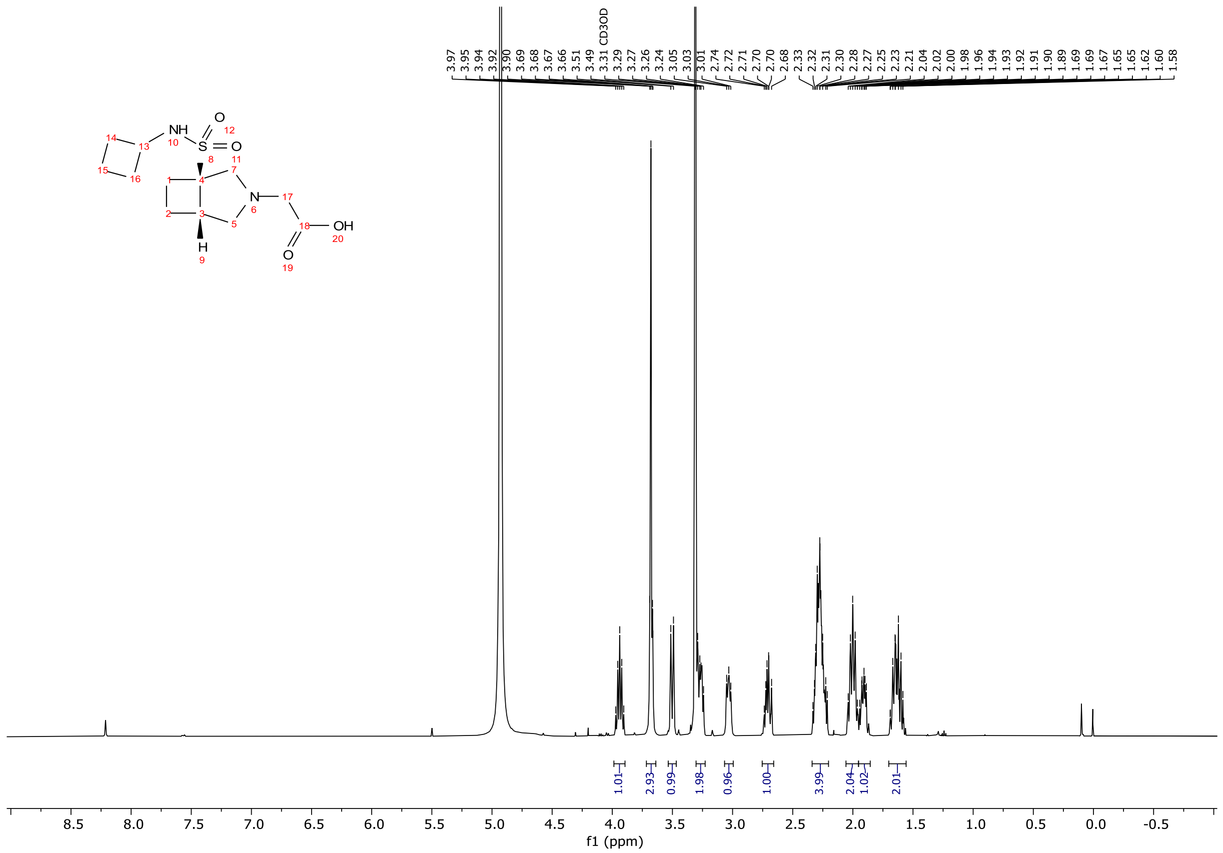

Figure S80 *^{1}H NMR spectrum (500 MHz, CD_3OD) of* ***2ci***.

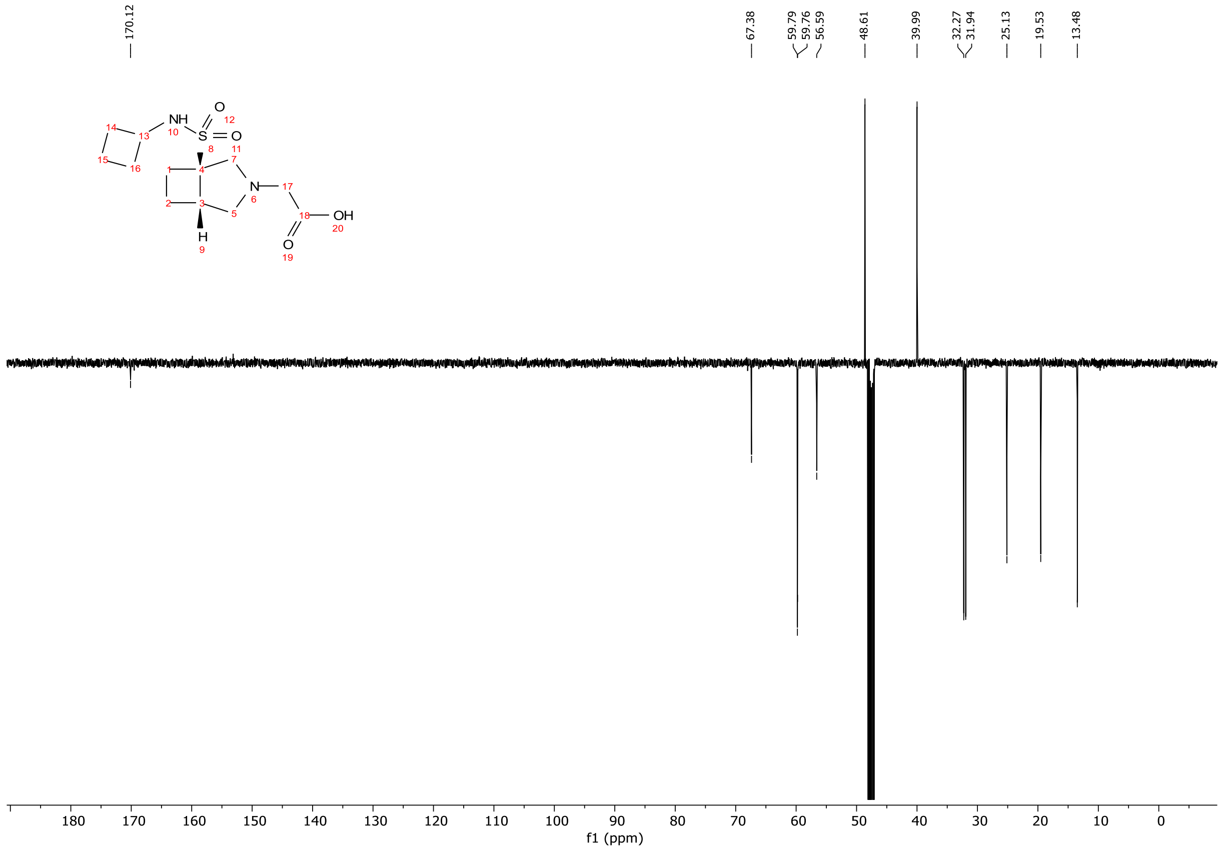

Figure S81 *^{13}C NMR spectrum (126 MHz, CD_3OD) of* ***2ci***.

4) X-Ray Crystal Structure Data

Crystals were obtained by slow evaporation of a solution of compound **3** in a heptane:EtOAc 4:1 mixture at room temperature.

Deposition Number 2380113 (for **3**) contains the supplementary crystallographic data for this paper. These data are provided free of charge by the joint Cambridge Crystallographic Data Centre (CCDC) and Fachinformationszentrum Karlsruhe Access Structures service.

```
Bond precision:    C-C = 0.0019 A          Wavelength=0.71073

Cell:               a=11.9373(3)     b=12.2650(3)      c=17.7321(5)
                    alpha=90         beta=90           gamma=90
Temperature:        150 K

                  Calculated                Reported
Volume            2596.17(12)               2596.17(12)
Space group       P b c a                   P b c a
Hall group        -P 2ac 2ab                -P 2ac 2ab
Moiety formula    C13 H16 F N O2 S          C13 H16 F N O2 S
Sum formula       C13 H16 F N O2 S          C13 H16 F N O2 S
Mr                269.33                    269.33
Dx,g cm-3         1.378                     1.378
Z                 8                         8
Mu (mm-1)         0.255                     0.255
F000              1136.0                    1136.0
F000'             1137.55
h,k,lmax          15,16,23                  15,16,23
Nref              3233                      3225
Tmin,Tmax         0.925,0.970               0.523,0.746
Tmin'             0.925

Correction method= # Reported T Limits: Tmin=0.523 Tmax=0.746
AbsCorr = MULTI-SCAN

Data completeness= 0.998            Theta(max)= 28.323

                                                 wR2(reflections)=
R(reflections)= 0.0393( 2963)                    0.1062( 3225)

S = 1.037                  Npar= 163
```

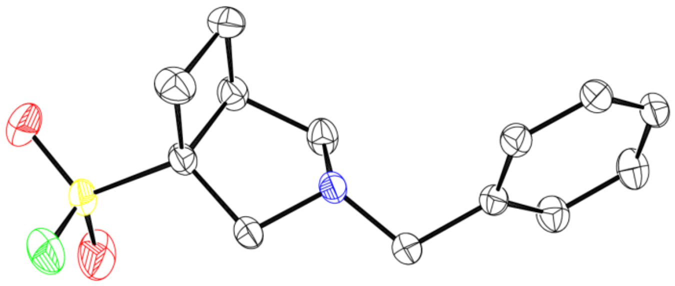

ORTEP representation of the molecular structure with displacement ellipsoids drawn at the 50% probability level.

5) References

[1] M. A. C. H. Janssen, R. Rappard, T. Dekker, M. Heiming, M. Beens, D. Pieters, B. H. M. Kuijpers, J. C. J. Benningshof, M. Wijtmans, I. J. P. de Esch, D. Blanco-Ania, F. P. J. T. Rutjes, *Eur. J. Org. Chem.* **2024**, e202400797.

[2] A. A. Homon, O. V. Hryshchuk, S. Trofymchuk, O. Michurin, Y. Kuchkovska, D. S. Radchenko, O. O. Grygorenko, *Eur. J. Org. Chem.* **2018**, *2018*, 5596–5604.

[3] S. Mahapatra, C. P. Woroch, T. W. Butler, S. N. Carneiro, S. C. Kwan, S. R. Khasnavis, J. Gu, J. K. Dutra, B. C. Vetelino, J. Bellenger, C. W. am Ende, N. D. Ball, *Org. Lett.* **2020**, *22*, 4389–4394.

[4] B. V. Yang, D. O'Rourke, J. Li, *Synlett* **1993**, *1993*, 195–196.

[5] Y. Hara, S. Onodera, T. Kochi, F. Kakiuchi, *Org. Lett.* **2015**, *17*, 4850–4853.

[6] C. L. Cioffi, N. Dobri, E. E. Freeman, M. P. Conlon, P. Chen, D. G. Stafford, D. M. C. Schwarz, K. C. Golden, L. Zhu, D. B. Kitchen, K. D. Barnes, B. Racz, Q. Qin, E. Michelotti, C. L. Cywin, W. H. Martin, P. G. Pearson, G. Johnson, K. Petrukhin, *J. Med. Chem.* **2014**, *57*, 7731–7757.

[7] B.-S. Kim, J. Jiménez, F. Gao, P. J. Walsh, *Organic Letters* **2015**, *17*, 5788–5791.

[8] R. Dorel, C. P. Grugel, A. M. Haydl, *Angew. Chem. Int. Ed.* **2019**, *58*, 17118–17129.

[9] A. F. Abdel-Magid, K. G. Carson, B. D. Harris, C. A. Maryanoff, R. D. Shah, *J. Org. Chem.* **1996**, *61*, 3849–3862.

[10] M. F. Khumalo, E. D. Akpan, P. K. Chinthakindi, Edikarlos M. Brasil, K. K. Rajbongshi, M. M. Makatini, T. Govender, H. G. Kruger, T. Naicker, P. I. Arvidsson, *RSC Adv.* **2018**, *8*, 37503-37507.

[11] M. Wei, D. Liang, X. Cao, W. Luo, G. Ma, Z. Liu, L. Li, *Angew. Chem. Int. Ed.* **2021**, *60*, 7397-7404.
